# Acute caffeine ingestion and exercise performance in women: a systematic review and meta-analysis of menstrual-cycle phase and hormonal contraceptive status

**DOI:** 10.3389/fnut.2026.1876198

**Published:** 2026-07-10

**Authors:** Jian Zhu, Yinghui Zhang, Ming Li, Xiaohan Fan, Ping Liu, Hengzhi Deng

**Affiliations:** 1 School of Physical Education, Huaqiao University, Quanzhou, China; 2 School of Arts and Sciences, Fujian Fuyao University of Science and Technology, Fuzhou, China; 3 Faculty of Sports and Exercise Science, University of Malaya, Kuala Lumpur, Malaysia

**Keywords:** caffeine, exercise performance, hormonal contraceptives, menstrual cycle, women

## Abstract

**Background:**

Acute caffeine ingestion is widely used to enhance exercise performance, but most evidence comes from male or mixed-sex cohorts. Whether menstrual-cycle phase or hormonal contraceptive status modifies caffeine's ergogenic effect in women remains unclear.

**Methods:**

Six databases were searched for randomized controlled trials examining acute caffeine ingestion and objective exercise performance outcomes in women. Methodological quality and risk of bias were assessed using a modified PEDro scale and RoB 2. Three-level meta-analyses accounted for dependent effect sizes within studies. The primary moderator was reproductive-hormonal status, classified as early follicular, late follicular/peri-ovulatory, luteal/mid-luteal, or hormonal contraceptive/oral contraceptive use. Exploratory analyses examined dose, exercise type, phase verification, timing, and habitual caffeine intake.

**Results:**

Twenty studies contributed 144 primary effect sizes. Acute caffeine ingestion improved exercise performance in women overall (Hedges' g = 0.37, 95% CI 0.24 to 0.50), with moderate heterogeneity and a prediction interval crossing the null. Positive estimates were observed across all reproductive-hormonal strata, but there was no clear evidence of between-stratum moderation. The overall effect was robust to outlier exclusion and alternative correlation assumptions. Exploratory analyses suggested that exercise-task phenotype and phase-verification method may partly explain variability in observed effects, whereas dose, timing, and habitual caffeine intake did not support phase-specific prescriptions. Risk-of-bias concerns, sparse subgroup data, and low-to-very-low GRADE certainty limited individualized inference.

**Conclusions:**

Acute caffeine ingestion produces a small average ergogenic effect in women, but exploratory findings suggest that its expression may vary by exercise task within different reproductive-hormonal contexts. Current evidence therefore supports a context-sensitive interpretation rather than rigid menstrual-phase- or contraceptive-specific prescriptions. However, given the low-to-very-low certainty of evidence, these findings should be interpreted cautiously and considered hypothesis-generating. Inconsistent phase verification remains a key methodological limitation that may obscure biologically meaningful patterns. Future female-specific trials should combine rigorous hormonal verification, detailed contraceptive profiling, prespecified exercise phenotypes, and mechanistic assessment to refine individualized caffeine guidance..

**Systematic review registration:**

OSF https://osf.io/5y69g/, identifier 5y69g.

## Graphical Abstract

**Figure:**
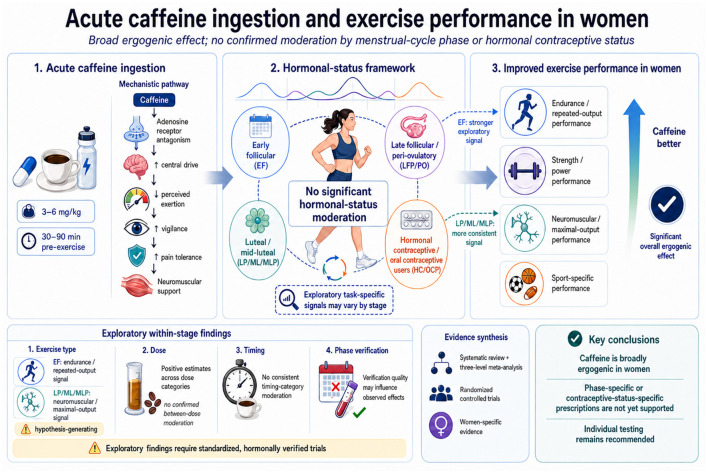

## Highlights

- Acute caffeine ingestion may associate with small-to-moderate improvements in exercise performance in women.
- No robust evidence supports differential ergogenic effects across menstrual-cycle phases or hormonal contraceptive status.
- Exploratory within-stage analyses suggest that exercise-task phenotype may help explain variability in caffeine responsiveness, but this evidence remains hypothesis-generating.
- Variability in responses may be influenced by hormonal context, although current evidence remains insufficient to confirm this.
- Current evidence does not support phase-specific supplementation strategies, highlighting the need for well-controlled, hormonally verified trials.

## 1 Introduction

Caffeine is one of the most widely used ergogenic aids in sport, with meta-analytic evidence supporting small-to-moderate benefits for endurance, muscular endurance, strength, power, and sport-specific performance (1). However, much of this evidence has been derived from male or mixed-sex samples, leaving uncertainty about the magnitude and consistency of caffeine's effects in women (2, 3). This evidence gap is important because female hormonal fluctuations may influence exercise responses, substrate metabolism, neuromuscular function, and pharmacological responses to caffeine (4).

Menstrual-cycle phase and hormonal contraceptive use are increasingly recognized as key methodological and biological variables in women's sport and exercise research (4). Although recent meta-analyses suggest that menstrual-cycle phase and oral contraceptive use have, on average, trivial effects on exercise performance at the group level, substantial heterogeneity and inter-individual variability remain (5, 6). These issues are particularly relevant for caffeine research, where modest ergogenic effects may be obscured when female hormonal status is poorly classified, inconsistently verified, or not considered analytically.

There is also a plausible biological rationale for examining hormonal status as a moderator of caffeine's ergogenic effects. Caffeine is primarily metabolized through hepatic CYP1A2, and oral contraceptive steroids have been shown to impair caffeine elimination and prolong caffeine half-life (7). In contrast, CYP1A2-dependent caffeine metabolism appears to vary minimally across the natural menstrual cycle, suggesting that exogenous hormonal contraceptive use may have a more consistent pharmacokinetic influence than menstrual-cycle phase alone (8, 9). Nevertheless, both endogenous and exogenous hormonal environments may still affect caffeine exposure, tolerability, and performance responses.

Despite this biological rationale, existing evidence in women has primarily focused on the overall ergogenic effects of caffeine rather than on whether these effects differ across hormonal contexts. Female-specific caffeine reviews generally report beneficial effects on muscular strength, muscular endurance, jumping performance, and team-sport-related outcomes (10–12). Individual randomized trials have reported improvements in Wingate performance, peak aerobic cycling power, movement velocity, strength, and power outcomes in women (13–17). However, findings are not uniform, with recent evidence showing no clear benefit of caffeine for 5-km cycling time-trial performance across menstrual-cycle phases (18), and these results are rarely interpreted within a consistent hormonal framework.

Taken together, caffeine appears ergogenic in women, but whether its effects differ by menstrual-cycle phase or hormonal contraceptive status remains unclear. This review therefore synthesized randomized trial evidence on acute caffeine ingestion and objective exercise performance in women, using reproductive-hormonal status as the primary analytical framework.

## 2 Methods

This systematic review and meta-analysis was pre-registered on the Open Science Framework (OSF) on March 16, 2026 (Registration: osf.io/5y69g) and conducted in accordance with the PRISMA 2020 guidelines (19). The registered protocol and dataset used for the present meta-analysis are available in the OSF repository. The completed PRISMA checklist is available in Supplementary material Appendix S1.

### 2.1 Eligibility criteria

Eligibility criteria were prespecified according to the PICOS framework and reported in accordance with the PRISMA 2020 statement (19). Studies were considered eligible if they met all of the following criteria: (1) Participants: healthy women aged 18 years or older, including either naturally menstruating/eumenorrheic women or women using hormonal contraceptives/oral contraceptives; studies enrolling mixed-sex samples were eligible only when female-specific data were reported separately or could be extracted separately; (2) Intervention: acute pre-exercise caffeine ingestion administered alone in an identifiable dose, whether as anhydrous caffeine or a caffeine-containing beverage/solution; (3) Comparator: placebo, non-caffeinated control, or a matched control condition; (4) Outcomes: objective exercise performance outcomes, including endurance, muscular endurance, strength, power and sport-specific performance; and (5) Study design: randomized controlled trials, including crossover and parallel-group designs, published as full-text articles in peer-reviewed journals in English.

To address the primary aim of this review, eligible studies were also required to provide sufficient information to classify participants according to menstrual-cycle phase and/or hormonal contraceptive status, or to report extractable subgroup data that allowed such classification.

Studies were excluded if they met any of the following criteria: (1) included men only, clinical populations, pregnant or postpartum women, or mixed-sex samples from which female-specific data could not be separated; (2) evaluated chronic or repeated caffeine supplementation rather than an acute pre-exercise intervention; (3) administered caffeine in combination with other active ergogenic ingredients such that the independent effect of caffeine could not be isolated; (4) used non-ingestive strategies, such as caffeine mouth rinsing, or other routes not consistent with acute caffeine ingestion; (5) lacked a placebo/control condition; (6) did not report objective exercise performance outcomes; (7) did not report, or did not allow classification of, menstrual-cycle phase or hormonal contraceptive status; or (8) were reviews, meta-analyses, conference abstracts, dissertations, study protocols, case reports, duplicate publications, or reports with insufficient data for extraction.

### 2.2 Data sources and search strategy

The systematic search process was initiated in March 2026 alongside protocol development and preliminary study screening. To ensure that the evidence base was current before manuscript submission, an updated search was completed on May 1, 2026 across PubMed, Web of Science, Cochrane Library, Embase, SciELO, and SPORTDiscus. The PRISMA flow diagram was prepared using the results of this updated search. One Boolean strategy was applied:

(“caffeine” OR “anhydrous caffeine” OR “caffeine supplementation” OR “caffeine ingestion” OR “acute caffeine ingestion”) AND (“female” OR “women” OR “woman” OR “female athlete” OR “female athletes” OR “sportswomen”) AND (“exercise performance” OR “time trial” OR “time to exhaustion” OR “Wingate test” OR “countermovement jump” OR “CMJ” OR “1RM” OR “one repetition maximum” OR “bench press” OR “sprint performance” OR “VO2max” OR “maximal oxygen uptake”) AND (“randomized controlled trial” OR “randomized controlled trial” OR “randomized trial” OR “randomized trial” OR “placebo controlled” OR “placebo-controlled” OR “crossover trial” OR “cross-over trial” OR “double blind”).

No date or filter restrictions were applied.

### 2.3 Data extraction

All records were imported into Excel and EndNote 25 for de-duplication. Two independent reviewers (Z.J. and D.Z.H.) screened titles, abstracts, and full texts. Discrepancies were resolved by consensus. Extracted data included sample size, participant characteristics, training status, caffeine dose and form, timing of ingestion, placebo/control condition, menstrual cycle phase and/or hormonal contraceptive status, exercise protocol, and direct exercise-performance outcomes (e.g., time trial, time to exhaustion, Wingate test, countermovement jump, 1RM, bench press, sprint performance, and VO2max). Only outcomes directly reflecting exercise performance were eligible for quantitative synthesis to improve outcome comparability across studies; derived metrics that did not directly represent exercise performance were not included in the primary meta-analysis.

When data were not reported numerically, authors were contacted or WebPlotDigitizer (v4.8) was used for extraction (20).

### 2.4 Quality and risk of bias assessment

Methodological quality was assessed using a modified version of the Physiotherapy Evidence Database (PEDro) scale, with the inclusion of an additional item evaluating whether the effectiveness of blinding to the placebo condition was assessed (1). The total score ranged from 0 to 12, and studies were classified as excellent (10–12), good (7–9), fair (5, 6), or poor (<5).

Risk of bias was evaluated using the Cochrane Risk of Bias 2 (RoB 2) tool with design-specific application. Because the final evidence base included both crossover and parallel-group randomized trials, the version of RoB 2 adapted for crossover trials was applied to crossover studies, whereas the standard RoB 2 tool was applied to the single parallel-group trial. Specifically, the crossover version included the additional domain addressing bias arising from period effects and carryover effects, in addition to the five standard domains: bias arising from the randomization process, deviations from intended interventions, missing outcome data, measurement of the outcome, and selection of the reported result (21). For the parallel-group trial, the crossover-specific domain was considered not applicable.

All assessments were conducted independently by the same two reviewers (Z.J. and D.H.Z.), with discrepancies resolved through consensus or adjudication by a third reviewer when required (Z.Y.H.).

### 2.5 Statistical analysis

#### 2.5.1 Effect size calculation

The effects of acute caffeine ingestion on female exercise performance were synthesized using standardized mean differences (SMDs), expressed as Hedges' *g*, to correct for small-sample bias (22). For all outcomes, effect directions were harmonized so that positive effect sizes consistently indicated better performance with caffeine than with placebo or control.

Because the final evidence base consisted predominantly of crossover trials, within-subject dependency was considered in the calculation of effect sizes and sampling variances whenever possible (23, 24). For crossover studies, an assumed within-subject correlation of *r* = 0.50 was used in the primary analysis when the required correlation was not reported (25). To evaluate the robustness of the pooled estimates to this assumption, sensitivity analyses were additionally performed using *r* = 0.20 and *r* = 0.80. For the single parallel-group trial, effect sizes were calculated using the standard between-group contrast (23, 25).

Effect sizes were (g) interpreted using standard thresholds: trivial (<0.2), small (0.2–0.5), medium (0.5–0.8), and large (>0.8) (26). Detailed computational formulas and step-by-step procedures are provided in (Supplementary material Appendix S2).

#### 2.5.2 Three-level meta-analysis and heterogeneity

To account for multiple effect sizes nested within studies, three-level random-effects meta-analyses were performed using the metafor package in R with restricted maximum likelihood estimation (REML) (27–29). Variance was partitioned into sampling error (level 1), within-study variance (level 2), and between-study variance (level 3) (28, 29). Model estimates were checked using maximum likelihood (ML).

Heterogeneity was assessed using total I^2^ and variance components, with I^2^ interpreted as low (0–25%), moderate (25–50%), substantial (50–75%), or considerable (>75%) (30, 31). Prediction intervals (PIs) were calculated to indicate the expected range of true effects in future comparable studies (32, 33). *Post-hoc* power was examined descriptively as a supplementary diagnostic for potential Type II error (34, 35).

#### 2.5.3 Moderator and exploratory analyses

To explore heterogeneity in the ergogenic response to acute caffeine ingestion, moderator and subgroup analyses were conducted for exercise-performance outcomes.

The primary moderator was menstrual-cycle phase or hormonal contraceptive status. Effect sizes were classified into four categories: early follicular phase (EF), late follicular/peri-ovulatory phase (LFP/PO), luteal/mid-luteal phase (LP/ML/MLP), and hormonal contraceptive/oral contraceptive users (HC/OCP), in accordance with methodological recommendations for exercise studies involving women (4, 36). A mixed-effects three-level meta-regression model was fitted with this four-level variable as a categorical moderator. Subgroup-specific estimates were obtained using a no-intercept model, and between-category differences were evaluated using omnibus moderator tests.

For exploratory within-stage analyses, the following moderators were examined:

**1. Caffeine dose**. Acute caffeine dose was categorized as 3 mg/kg, 4 mg/kg, or ≥5 mg/kg body mass, reflecting the dose distribution in the included studies and the commonly reported ergogenic range of 3–6 mg/kg (37).

**2. Exercise task type**. Outcomes were grouped into endurance/repeated-output performance and neuromuscular/maximal-output performance. The former included time trials, time-to-exhaustion tests, repetitions to failure, total-volume outcomes, and sport-specific repeated-output tasks; the latter included maximal strength, jump, sprint/agility, Wingate-derived power, movement velocity, and maximal voluntary contraction outcomes.

**3. Phase-verification method**. Phase classification was grouped as no verification/cycle counting or hormonal verification. The no verification/cycle-counting category included self-report, calendar-based estimation, period tracking, pill-cycle timing, unclear verification, or no formal phase control; hormonal verification included luteinizing hormone testing, basal body temperature assessment, or blood-based hormonal confirmation (4, 36).

**4. Caffeine-ingestion timing**. Timing was analyzed according to the reported interval between caffeine ingestion and exercise testing. Categorical timing analyses were conducted where sufficient data were available, and continuous timing meta-regression was performed as an exploratory analysis.

**5. Habitual caffeine intake**. Daily caffeine intake was classified as low (0–150 mg/day), moderate (150–300 mg/day), or high (>300 mg/day) (38). When intake was not reported or could not be reliably classified, it was coded as unclear.

Because data were unevenly distributed across reproductive strata, the main exploratory within-stage analyses focused on EF and LP/ML/MLP, where sufficient data were available. Analyses in LFP/PO and HC/OCP were treated as sparse-strata exploratory analyses and interpreted descriptively when models were unstable, based on few studies or effect sizes, or not estimable.

#### 2.5.4 Publication bias and sensitivity analyses

Contour-enhanced funnel plots (39) and Egger's regression tests (40) were used to assess potential publication bias when at least 10 effect sizes were available for a given analysis (41).

Sensitivity analyses were conducted to evaluate the robustness of the main findings. For the overall pooled effect and the primary menstrual-cycle/contraceptive subgroup analyses, sensitivity analyses included: 1) varying the assumed within-participant correlation coefficients using a relatively low value (r = 0.2) and a relatively high value (r = 0.8); 2) leave-one-out analyses; and 3) exclusion of outliers identified using Cook's distance and studentized residuals (42, 43). For the exploratory nested subgroup analyses, outlier-exclusion sensitivity analyses were additionally performed to assess whether subgroup-level inferences were robust to influential effects. Other sensitivity checks were restricted to the main pooled and primary subgroup models.

### 2.6 Certainty of the evidence

The certainty of evidence was evaluated using the Grading of Recommendations Assessment, Development, and Evaluation (GRADE) framework, considering risk of bias, inconsistency, indirectness, imprecision, and publication bias (44). Ratings were categorized as high, moderate, low, or very low. All GRADE assessments were performed independently by one reviewer and verified by a second. Any disagreements were resolved through discussion until consensus was reached.

## 3 Results

### 3.1 Studies retrieved

**Figure 1:**
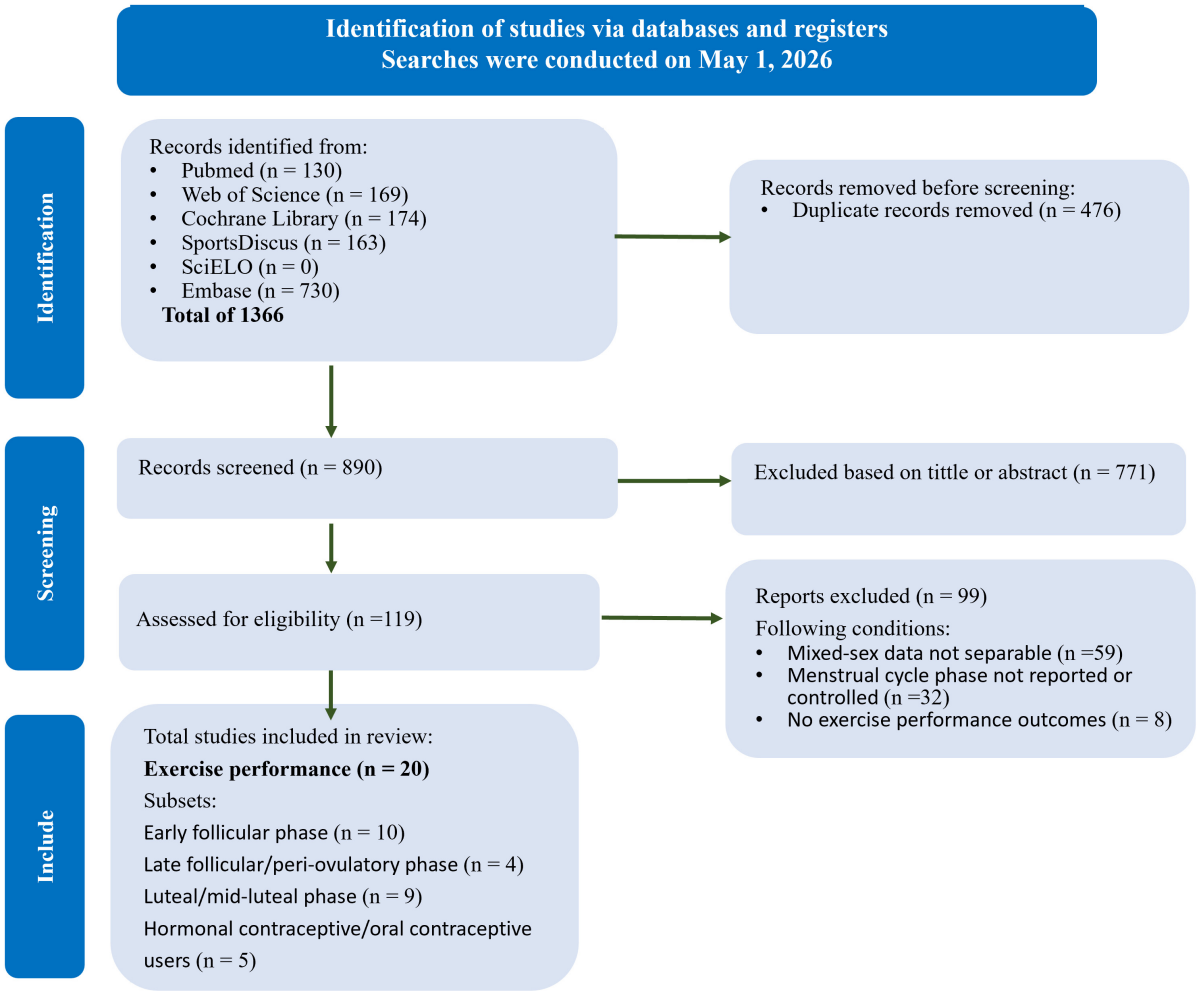
PRISMA flow diagram for included and excluded studies.

The initial search yielded 1,366 publications from the primary database search. After screening, a total of 20 studies met the inclusion criteria. These studies provided 144 eligible exercise-performance effect size estimates (k = 144) for the primary meta-analysis. Across reproductive-hormonal strata, 10 studies contributed data from the early follicular phase (k = 44), 4 studies from the late follicular/peri-ovulatory phase (k = 14), 9 studies from the luteal/mid-luteal phase (k = 55), and 5 studies from hormonal contraceptive/oral contraceptive users (k = 31) (Figure 1).

### 3.2 Characteristics of included studies

The 20 included studies represented 272 female participants, with study-level sample sizes ranging from 10 to 26. Most studies adopted randomized, double-blind, placebo-controlled crossover designs; the exceptions were one randomized, triple-blind, placebo-controlled crossover trial (45) and one randomized parallel-group trial (18). Participants were predominantly trained or physically active women, with 17 studies contributing data from trained participants (k = 136) and 3 studies contributing data from untrained or recreationally active participants (k = 8).

Regarding reproductive-hormonal status, studies included women tested during different menstrual-cycle phases as well as hormonal contraceptive/oral contraceptive users. Phase classification was based on no verification/cycle counting in 15 studies (k = 108) and hormonal verification in 5 studies (k = 36). Caffeine was administered acutely at doses ranging from 3 to 6 mg/kg body mass, with most effect sizes involving 3 mg/kg caffeine (12 studies, k = 81), followed by ≥5 mg/kg (7 studies, k = 49) and 4 mg/kg (3 studies, k = 14). The retained outcomes were classified as neuromuscular/maximal-output performance (14 studies, k = 100) or endurance/repeated-output performance (15 studies, k = 44). Capsules were the most common delivery form (13 studies, k = 94), followed by oral supplements (3 studies, k = 25), drinks (3 studies, k = 24), and coffee (1 study, k = 1). The interval between caffeine ingestion and exercise testing ranged from 30 to 90 min, with 60 min being the predominant protocol (16 studies, k = 119). Habitual caffeine intake was mainly classified as low (14 studies, k = 102), with fewer studies involving moderate intake (5 studies, k = 35) or high intake (1 study, k = 7).

For more details, please refer to Table 1.

**Table 1:** Summary of studies assessing the effect of caffeine ingestion on exercise performance in female participants.

Study; Study design	Exercise protocol	Sample; Training status	Mean age (y); Body weight (kg); Daily caffeine intake	Dosage of CAF and PLA; Form, timing; Menstrual period; Phase verification	Washout period	Performance outcomes	Statistical significance
Abumoh'd et al. (57) RDB Crossover	3 × 10 reps at 60% 1-RM Chest press/Lat pulldown/Triceps pushdown and Rowing torso +4th set: RTF	10 F; Resistance-trained (training exp: 4.33 ± 3.34 y)	24.53 ± 4.11; 58.42 ± 3.63; ≤ 200 mg/day	CAF: 4 mg/kg; PLA: placebo; Capsule, 60 min; EF; Self-report	48 h	RTF (reps) of Chest press/Lat pulldown/Triceps pushdown and Rowing torso: CAF: 23.12 ± 2.29/18.33 ± 3.28/22.77 ± 1.77 and 20.63 ± 2.07 vs. PLA: 21.45 ± 3.11/18.83 ± 4.31/20.21 ± 1.89 and 18.84 ± 3.22; Total repetitions (reps): CAF: 84.85 ± 5.66 vs. PLA: 79.33 ± 6.43	Performance: Yes, only with total repetitions: ↑ (p < 0.05)
Ali et al. (13) RDB Crossover	90-min intermittent treadmill-running protocol; Knee Flexor/Extensor isometric, concentric and eccentric tests + CMJ; Trial Mean only	10 F; team-sport players (2–6 sessions/week; recreational to international level)	24 ± 4; 59.7 ± 3.5; 0–300 mg/day	CAF: 6 mg/kg; PLA: artificial sweetener; Capsule, 60 min; OCP (days 5–8 and 18–22); Pill-cycle timing + pill diary	≈10–17 d	Pre- and mid/post -eccentric knee flexor torque (Nm): CAF: 170.0 ± 55.0 and 183.0 ± 46.0/170.0 ± 55.0 and 183.0 ± 52.0 vs. PLA: 159.0 ± 39.0 and 155.0 ± 43.0/159.0 ± 39.0 and 165.0 ± 53.0; Pre- and mid/post -eccentric knee extensor torque (Nm): CAF: 119.0 ± 38.0 and 123.0 ± 41.0/119.0 ± 38.0 and 122.0 ± 43.0 vs. PLA: 116.0 ± 31.0 and 106.0 ± 30.0/116.0 ± 31.0 and 115.0 ± 38.0; Pre- and mid/post -eccentric knee flexor power (W): CAF: 39.0 ± 14.0 and 41.0 ± 10.0/39.0 ± 14.0 and 41.0 ± 14.0 vs. PLA: 36.0 ± 11.0 and 36.0 ± 11.0/38.0 ± 12.0; Pre- and mid/post -eccentric knee extensor power (W): CAF: 30.8 ± 9.6 and 30.8 ± 9.8/30.8 ± 9.6 and 29.2 ± 9.4 vs. PLA: 29.5 ± 9.0 and 26.8 ± 7.8/29.5 ± 9.0 and 27.8 ± 6.8; Pre- and mid/post -concentric knee flexor torque (Nm): CAF: 75.9 ± 19.8 and 80.6 ± 17.3/5.9 ± 19.8 and 80.2 ± 20.1 vs. PLA: 79.7 ± 21.2 and 77.9 ± 23.4/79.7 ± 21.2 and 77.1 ± 20.8; Pre- and mid/post -concentric knee extensor torque (Nm): CAF: 112.6 ± 45.3 and 115.7 ± 33.7/112.6 ± 45.3 and 114.4 ± 32.3 vs. PLA: 104.4 ± 34.6 and 105.0 ± 35.7/104.4 ± 34.6 and 111.8 ± 43.2; Pre- and mid/post -concentric knee flexor power (W): CAF: 24.8 ± 7.1 and 24.5 ± 7.4/24.8 ± 7.1 and 24.5 ± 5.8 vs. PLA: 25.7 ± 7.5 and 23.7 ± 6.5/25.7 ± 7.5 and 24.0 ± 7.1 Pre- and mid/post -concentric knee extensor power (W): CAF: 23.1 ± 8.0 and 26.7 ± 8.0/23.1 ± 8.0 and 27.0 ± 8.1 vs. PLA: 24.3 ± 8.7 and 23.8 ± 9.0/24.3 ± 8.7 and 25.5 ± 9.2; Pre- and mid/post -isometric knee flexor torque (Nm): CAF: 77.9 ± 18.3 and 79.9 ± 24.9/77.9 ± 18.3 and 81.7 ± 17.0 vs. PLA: 77.6 ± 18.6 and 77.0 ± 16.9/77.6 ± 18.6 and 76.3 ± 17.5; Pre- and mid/post -isometric knee extensor torque (Nm): CAF: 153.6 ± 47.7 and 151.8 ± 30.0/153.6 ± 47.7 and 159.9 ± 44.0 vs. PLA: 157.4 ± 40.8 and 155.3 ± 38.9/157.4 ± 40.8 and 156.1 ± 35.1; Trial mean CMJ Height (cm)/Estimated CMJ Power (W): PLA: 39.7 ± 5.7/3055.0 ± 403.7 vs. CAF: 40.1 ± 4.7/3089.3 ± 390.8	Performance: Yes, only with ecc knee flexor strength/flexor power/extensor power: ↑ (*p* < 0.05)
Chen et al. (58) RDB Crossover	MVIC; Tlim at 50% MVIC; MVICpost	10 F; elite collegiate athletes (basketball/ soccer/tennis)	19.90 ± 0.99; 60.75 ± 7.89; < 200 mg/week	CAF: 6 mg/kg; PLA: diet flour; capsule, 60 min; EF; self-report	1 week	Pre- and post-MVIC (Nm/kg): CAF: 4.0 ± 0.5 and 3.6 ± 0.7 vs. PLA: 3.7 ± 0.6 and 3.4 ± 0.6. Tlim (s): CAF: 89 ± 14 vs. PLA: 77 ± 13; MVICpost (Nm/kg): CAF: 3.6 ± 0.7 vs. PLA: 3.4 ± 0.6;	Performance: Yes, only with MVIC, Tlim and MVICpost; ↑ (*p* < 0.05)
Clarke et al. (59) RDB Crossover	5-km cycling TT	19 F; Highly active	28 ± 6; 72 ± 11; 214 ± 158 mg/day	CAF: 3 mg/kg; PLA: placebo; Coffee; 60 min; OCP (days 5–8 and 19–22); Pill-cycle timing	≥48 h	5-km TT time (s): CAF: 516 ± 42; PLA: 525 ± 47; CON: 522 ± 45	Performance: Yes, only with CAF vs. PLA/CAF vs. CON: ↓ (*p* < 0.05)
Jones et al. (46) RDB Crossover	Leg press 1RM Leg press RF at 60% 1RM Total volume lifted	14 F; strength-trained (4.0 ± 1.0 sessions/week)	23.3 ± 3.9; 64.1 ± 10.4; 109.7 ± 73.4 mg/day	CAF: 3 and 6 mg/kg; PLA: placebo; Drink, 30 min; HC; Not phase-controlled	≥ 3 d	1RM (kg): 3CAF/6CAF/PLA: 266.46 ± 56.03/270.46 ± 51.95/267.77 ± 52.86 RF (reps): 3CAF/6CAF: 43.69 ± 16.50/48.23 ± 23.84/35.46 ± 11.98; Total volume lifted (kg): 3CAF/6CAF/PLA: 6,280 ± 2,210; 6,840 ± 3,360/4,940 ± 1,450	Performance: yes, only with RF and total volume lifted: ↑ (*p* < 0.05)
Karayigit et al. (60) RDB Crossover	3 sets of Squat/Bench Press RTF at 40% 1RM;	17 F; resistance-trained athletes (RT exp: 4 ± 1 y; caffeine-naive)	23 ± 2; 64 ± 4; 15 ± 4 mg/day	CAF: 3 and 6 mg/kg; PLA: decaf coffee; Drink, 60 min; LP; Period tracker + self-report	48–72 h	3 sets of squat repetitions (reps): PLA/3CAF/6CAF: 31.2 ± 5.1, 24.5 ± 4.6, 13.1 ± 5.2/33.4 ± 5.2, 24.4 ± 4.8, 13.5 ± 4.6/33.9 ± 6.0, 24.5 ± 4.3, 13.2 ± 4.0; 3 sets of bench press repetitions (reps): PLA/3CAF/6CAF: 24.6 ± 4.4, 17.8 ± 3.5, 12.9 ± 3.2/24.7 ± 4.7, 17.9 ± 3.8, 12.9 ± 3.2/25.2 ± 5.1, 17.5 ± 4.0, 12.3 ± 3.0	Performance: yes, only for Squat 1st set: ↑ (*p* < 0.05)
Lara et al. (16) RDB Crossover	Incremental maximal cycle ergometer test; Wmax	13 F; well-trained triathletes (~2 h/day; ≥5 d/week)	31 ± 6; 58.6 ± 7.8; < 50 mg/day	CAF: 3 mg/kg; PLA: cellulose; Capsule, 60 min; EF/PO/ML; Tracker app + LH test + BTT	≥48 h	Wmax (W/kg): EF/PO/ML: CAF 4.24 ± 0.71/4.27 ± 0.73/4.29 ± 0.67 vs. PLA 4.13 ± 0.69/4.14 ± 0.70/4.15 ± 0.69	Performance: yes ↑ (*p* < 0.05)
Lara et al. (14, 15) RDB Crossover	15-s adapted Wingate test Cycling	13 F; well-trained triathletes (~2 h/day; ≥5 d/week)	31 ± 6; 58.6 ± 7.8; < 50 mg/day	CAF: 3 mg/kg; PLA: cellulose; Capsule, 60 min; EF/PO/ML; Tracker app + LH test + BTT	48 h	Peak power (W/kg): EF/PO/ML: CAF 8.9 ± 0.9/8.9 ± 0.9/8.9 ± 0.8 vs. PLA 8.6 ± 0.8/8.6 ± 0.9/8.6 ± 0.8 Mean power (W/kg):EF/PO/ML: CAF 7.9 ± 0.7/8.0 ± 0.8/8.2 ± 0.7 vs. PLA 7.8 ± 0.8/7.8 ± 0.9/8.0 ± 0.7	Performance: yes ↑ (*p* < 0.05)
Lara et al. (61) RDB Crossover	15-s adapted Wingate test	10 F; intermittent-sport athletes (>1 h/day; ≥4 d/week for previous year)	30.8 ± 5.4 57.3 ± 7.4 < 60 mg/day	CAF: 3 mg/kg PLA: cellulose Capsule, 60 min; ML; Tracker app + LH test + BTT	1 week	Peak power (W/kg): CAF: 9.1 ± 0.8 vs. PLA: 8.8 ± 0.9 Mean power (W/kg): CAF: 8.3 ± 0.7 vs. PLA: 8.1 ± 0.7	Performance: yes ↑ (*p* < 0.05)
Mendes et al. (18) RDB Parallel	5-km cycling TT	21 F; exercise practitioners (regular PA: 150–300 min/week)	26.6 ± 3.97; 61.6 ± 8.37; 119 ± 97.7 mg/day	CAF: 6 mg/kg; PLA: maltodextrin; Capsule, 60 min; LFP/MLP; Forward-count method	NA	5-km time (min): LFP/MLP: CAF 10.01 ± 1.46/9.95 ± 1.51 vs. PLA 10.5 ± 1.09/10.6 ± 0.90; Mean power (W): LFP/MLP: CAF 117 ± 41.6/122 ± 48.3 vs. PLA 98.1 ± 29.9/99.9 ± 32.5; Mean power (W/kg): LFP/MLP: CAF 1.94 ± 0.63/2.05 ± 0.70 vs. PLA 1.56 ± 0.41/1.59 ± 0.38;	Performance: No (*p* > 0.05)
Norum et al. (62) RDB Crossover	CMJ; MVC knee extension; Squat 1RM/RTF at 60% 1RM; Bench press 1RM/RTF at 60% 1RM	15 F; resistance-trained (≥12 mo; 2–3 sessions/week)	29.8 ± 5.5; 63.8 ± 5.5; 341 ± 184 mg/day	CAF: 4 mg/kg; PLA: placebo; Oral supplement, 60 min; EF; self-report	72 h	CMJ height (cm): CAF: 34.3 ± 4.5 vs. PLA: 32.0 ± 4.7; CMJ peak power (W): CAF: 2946 ± 430 vs. PLA: 2840 ± 430; MVC peak torque (Nm): CAF: 181 ± 31 vs. PLA: 173 ± 29; Squat 1RM (kg)/RTF (reps): CAF: 100 ± 13/45 ± 17 vs. PLA: 96 ± 14/39 ± 17; Bench press 1RM (kg)/RTF (reps): CAF: 68 ± 11/23 ± 6 vs. PLA: 66 ± 10/21 ± 6	Performance: yes ↑ (*p* < 0.005)
Ouergui et al. (63) RDB Crossover	TSAT; FSKT-10s; FSKT-mult	10 F (subgroup; *n* = 20 total); taekwondo athletes (≥6 y experience)	17.5 ± 0.7; 59.2 ± 10.0; moderate consumers (< 3 cups/day)	CAF: 3 mg/kg; PLA: placebo; Drink, 60 min; EF; self-report	1 week	TSAT (s): PL + NoCA: 6.6 ± 0.5 vs. CAF + NoCA: 6.3 ± 0.4; PL + CA: 6.2 ± 0.4 vs. CAF + CA: 5.8 ± 0.4 FSKT-10s (n): PL + NoCA: 23 ± 1 vs. CAF + NoCA: 24 ± 1; PL + CA: 24 ± 1 vs. CAF + CA: 26 ± 1 FSKT-mult (n): PL + NoCA: 105 ± 6 vs. CAF + NoCA: 110 ± 10; PL + CA: 111 ± 9 vs. CAF + CA: 119 ± 6	Performance: yes (*p* < 0.005)
Ouergui et al. (64) RDB Crossover	TSAT; FSKT-10s; FSKT-multi	26 F; taekwondo athletes (elite n = 16; sub-elite n = 10)	Elite F: 17.7 ± 0.6 Sub-elite F: 17.3 ± 0.5 Elite F: 50 ± 5 kg Sub-elite F: 55 ± 8 kg 1.14 ± 0.49 mg/kg/day	CAF: 3 mg/kg; PLA: placebo; Oral supplement, 45 min; EF; period tracker + self-reported	1 week	TSAT (s): Elite F/Sub-elite F: CAF 5.5 ± 0.3/6.3 ± 0.4 vs. PLA 5.8 ± 0.3/6.6 ± 0.5; FSKT-10s (n): Elite F/Sub-elite F: CAF 27 ± 2/24 ± 1 vs. PLA 25 ± 1/23 ± 1; FSKT-multi (*n*): Elite F/Sub-elite F: CAF 127 ± 2/110 ± 10 vs. PLA 124 ± 1/105 ± 6	Performance: yes (*p* < 0.005)
Robles-González et al. (45) TB crossover	CMJ; BPT peak velocity; squat 1RM/BP 1RM; squat AV-8R at 70% 1RM/BP RtF at 70% 1RM	15 F; resistance-trained (≥1 y; ≥2 sessions/week; habitual caffeine >2 mg/kg/day)	25.1 ± 4.3; 62.8 ± 9.5; 121.4 ± 82.5 mg/day	CAF: 3 mg/kg; PLA: microcrystalline cellulose; Oral supplement, 30 min; LP; Not reported	48–72 h	CMJ (cm): AM/PM: CAF: 27.59 ± 5.06/28.20 ± 5.65 vs. PLA: 26.75 ± 4.66/27.74 ± 4.81; BPT peak velocity (m/s):AM/PM: CAF: 1.67 ± 0.34/1.70 ± 0.37 vs. PLA: 1.65 ± 0.34/1.69 ± 0.37; Squat 1RM/BP 1RM (kg): AM: CAF: 72.01 ± 10.62/43.06 ± 9.57 vs. PLA: 71.17 ± 13.58/43.63 ± 10.61; PM: CAF: 71.05 ± 12.57/43.40 ± 10.00 vs. PLA: 71.17 ± 12.18/43.58 ± 9.85; squat AV-8R at 70% 1RM (m/s)/BP RtF at 70% 1RM (reps): AM: CAF: 0.56 ± 0.08 m/s/16.13 ± 4.32 vs. PLA: 0.53 ± 0.07/16.00 ± 4.68; PM: CAF: 0.54 ± 0.08/16.67 ± 3.99 vs. PLA: 0.55 ± 0.08/16.60 ± 5.12	Performance: yes, only for CMJ: ↑ (*p* = 0.035);
Romero-Moraleda et al. (16) RDB Crossover	Mean velocity/Peak velocity at 20%, 40%, 60% and 80% 1RM	13 F; trained athletes (~2 h/day; ≥5 d/week for previous 2 mo)	31 ± 6; 58.6 ± 7.8; < 100 mg/day	CAF: 3 mg/kg; PLA: cellulose; Capsule, 60 min; EF/LFP/ML; Tracker app + LH test + BBT	48 h	Mean velocity (m/s): EF: PLA: 0.72 ± 0.05/0.63 ± 0.08/0.56 ± 0.06/0.48 ± 0.07 vs. CAF: 0.69 ± 0.05/0.65 ± 0.07/0.57 ± 0.08/0.49 ± 0.07 LFP: PLA: 0.72 ± 0.09/0.61 ± 0.08/0.53 ± 0.07/0.48 ± 0.07 vs. CAF: 0.72 ± 0.09/0.65 ± 0.06/0.58 ± 0.06/0.48 ± 0.07 ML: PLA: 0.70 ± 0.10/0.63 ± 0.10/0.54 ± 0.08/0.47 ± 0.08 vs. CAF: 0.73 ± 0.10/0.66 ± 0.09/0.57 ± 0.08/0.49 ± 0.08 Peak velocity (m/s): EF: PLA: 1.33 ± 0.06/1.20 ± 0.14/1.12 ± 0.10/1.03 ± 0.09 vs. CAF: 1.24 ± 0.07/1.22 ± 0.11/1.14 ± 0.10/1.02 ± 0.09 LFP: PLA: 1.35 ± 0.19/1.21 ± 0.18/1.14 ± 0.13/1.05 ± 0.11 vs. CAF: 1.40 ± 0.18/1.26 ± 0.15/1.19 ± 0.11/1.05 ± 0.11 ML: PLA: 1.37 ± 0.18/1.25 ± 0.20/1.12 ± 0.14/1.03 ± 0.12 vs. CAF: 1.36 ± 0.18/1.25 ± 0.20/1.16 ± 0.12/1.03 ± 0.12	Performance: yes, only with mean velocity at 60% 1RM: EF/LFP: ↑ (*p* < 0.05)
Santana et al. (17) RDB Crossover	CMJ; MVIC; 1-RM half-squat; RF at 80% 1-RM	14 F; resistance-trained (5.1 ± 0.8 d/week; 1.0 ± 0.4 h/session; RT exp: 5.7 ± 3.0 y)	25.5 ± 4.0; 59.8 ± 9.0; 130.6 ± 37.0 mg/day	CAF: 5 mg/kg; PLA: cellulose; Capsule, 60 min; EF/ML; Calendar tracking + bleeding report	48 h	MVIC (N): EF/ML: PLA 97.3 ± 20.2/103.5 ± 20.2, CAF 114.1 ± 24.3/120.3 ± 24.3; 1-RM (kg): EF/ML: PLA 103.0 ± 16.0/119.6 ± 19.2, CAF 119.6 ± 19.2/127.3 ± 20.1; CMJ (cm): EF/ML: PLA 18.7 ± 2.4/21.2 ± 2.3, CAF 21.2 ± 2.3/22.7 ± 2.6; RF at 80% 1-RM (reps): EF/ML: PLA 6.0 ± 1.7/9.5 ± 2.5, CAF 10.5 ± 3.0/11.9 ± 3.1;	Performance: yes ↑ (*p* < 0.05)
Shlool et al. (65) RDB Crossover	Leg extension/hip adduction RTF at 65% 1-RM; Time under tension	11 F; strength-trained (≥1 h/day; ≥3 d/week for previous 4 y)	26.22 ± 4.27; 53.33 ± 4.26; ≤ 100 mg/day	CAF: 4 mg/kg; PLA: dextrose placebo; capsule, 60 min; EF; self-reported	48 h	Leg extension RTF(*n*): CAF: 31.7 ± 2.1 vs. PLA: 26.9 ± 1.8; Hip adduction RTF(*n*): CAF: 34.3 ± 3.5 vs. PLA: 32.8 ± 3.7	Performance: yes ↑ (*p* < 0.05)
Skinner et al. (66) RDB Crossover	Cycling time trial;	11 F; endurance-trained cyclists/triathletes (competitive ≥1 season; high volume ≥2 mo)	29.7 ± 5.3; 59.5 ± 9.7; 253.1 ± 227.8 mg/day	CAF: 3 mg/kg; PLA: placebo; Capsule, 90 min; high-hormone OCP phase; Pill mapping + ovulation test	≥5 d	Performance time (s): CAF: 3757 ± 312 vs. PLA: 3863 ± 419	Performance: yes ↓ (*p* < 0.05)
Stojanović et al. (67) RDB Crossover	CMJ without/with arm swing; SJ; Lane Agility Drill; 5-m/10-m and 20-m sprint with/without dribbling; Suicide Run	10 F; professional basketball players (8 × 90-min sessions/week; ≥1 match/week)	20.2 ± 3.9; 69.2 ± 6.3; 219 ± 141.4 mg/week (< 100 mg/day)	CAF: 3 mg/kg; PLA: dextrose 3 mg/kg; Capsule, 60 min; LP; self-report	1 week	CMJ without/with arm swing (cm): CAF: 29.20 ± 4.39/35.14 ± 5.08 vs. PLA: 27.92 ± 4.24/33.85 ± 3.92 SJ (cm): CAF: 27.22 ± 4.37 vs. PLA: 25.97 ± 3.16; Lane Agility Drill (s): CAF: 12.99 ± 0.86 vs. PLA: 13.22 ± 0.87; 5-m/10-m and 20-m sprint (s): CAF: 1.18 ± 0.11/2.01 ± 0.13/3.49 ± 0.23 vs. PLA: 1.24 ± 0.15/2.11 ± 0.18/3.59 ± 0.25; 5-m/10-m and 20-m dribbling sprint (s): CAF: 1.20 ± 0.05/2.05 ± 0.12/3.56 ± 0.25 vs. PLA: 1.22 ± 0.08/2.07 ± 0.11/3.65 ± 0.15; Suicide Run (s): CAF: 31.80 ± 1.62 vs. PLA: 32.20 ± 1.74	Performance: yes, only for 10-m and 20-m sprint without dribbling: ↓ (*p* < 0.05)
Suvi et al. (68) RDB Crossover	Treadmill walking in heat 60% VO2peak until volitional exhaustion	10 F; physically active (endurance-type recreational activities 3–4 times/week)	22.5 ± 2.0; 61.0 ± 5.4; < 60 mg/day	CAF: 6 mg/kg; PLA: wheat flour; Capsule, 4 mg/kg 60 min + 2 mg/kg immediately; HC; Not phase-controlled	1 week	Time to exhaustion (min): CAF: 82 ± 15 vs. PLA: 76 ± 11	Performance: No (*p* > 0.05)

**CAF**, caffeine; **PLA**, placebo; **CON**, control; **CA**, caffeine abstinence; **NoCA**, no caffeine abstinence; **TB**, triple-blind trial; **RDB**, randomized double-blind trial; **F**, female participants; **1-RM**, one-repetition maximum; **CMJ**, countermovement jump; **SJ**, squat jump; **MVIC**, maximal voluntary isometric contraction; **MVC**, maximal voluntary contraction; **MVICpost**, post-exercise maximal voluntary isometric contraction; **RF/RTF**, repetitions to failure; **Tlim**, time to exhaustion; **TT**, time trial; **TSAT**, taekwondo-specific agility test; **FSKT-10s**, frequency speed of kick test over 10 s; **FSKT-mult**, multiple frequency speed of kick test; **Wmax**, maximal power output; **BPT**, bench press throw; **BP**, bench press; **AV-8R**, average velocity attained during 8 repetitions; **VO2peak**, peak oxygen uptake; **LH**, luteinizing hormone; **BTT**, basal body temperature; **EF**, early follicular phase; **LFP**, late follicular phase; **PO**, peri-ovulatory phase; **LP**, luteal phase; **ML/MLP**, mid-luteal phase; **HC**, hormonal contraceptive; **OCP**, oral contraceptive pill; **AM**, morning; **PM**, afternoon; **NA**, not applicable; **cm**, centimeters; **h**, hours; **kg**, kilograms; **km**, kilometers; **m**, meters; **mg**, milligrams; **min**, minutes; **mo**, month; **Nm**, newton-meters; **s**, seconds; **W**, watts; **y**, years; **↑**, significant increase; **↓**, significant decrease.

### 3.3 Primary overall and subgroup analyses

**Figure 2:**
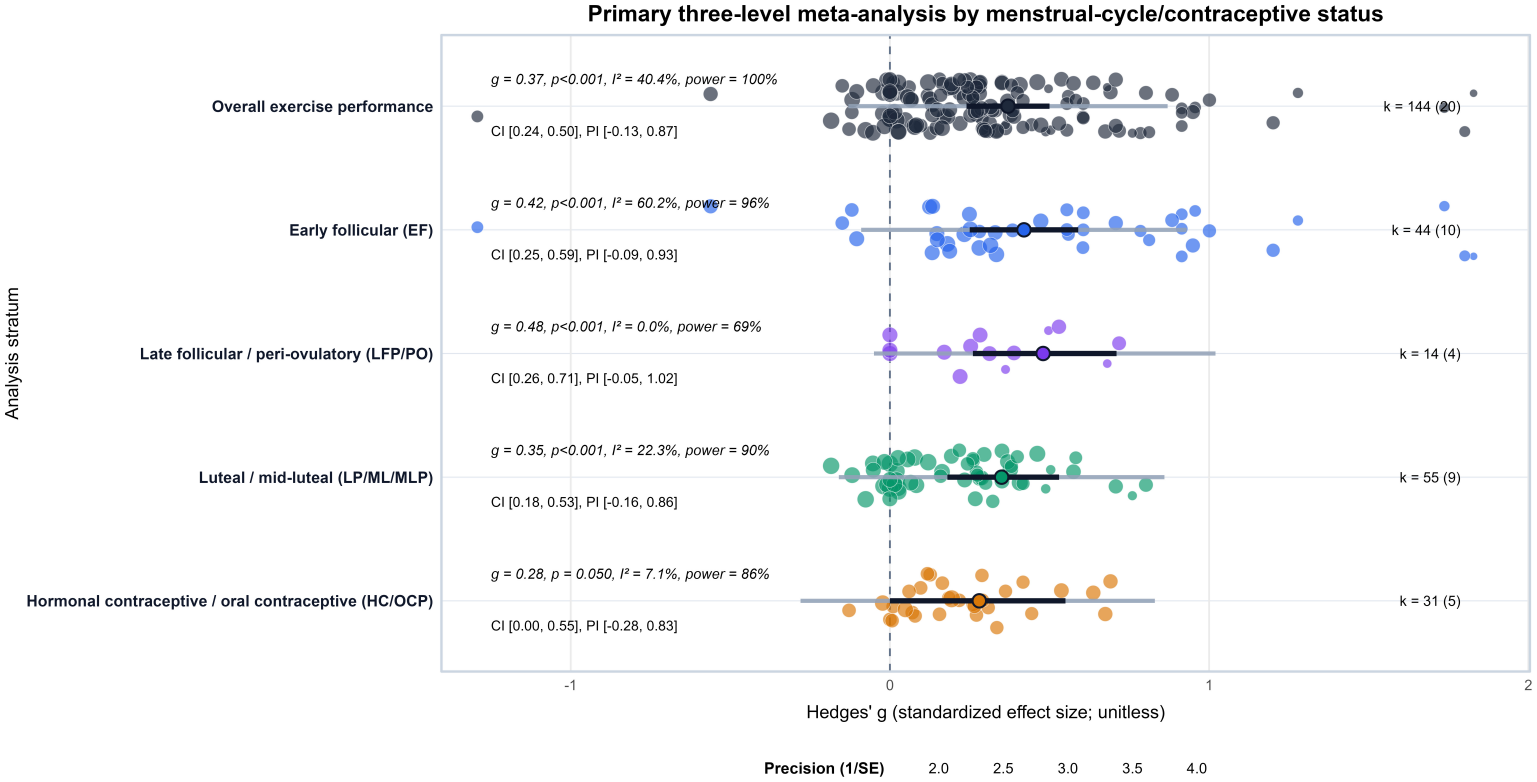
Primary three-level meta-analysis of acute caffeine ingestion by menstrual-cycle phase and hormonal contraceptive status. Dots represent individual effect sizes; dot size reflects precision (1/SE). Colored circles represent pooled Hedges' g estimates, black horizontal lines represent 95% confidence intervals, and gray horizontal lines represent 95% prediction intervals. K indicates the number of effect sizes, with the number of contributing studies shown in parentheses. Hedges' g was used as the standardized effect-size metric. CI, confidence interval; EF, early follicular; HC/OCP, hormonal contraceptive/oral contraceptive users; I^2^, inconsistency statistic; Power, statistical power for the pooled effect size; LFP/PO, late follicular/peri-ovulatory; LP/ML/MLP, luteal/mid-luteal; PI, prediction interval.

Acute caffeine ingestion was associated with a statistically significant positive effect on overall exercise performance (Hedges' g = 0.37, 95% CI: 0.24 to 0.50, *p* < 0.001; 95% PI: −0.13 to 0.87). Residual heterogeneity was moderate (I^2^ = 40.4%), and the certainty of evidence was rated as low (Figure 2; Supplementary material Appendix S3).

In the primary subgroup analysis, all menstrual-cycle and hormonal-contraceptive strata showed positive effect estimates favoring caffeine. The pooled effects were g = 0.42 (95% CI: 0.25 to 0.59, p < 0.001) in the early follicular phase, g = 0.48 (95% CI: 0.26 to 0.71, p < 0.001) in the late follicular/peri-ovulatory phase, g = 0.35 (95% CI: 0.18 to 0.53, p < 0.001) in the luteal/mid-luteal phase, and g = 0.28 (95% CI: 0.00 to 0.55, *p* = 0.050) among hormonal contraceptive/oral contraceptive users. However, the between-subgroup omnibus test was not significant (F(3,140) = 0.70, *p* = 0.551), indicating no clear evidence that menstrual-cycle phase or hormonal contraceptive status moderated the ergogenic effect of acute caffeine ingestion (Figure 2; Supplementary material Appendix S3).

### 3.4 Subgroup analysis by specific menstrual phase

**Figure 3:**
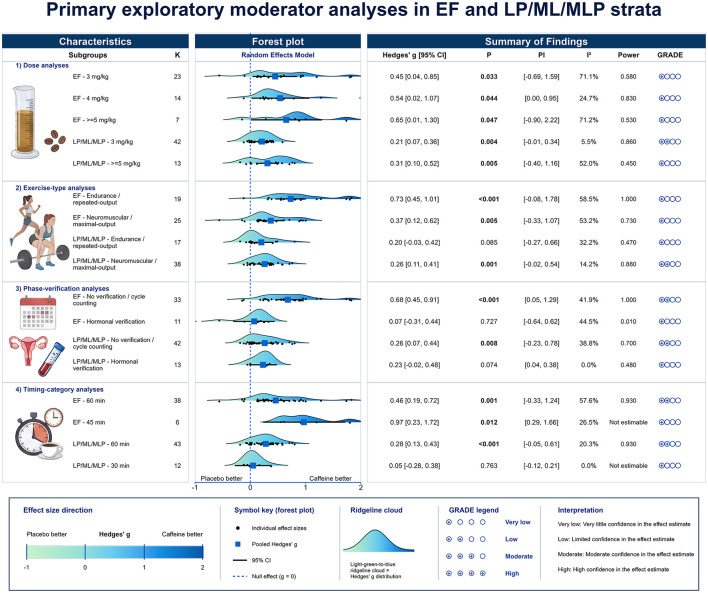
Exploratory within-stage moderator analyses in the early follicular and luteal/mid-luteal strata. **K**, the total number of effect sizes included in each pooled estimate; **Hedges' g**, the standardized effect-size metric used for pooled effects; **95% CI**, 95% confidence interval; **95% PI**, 95% prediction interval; ***P*-value**, statistical significance of the pooled effect within each category; **I**^**2**^, quantitative indicator of heterogeneity; **Power**, statistical power for the pooled effect size; **GRADE**, Grading of Recommendations Assessment, Development and Evaluation system for evaluating certainty of evidence. Black dots represent individual effect sizes; blue squares represent pooled Hedges' g estimates; black horizontal lines represent 95% CIs; ridgeline clouds represent the distribution of Hedges' g values; dashed vertical line represents the null effect. **EF**, early follicular; **LP/ML/MLP**, luteal/mid-luteal.

Exploratory within-stage moderator analyses were conducted primarily in the early follicular (EF) and luteal/mid-luteal (LP/ML/MLP) strata, where sufficient data were available. In dose-specific analyses, positive effects were observed across all EF dose categories: 3 mg/kg (g = 0.45, 95% CI: 0.04 to 0.85), 4 mg/kg (g = 0.54, 95% CI: 0.02 to 1.07), and ≥5 mg/kg (g = 0.65, 95% CI: 0.01 to 1.30). In the LP/ML/MLP stratum, both 3 mg/kg (g = 0.21, 95% CI: 0.07 to 0.36) and ≥5 mg/kg (g = 0.31, 95% CI: 0.10 to 0.52) showed positive effects. However, there was no evidence of dose-dependent moderation within either EF (F(2, 41) = 0.16, p = 0.854) or LP/ML/MLP (F(1, 53) = 0.73, *p* = 0.398) (Figure 3; Supplementary material Appendix S4).

For exercise type, a significant within-stage difference was observed in the EF stratum. Endurance/repeated-output outcomes showed a larger effect (g = 0.73, 95% CI: 0.45 to 1.01) than neuromuscular/maximal-output outcomes (g = 0.37, 95% CI: 0.12 to 0.62; *p* between = 0.013). In contrast, exercise type did not significantly moderate caffeine effects in the LP/ML/MLP stratum (p between = 0.602), although neuromuscular/maximal-output outcomes showed a significant positive effect (g = 0.26, 95% CI: 0.11 to 0.41).

Phase-verification method also moderated the EF results. Studies using no verification/cycle counting showed a larger pooled effect (g = 0.68, 95% CI: 0.45 to 0.91) than studies using hormonal verification (g = 0.07, 95% CI: −0.31 to 0.44; *p* between = 0.008). This pattern was not observed in the LP/ML/MLP stratum (p between = 0.871). Timing-category analyses showed positive effects for the predominant 60-min ingestion protocol in both EF (g = 0.46, 95% CI: 0.19 to 0.72) and LP/ML/MLP (g = 0.28, 95% CI: 0.13 to 0.43), but timing did not significantly moderate effects within either stratum. Additional analyses for the late follicular/peri-ovulatory and hormonal contraceptive/oral contraceptive strata were considered exploratory because of sparse data and are reported in Supplementary material Appendix S5.

### 3.5 Risk of bias and quality of methods

Across included exercise-performance trials, no study was rated as overall “low risk” using RoB 2; one study was judged “high risk” (46), and the remaining studies were classified as having “some concerns.” Domain-level judgments were generally favorable for missing outcome data and outcome measurement, whereas concerns were more frequent for randomization procedures, period/carryover effects, deviations from intended interventions, and selection of the reported result. Overall, the evidence base showed a predominantly moderate risk-of-bias profile, with the high-risk judgment mainly driven by selective-reporting concerns in one trial (Supplementary material Appendix S8).

Egger's regression tests were conducted for 27 analyses with at least 10 effect-size estimates, comprising the primary overall model and 26 eligible subgroup or exploratory analyses. Seventeen analyses showed significant funnel-plot asymmetry (*p* < 0.05), including the overall model, EF, LP/ML/MLP, and several nested exploratory cells; 10 analyses were non-significant (*p* > 0.05), including the LFP/PO and HC/OCP overall strata. Given that several tests involved few independent studies despite having ≥10 effect sizes, these results were interpreted as evidence of possible small-study effects rather than definitive publication bias (Supplementary material Appendix S9). Sunset funnel plots further indicated variable and often limited statistical power in several subgroup cells (Supplementary material Appendix S10).

For exercise-performance outcomes, the mean modified PEDro score was 7.9, consistent with good methodological quality. More information is provided in Supplementary material Appendix S11. In addition, the certainty of evidence for each primary, subgroup, and exploratory outcome was evaluated using the GRADE approach, with details provided in (Supplementary material Appendix S12).

### 3.6 Sensitivity analysis

#### 3.6.1 Sensitivity analysis for primary effect

**Figure 4:**
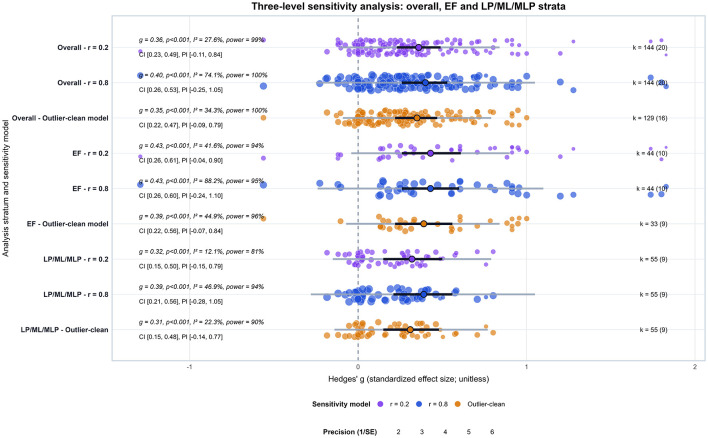
Sensitivity analyses for the primary three-level meta-analysis under alternative correlation assumptions and outlier-clean models. Dots represent individual effect sizes; dot size reflects precision (1/SE). Colored circles represent pooled Hedges' g estimates, black horizontal lines represent 95% confidence intervals, and gray horizontal lines represent 95% prediction intervals. Sensitivity models include alternative within-participant correlation assumptions (**r** = **0.2** and **r** = **0.8**) and outlier-clean analyses. **K** indicates the number of effect sizes, with the number of contributing studies shown in parentheses. **CI**, confidence interval; **EF**, early follicular; **I**^**2**^, inconsistency statistic; **Power**, statistical power for the pooled effect size; **LP/ML/MLP**, luteal/mid-luteal; **PI**, prediction interval.

Sensitivity analyses using alternative assumed within-participant correlations, outlier-clean models, and leave-one-out procedures supported the robustness of the primary overall effect (Figure 4; Supplementary material Appendix S3 and S6). The pooled effect remained positive and statistically significant across sensitivity models, and the menstrual-cycle/contraceptive omnibus test remained non-significant. Leave-one-out analyses did not materially alter the overall interpretation; however, the LFP/PO estimate became borderline non-significant after removal of Romero-Moraleda et al. (16), indicating limited stability in this sparse stratum (Supplementary material Appendix S7).

#### 3.6.2 Sensitivity analysis for exploratory moderator effects

In the EF stratum, the exercise-type contrast remained significant after outlier exclusion, whereas the phase-verification contrast was attenuated to a borderline level. In the LP/ML/MLP stratum, positive effects persisted for 3 mg/kg dosing, neuromuscular/maximal-output performance, and 60-min ingestion, although estimates were generally smaller after outlier exclusion. Findings for LFP/PO and HC/OCP were less stable and were interpreted descriptively because of limited study counts, sparse cells, or non-estimable models. Overall, the exploratory moderator patterns showed partial robustness, but these findings should be considered hypothesis-generating rather than confirmatory (Supplementary material Appendix S4 and S5).

## 4 Discussion

### 4.1 Principal findings and scientific contribution

The principal finding of this review is not merely that acute caffeine ingestion improves exercise performance in women, but that its ergogenic effect appears to be maintained across distinct reproductive-hormonal environments, including low-oestrogen/low-progesterone, high-oestrogen, progesterone-exposed luteal, and exogenous-hormone contexts. This pattern argues against a simple model in which caffeine is effective only in a specific menstrual phase or ineffective under hormonal-contraceptive use, and broadly aligns with previous meta-analytic evidence showing limited average effects of menstrual-cycle phase and oral-contraceptive use on exercise performance (5, 6). Instead, it suggests that caffeine's primary ergogenic mechanisms, including adenosine-receptor antagonism, enhanced central drive, reduced perceived exertion, improved neuromuscular function, and greater fatigue tolerance, may remain relevant across different ovarian and exogenous hormonal backgrounds (47–50).

The scientific value of this review lies in showing that caffeine's effects on women's exercise performance cannot be interpreted from menstrual-phase labels alone. Oestradiol and progesterone may influence substrate use, thermoregulation, ventilation, neuromuscular function, symptom burden, and perceived fatigue (5, 51), whereas hormonal contraceptives may alter endogenous hormone profiles, caffeine clearance, and tolerability (4, 6, 52, 53). Therefore, the key issue is not simply whether caffeine works in women, but whether its effects are being measured in accurately defined endocrine states and in exercise tasks that are physiologically sensitive to caffeine's mechanisms. By integrating menstrual-cycle phase, hormonal-contraceptive status, exercise-task phenotype, and phase-verification rigor, this review provides a more biologically grounded framework for interpreting caffeine supplementation in women (4, 36, 54).

### 4.2 Menstrual-cycle phase and hormonal contraceptive status

The positive estimates observed across early follicular, late follicular/peri-ovulatory, luteal/mid-luteal, and hormonal-contraceptive/oral-contraceptive strata suggest that caffeine's ergogenic potential is not confined to a single reproductive-hormonal condition. However, the non-significant omnibus test indicates that current evidence is insufficient to define a single “best” menstrual phase or contraceptive state for caffeine use.

These findings should not be interpreted as evidence that ovarian hormones are irrelevant. In the early follicular phase, caffeine effects occur under relatively low oestradiol and progesterone; in the late follicular/peri-ovulatory phase, they occur under higher oestradiol and low progesterone; and in the luteal/mid-luteal phase, they occur under greater progesterone exposure. These hormonal environments may influence thermoregulation, ventilation, substrate use, fluid balance, symptom burden, perceived fatigue, and exercise performance (5, 51). The persistence of positive estimates across these environments therefore suggests that hormonal status may shape the expression, rather than the presence or absence, of caffeine's ergogenic effect.

Hormonal-contraceptive users require separate caution because this category does not represent a single endocrine state. Formulation, oestrogen dose, progestin type, androgenic profile, active-pill phase, and hormone-free interval may all influence physiological responses (4, 5). In addition, oestrogen-containing contraceptives may slow CYP1A2-mediated caffeine clearance and alter caffeine exposure, tolerability, and timing of peak effect (52, 53). Because most studies did not measure circulating hormones, caffeine pharmacokinetics, genotype, or symptom responses, current evidence does not support rigid phase-specific or contraceptive-specific caffeine prescriptions. Future interpretation should therefore prioritize accurately defined endocrine states and rigorous menstrual or contraceptive-cycle verification (4, 36, 54).

### 4.3 Exercise task type as an effect-shaping factor

One of the most informative exploratory signals was the stage-specific task-type pattern. In the early follicular phase, caffeine showed a larger effect for endurance/repeated-output outcomes than for neuromuscular/maximal-output outcomes, whereas no comparable task-type moderation was observed in the luteal/mid-luteal phase. This pattern suggests that caffeine's ergogenic expression may depend not only on reproductive-hormonal context, but also on whether the exercise task is constrained by sustained output, accumulated fatigue, and perceptual tolerance.

Physiologically, the early follicular phase represents a relatively low-oestrogen and low-progesterone environment. Within this context, endurance and repeated-output tasks may provide greater opportunity for caffeine's central and perceptual mechanisms to translate into measurable performance benefits, including enhanced central drive, reduced perceived exertion, improved pacing, greater pain tolerance, and fatigue resistance (48–50). Brief maximal-output tasks may still benefit from caffeine, particularly through effects on motor-unit recruitment and voluntary activation, but their dependence on rapid force production, technical execution, training status, and measurement reliability may make the observable effect smaller or more protocol-dependent (1, 47).

The absence of a similar contrast in the luteal/mid-luteal phase may reflect the more complex post-ovulatory hormonal milieu, characterized by greater progesterone exposure together with moderate-to-high oestradiol. In this context, thermoregulation, ventilation, substrate use, fluid balance, symptom burden, and perceived fatigue may alter the constraints limiting performance, particularly during sustained or repeated-output exercise (5, 51). Comparable inference was not possible for the late follicular/peri-ovulatory and hormonal-contraceptive/oral-contraceptive strata because these analyses were sparse and reported descriptively in the Supplementary material. Therefore, these findings should be interpreted as hypothesis-generating, and future trials should prespecify exercise phenotypes within hormonally verified reproductive contexts rather than treating all performance outcomes as interchangeable expressions of a single caffeine effect (4, 36).

### 4.4 Phase verification as a methodological moderator

Phase verification determines whether a menstrual-phase label represents the intended endocrine state. This was most evident in the early follicular stratum, where studies using no verification or cycle counting showed larger caffeine effects than hormonally verified studies. This pattern should not be interpreted as evidence that non-verified studies are more valid, or that caffeine is ineffective when the early follicular phase is hormonally confirmed. Rather, it suggests that nominal early follicular labels may not consistently capture the intended low-oestrogen/low-progesterone environment, allowing endocrine misclassification to distort the apparent magnitude of caffeine's ergogenic effect (4, 36).

The absence of a comparable verification-related pattern in the luteal/mid-luteal stratum does not imply that luteal verification is unnecessary. The post-ovulatory milieu involves greater progesterone exposure together with moderate-to-high oestradiol, and phase timing alone may not capture the physiological variability relevant to caffeine responses, including symptom burden, perceived fatigue, thermoregulation, substrate use, and tolerability (5, 51). Thus, verification is necessary for biological interpretation, but direct endocrine, pharmacokinetic, and mechanistic phenotyping remains essential.

Future trials should define reproductive-hormonal targets *a priori* and match verification procedures to the research question. Naturally menstruating women require prospective cycle tracking, urinary LH testing when ovulation timing is relevant, and serum or salivary oestradiol and progesterone confirmation (36, 54). Hormonal-contraceptive studies should report formulation, progestin type, dose, active-pill phase, and withdrawal-bleeding phase (4). Without these procedures, phase-specific caffeine responses should remain cautiously interpreted.

### 4.5 Dose, timing, and habitual caffeine intake

Dose-specific findings support practical caution rather than a phase-specific dose-response model. The available evidence provides no indication that higher caffeine doses are required in low-oestrogen/low-progesterone or progesterone-exposed luteal environments. A moderate, well-tolerated dose remains the most defensible starting point, consistent with sport-nutrition guidance that caffeine is commonly effective within the 3–6 mg/kg range, while higher doses may increase side effects without ensuring greater performance benefit (37, 50, 55).

Timing and habitual caffeine intake should be interpreted similarly. The predominance of 60-min pre-exercise ingestion supports this as a practical default for many conventional caffeine protocols, but current evidence does not justify menstrual-phase-specific timing rules; timing should also consider caffeine formulation and individual pharmacokinetic variability (37, 50). Likewise, uneven habitual-intake data and sparse high-intake evidence do not support routine caffeine withdrawal or automatic dose escalation in habitual users (56). Overall, these findings support a minimum-effective-dose approach aligned with caffeine formulation, exercise demands, individual tolerability, and reproductive-hormonal context, rather than rigid phase-specific dosing, timing, or withdrawal prescriptions.

### 4.6 Strengths and limitations

This review provides a female-specific synthesis of acute caffeine ingestion and exercise performance within a reproductive-hormonal framework, rather than extrapolating from male-dominant or mixed-sex evidence. Key strengths include the use of three-level meta-analysis to account for dependent effect sizes from crossover and multi-outcome trials (28), a structured moderator framework integrating menstrual-cycle phase, hormonal-contraceptive status, exercise-task phenotype, dose, timing, habitual intake, and phase verification, and a clear distinction between primary and exploratory inferences. Sensitivity analyses, certainty assessment, risk-of-bias evaluation, and small-study-effect assessment further strengthened interpretation by considering robustness, certainty, and credibility rather than statistical significance alone.

Several limitations temper interpretation. Although the main ergogenic signal was stable across sensitivity analyses, certainty of evidence remained limited, and prediction intervals indicate that the average effect should not be assumed for every individual or context (33, 44). Evidence was sparse in some reproductive strata, particularly late follicular/peri-ovulatory and hormonal-contraceptive/oral-contraceptive groups, and uneven moderator distributions limited separation of menstrual phase, contraceptive status, exercise phenotype, dose, timing, verification method, and study quality. In addition, inconsistent phase verification, broad grouping of hormonal-contraceptive users, and limited measurement of circulating hormones, caffeine pharmacokinetics, genotype, adverse effects, sleep, symptom burden, and participant-level habitual intake constrained individualized, phase-specific, contraceptive-specific, task-specific, and mechanistic inference. A further limitation concerns exercise-task classification: although outcomes were grouped into two broad categories according to the characteristics of the included studies and to preserve model stability, this approach inevitably introduced physiological heterogeneity. The neuromuscular/maximal-output category included maximal strength, jumping, sprint/agility, movement velocity, maximal voluntary contraction, and Wingate-derived power outcomes, which differ in duration, energy-system contribution, motor-unit recruitment, fatigue development, and lactate accumulation. Therefore, although this grouping provided a practical framework for exploratory moderator analysis, pooled task-type estimates may have masked or diluted mechanism-specific ergogenic pathways of caffeine and should be interpreted as broad phenotype-level signals rather than mechanism-specific effects.

### 4.7 Practical implications and future directions

Practically, acute caffeine ingestion should be applied as a context-sensitive strategy rather than prescribed solely according to menstrual-cycle phase. Current evidence does not support avoiding caffeine in any specific phase, assuming that hormonal-contraceptive users are uniformly less responsive, or applying rigid phase-specific dosing and timing rules. A moderate, well-tolerated dose, commonly around 3 mg/kg approximately 60 min before exercise, is a reasonable starting point, with subsequent adjustment according to exercise demands, perceived benefit, side effects, sleep, recovery, habitual intake, and reproductive-hormonal context. Therefore, caffeine use in women should be individualized according to task demands and tolerability, while menstrual-cycle phase and hormonal-contraceptive status should be considered as contextual factors rather than stand-alone prescription rules.

## 5 Conclusion

Acute caffeine ingestion produced a small average ergogenic effect on exercise performance in women. Exploratory findings suggest that this effect may vary by exercise-task characteristics within different reproductive-hormonal contexts, rather than by menstrual-cycle phase or hormonal-contraceptive status alone. Current evidence supports a context-sensitive interpretation rather than rigid phase- or contraceptive-specific recommendations; however, given the low-to-very-low certainty of evidence, these observations should be regarded as hypothesis-generating. Future trials should integrate rigorous hormonal verification, contraceptive profiling, prespecified exercise phenotypes, and mechanistic assessment to refine female-specific caffeine guidance.

## Data Availability

The data and analysis code supporting the findings of this study are publicly available in the Open Science Framework (OSF) project at https://osf.io/5y69g/.
