# Supplementary material for: Acute caffeine ingestion and exercise performance in women: a systematic review and meta-analysis of menstrual-cycle phase and hormonal contraceptive status
Source: Front Nutr. 2026 Jul 10;13:1876198. doi: 10.3389/fnut.2026.1876198 (PMC13395651; doi:10.3389/fnut.2026.1876198)
Supplement: Supplementary file 1 [file Supplementary_file_1.docx]

**Electronic Supplementary Material Contents**

| **Number** | **Material** | **Page** |
| --- | --- | --- |
| 1 | Electronic Supplementary Material Appendix S1 (PRISMA 2020 checklist) | P2-4 |
| 2 | Electronic Supplementary Material Appendix S2 (Table. Summary of effect size calculation procedures) | P5 |
| 3 | Electronic Supplementary Material Appendix S3 (Table. Main and sensitivity meta-analytic results) | P6 |
| 4 | Electronic Supplementary Material Appendix S4 (Table. Exploratory within-stage moderator analyses for selected strata) | P7-8 |
| 5 | Electronic Supplementary Material Appendix S5 (Table. Sparse-strata exploratory within-stage moderator analyses for LFP/PO and HC/OCP strata). | P9-11 |
| 6 | Electronic Supplementary Material Appendix S6 (Leave-one-out sensitivity analysis for the overall model) | P12 |
| 7 | Electronic Supplementary Material S7 (Leave-one-out sensitivity analyses for menstrual-cycle and hormonal-contraceptive subgroups) | P13 |
| 8 | Electronic Supplementary Material Appendix S8 (RoB 2 Assessment of Risk of Bias in the Included Studies) | P14 |
| 9 | Electronic Supplementary Material Appendix S9 (Contour-enhanced funnel plots for primary, subgroup, and exploratory analyses) | P15-16 |
| 10 | Electronic Supplementary Material Appendix S10 (Sunset funnel plots for statistical power visualization in primary, subgroup, and exploratory analyses) | P17-18 |
| 11 | Electronic Supplementary Material Appendix S11 (Modified PEDro assessment of the included randomized controlled trials) | P19 |
| 12 | Electronic Supplementary Material Appendix S12 (Table. GRADE certainty assessment for overall, subgroup, and exploratory meta-analytic outcomes) | P20-22 |

**Electronic Supplementary Material Appendix S1 (PRISMA 2020 checklist)**

| **Section and Topic** | **Item #** | **Checklist item** | **Reported location** |
| --- | --- | --- | --- |
| **TITLE : Acute Caffeine Ingestion and Exercise Performance in Women: A Systematic Review and Meta-Analysis of Menstrual-Cycle Phase and Hormonal Contraceptive Status** | | |  |
| Title | 1 | Identify the report as a systematic review. | Title page |
| **ABSTRACT** | | |  |
| Abstract | 2 | See the PRISMA 2020 for Abstracts checklist. | Abstract |
| **INTRODUCTION** | | |  |
| Rationale | 3 | Describe the rationale for the review in the context of existing knowledge. | Introduction |
| Objectives | 4 | Provide an explicit statement of the objective(s) or question(s) the review addresses. | Introduction |
| **METHODS** | | |  |
| Eligibility criteria | 5 | Specify the inclusion and exclusion criteria for the review and how studies were grouped for the syntheses. | Methods—Eligibility |
| Information sources | 6 | Specify all databases, registers, websites, organisations, reference lists and other sources searched or consulted to identify studies. Specify the date when each source was last searched or consulted. | Methods—Search |
| Search strategy | 7 | Present the full search strategies for all databases, registers and websites, including any filters and limits used. | Methods—Search |
| Selection process | 8 | Specify the methods used to decide whether a study met the inclusion criteria of the review, including how many reviewers screened each record and each report retrieved, whether they worked independently, and if applicable, details of automation tools used in the process. | Methods—Screening |
| Data collection process | 9 | Specify the methods used to collect data from reports, including how many reviewers collected data from each report, whether they worked independently, any processes for obtaining or confirming data from study investigators, and if applicable, details of automation tools used in the process. | Methods—Extraction |
| Data items | 10a | List and define all outcomes for which data were sought. Specify whether all results that were compatible with each outcome domain in each study were sought (e.g. for all measures, time points, analyses), and if not, the methods used to decide which results to collect. | Methods—Eligibility/Extraction |
|  | 10b | List and define all other variables for which data were sought (e.g. participant and intervention characteristics, funding sources). Describe any assumptions made about any missing or unclear information. | Methods—Extraction/Moderators |
| Study risk of bias assessment | 11 | Specify the methods used to assess risk of bias in the included studies, including details of the tool(s) used, how many reviewers assessed each study and whether they worked independently, and if applicable, details of automation tools used in the process. | Methods—RoB assessment |
| Effect measures | 12 | Specify for each outcome the effect measure(s) (e.g. risk ratio, mean difference) used in the synthesis or presentation of results. | Methods—Effect sizes |
| Synthesis methods | 13a | Describe the processes used to decide which studies were eligible for each synthesis (e.g. tabulating the study intervention characteristics and comparing against the planned groups for each synthesis (item #5)). | Methods—Eligibility/Moderators |
|  | 13b | Describe any methods required to prepare the data for presentation or synthesis, such as handling of missing summary statistics, or data conversions. | Methods—Extraction/Effect sizes; App. S2 |
|  | 13c | Describe any methods used to tabulate or visually display results of individual studies and syntheses. | Results; Figs. 1–4; Apps. S3–S10 |
|  | 13d | Describe any methods used to synthesize results and provide a rationale for the choice(s). If meta-analysis was performed, describe the model(s), method(s) to identify the presence and extent of statistical heterogeneity, and software package(s) used. | Methods—Meta-analysis |
|  | 13e | Describe any methods used to explore possible causes of heterogeneity among study results (e.g. subgroup analysis, meta-regression). | Methods—Moderators |
|  | 13f | Describe any sensitivity analyses conducted to assess robustness of the synthesized results. | Methods—Sensitivity; Fig. 4; Apps. S3, S6–S7 |
| Reporting bias assessment | 14 | Describe any methods used to assess risk of bias due to missing results in a synthesis (arising from reporting biases). | Methods—Reporting bias; Apps. S9–S10 |
| Certainty assessment | 15 | Describe any methods used to assess certainty (or confidence) in the body of evidence for an outcome. | Methods—GRADE; App. S12 |
| **RESULTS** | | |  |
| Study selection | 16a | Describe the results of the search and selection process, from the number of records identified in the search to the number of studies included in the review, ideally using a flow diagram. | Results—Selection; Fig. 1 |
|  | 16b | Cite studies that might appear to meet the inclusion criteria, but which were excluded, and explain why they were excluded. | Results—Selection; Fig. 1 |
| Study characteristics | 17 | Cite each included study and present its characteristics. | Results—Characteristics; Table 1 |
| Risk of bias in studies | 18 | Present assessments of risk of bias for each included study. | Results—RoB; Apps. S8, S11 |
| Results of individual studies | 19 | For all outcomes, present, for each study: (a) summary statistics for each group (where appropriate) and (b) an effect estimate and its precision (e.g. confidence/credible interval), ideally using structured tables or plots. | Results; Figs. 2–4; Apps. S3–S7 |
| Results of syntheses | 20a | For each synthesis, briefly summarise the characteristics and risk of bias among contributing studies. | Results—Characteristics/RoB; Table 1; Apps. S8, S11 |
|  | 20b | Present results of all statistical syntheses conducted. If meta-analysis was done, present for each the summary estimate and its precision (e.g. confidence/credible interval) and measures of statistical heterogeneity. If comparing groups, describe the direction of the effect. | Results—Syntheses; Figs. 2–4; Apps. S3–S5 |
|  | 20c | Present results of all investigations of possible causes of heterogeneity among study results. | Results—Moderators; Fig. 3; Apps. S4–S5 |
|  | 20d | Present results of all sensitivity analyses conducted to assess the robustness of the synthesized results. | Results—Sensitivity; Fig. 4; Apps. S3, S6–S7 |
| Reporting biases | 21 | Present assessments of risk of bias due to missing results (arising from reporting biases) for each synthesis assessed. | Results—Reporting bias; Apps. S9–S10 |
| Certainty of evidence | 22 | Present assessments of certainty (or confidence) in the body of evidence for each outcome assessed. | Results—GRADE; App. S12 |
| **DISCUSSION** | | |  |
| Discussion | 23a | Provide a general interpretation of the results in the context of other evidence. | Discussion |
|  | 23b | Discuss any limitations of the evidence included in the review. | Discussion—Limitations |
|  | 23c | Discuss any limitations of the review processes used. | Discussion—Limitations |
|  | 23d | Discuss implications of the results for practice, policy, and future research. | Discussion/Conclusions |
| **OTHER INFORMATION** | | |  |
| Registration and protocol | 24a | Provide registration information for the review, including register name and registration number, or state that the review was not registered. | Methods—Registration |
|  | 24b | Indicate where the review protocol can be accessed, or state that a protocol was not prepared. | Methods—Registration; OSF |
|  | 24c | Describe and explain any amendments to information provided at registration or in the protocol. | N/A |
| Support | 25 | Describe sources of financial or non-financial support for the review, and the role of the funders or sponsors in the review. | Funding statement |
| Competing interests | 26 | Declare any competing interests of review authors. | Conflict of interest |
| Availability of data, code and other materials | 27 | Report which of the following are publicly available and where they can be found: template data collection forms; data extracted from included studies; data used for all analyses; analytic code; any other materials used in the review. | Data availability; OSF; Supplementary material |

**Electronic Supplementary Material Appendix S2 (Table. Summary of effect size calculation procedures)**

| Step | Data condition | Calculation Formula | Description |
| --- | --- | --- | --- |
| ① | Pre–post data available | $\Delta CAF= M_{\mathrm{CAFpost}}- M_{\mathrm{CAFpre}}$  $\Delta PLA= M_{\mathrm{PLApost}}- M_{\mathrm{PLApre}}$  $\mathrm{MD}= \Delta CAF- \Delta PLA$ | Calculate the mean change within each condition (acute caffeine ingestion and placebo) and use their difference (MD) as the comparison metric. |
| ② | Only post-intervention data available | $\mathrm{MD}= M_{\mathrm{CAF}}- M_{\mathrm{PLA}}$ | When only post values are reported, the mean difference between conditions is used directly. |
| ③ | SD of change (for pre–post data) | $\begin{aligned} \mathrm{SD}_{\mathrm{change}}=\sqrt{{\mathrm{SD}^{2}}_{\mathrm{pre}}+{\mathrm{SD}^{2}}_{\mathrm{post}}-\left（ 2\times r\times\mathrm{SD}_{\mathrm{pre}}\times\mathrm{SD}_{\mathrm{post}} \right）} \end{aligned}$ | Computes the within-subject SD of change using the correlation (r) between pre- and post-measurements. |
| ④ | Pooled SD (for crossover design) | $\mathrm{SD}_{\mathrm{pooled}}=\sqrt{\frac{{\mathrm{SD}^{2}}_{\mathrm{CAF}}+{\mathrm{SD}^{2}}_{\mathrm{PLA}}}{2}}$ | Estimates the pooled variability across both conditions. |
| ⑤ | Effect size (Hedges’ *g*) | $Hedge’s g= \frac{M_{\mathrm{CAF}}- M_{\mathrm{PLA}}}{\mathrm{SD}_{\mathrm{pooled}}}\times\left( 1-\frac{3}{4\left( N-1 \right)-1} \right)$ | Standardized mean difference corrected for small-sample bias. |
| ⑥ | Standard error (SE) of *g* | $SE=\sqrt{\frac{1}{N}+\frac{g^{2}}{2N}}\times\sqrt{2\left( 1-r \right)}$ | Adjusts SE to account for within-subject correlation between acute caffeine ingestion and placebo conditions. |
| ⑦ | Assumed correlation (*r*) | *r* = 0.50 (primary analysis)  *r* = 0.20 and 0.80 (sensitivity) | The same r value was used for both pre–post and between-condition comparisons to ensure consistency and comparability. |

**Electronic Supplementary Material Appendix S3 (Table. Main and sensitivity meta-analytic results)**

| **Section** | **Analysis** | **Studies (n)** | **Effect sizes (n)** | **Hedges' g (95% CI)** | **P within** | **P between** | **I² (%)** | **PI** | **Power** |
| --- | --- | --- | --- | --- | --- | --- | --- | --- | --- |
| **Primary overall model** | **Overall three-level model** | 20 | 144 | **0.37 (0.24 to 0.50)** | <0.001 |  | 40.4 | -0.13 to 0.87 | 1.000 |
| **Primary overall model** | **Between-subgroup omnibus test** | 20 | 144 | F(3,140) = 0.70 |  | 0.551 | Not applicable |  |  |
| **Primary overall model** | Early follicular (EF) | 10 | 44 | 0.42 (0.25 to 0.59) | <0.001 | Ref | 60.2 | -0.09 to 0.93 | 0.960 |
| **Primary overall model** | Late follicular / peri-ovulatory (LFP/PO) | 4 | 14 | 0.48 (0.26 to 0.71) | <0.001 | 0.561 | 0.0 | -0.05 to 1.02 | 0.690 |
| **Primary overall model** | Luteal / mid-luteal (LP/ML/MLP) | 9 | 55 | 0.35 (0.18 to 0.53) | <0.001 | 0.470 | 22.3 | -0.16 to 0.86 | 0.900 |
| **Primary overall model** | Hormonal contraceptive / oral contraceptive (HC/OCP) | 5 | 31 | 0.28 (0.00 to 0.55) | 0.050 | 0.382 | 7.1 | -0.28 to 0.83 | 0.860 |
| **Outlier sensitivity (after outliers)** | **Overall three-level model after excluding outliers** | 16 | 129 | **0.35 (0.22 to 0.47)** | <0.001 |  | 34.3 | -0.09 to 0.79 | 1.000 |
| **Outlier sensitivity (after outliers)** | **Between-subgroup omnibus test after excluding outliers** | 16 | 129 | F(3,125) = 0.40 |  | 0.753 | Not applicable |  |  |
| **Outlier sensitivity (after outliers)** | Early follicular (EF) | 9 | 33 | 0.39 (0.22 to 0.56) | <0.001 | Ref | 44.9 | -0.07 to 0.84 | 0.960 |
| **Outlier sensitivity (after outliers)** | Late follicular / peri-ovulatory (LFP/PO) | 4 | 14 | 0.42 (0.20 to 0.64) | <0.001 | 0.779 | 0.0 | -0.06 to 0.89 | 0.690 |
| **Outlier sensitivity (after outliers)** | Luteal / mid-luteal (LP/ML/MLP) | 9 | 55 | 0.31 (0.15 to 0.48) | <0.001 | 0.428 | 22.3 | -0.14 to 0.77 | 0.900 |
| **Outlier sensitivity (after outliers)** | Hormonal contraceptive / oral contraceptive (HC/OCP) | 2 | 27 | 0.31 (-0.01 to 0.64) | 0.061 | 0.699 | 31.3 | -0.22 to 0.85 | 0.520 |
| **Sensitivity analysis (r = 0.2)** | **Overall three-level model** | 20 | 144 | **0.36 (0.23 to 0.49)** | <0.001 |  | 27.6 | -0.11 to 0.84 | 0.990 |
| **Sensitivity analysis (r = 0.2)** | **Between-subgroup omnibus test** | 20 | 144 | F(3,140) = 0.72 |  | 0.543 | Not applicable |  |  |
| **Sensitivity analysis (r = 0.2)** | Early follicular (EF) | 10 | 44 | 0.43 (0.26 to 0.61) | <0.001 | Ref | 41.6 | -0.04 to 0.90 | 0.940 |
| **Sensitivity analysis (r = 0.2)** | Late follicular / peri-ovulatory (LFP/PO) | 4 | 14 | 0.45 (0.20 to 0.71) | <0.001 | 0.885 | 0.0 | -0.05 to 0.96 | 0.610 |
| **Sensitivity analysis (r = 0.2)** | Luteal / mid-luteal (LP/ML/MLP) | 9 | 55 | 0.32 (0.15 to 0.50) | <0.001 | 0.297 | 12.1 | -0.15 to 0.79 | 0.810 |
| **Sensitivity analysis (r = 0.2)** | Hormonal contraceptive / oral contraceptive (HC/OCP) | 5 | 31 | 0.26 (-0.02 to 0.54) | 0.071 | 0.305 | 5.0 | -0.26 to 0.78 | 0.630 |
| **Sensitivity analysis (r = 0.8)** | **Overall three-level model** | 20 | 144 | **0.40 (0.26 to 0.53)** | <0.001 |  | 74.1 | -0.25 to 1.05 | 1.000 |
| **Sensitivity analysis (r = 0.8)** | **Between-subgroup omnibus test** | 20 | 144 | F(3,140) = 0.86 |  | 0.462 | Not applicable |  |  |
| **Sensitivity analysis (r = 0.8)** | Early follicular (EF) | 10 | 44 | 0.43 (0.26 to 0.60) | <0.001 | Ref | 88.2 | -0.24 to 1.10 | 0.950 |
| **Sensitivity analysis (r = 0.8)** | Late follicular / peri-ovulatory (LFP/PO) | 4 | 14 | 0.53 (0.31 to 0.75) | <0.001 | 0.314 | 24.9 | -0.15 to 1.21 | 0.910 |
| **Sensitivity analysis (r = 0.8)** | Luteal / mid-luteal (LP/ML/MLP) | 9 | 55 | 0.39 (0.21 to 0.56) | <0.001 | 0.610 | 46.9 | -0.28 to 1.05 | 0.940 |
| **Sensitivity analysis (r = 0.8)** | Hormonal contraceptive / oral contraceptive (HC/OCP) | 5 | 31 | 0.31 (0.02 to 0.60) | 0.036 | 0.483 | 36.0 | -0.40 to 1.02 | 1.000 |

**Note:** Overall pooled effects came from the primary three-level model. Subgroup Hedges' g, 95% CIs, P within values, P between values, and prediction intervals were aligned to the overall Menstrual2 moderator model. I² and Power for subgroup rows were taken from corresponding stage-specific descriptive models. EF, early follicular; HC/OCP, hormonal contraceptive/oral contraceptive; I², inconsistency statistic; PI, prediction interval; F, omnibus moderator test statistic from the three-level moderator model using t-distribution inference.

**Electronic Supplementary Material Appendix S4 (Table. Exploratory within-stage moderator analyses for selected strata)**

| **Section** | **Stratum** | **Analysis** | **Studies (n)** | **Effect sizes (n)** | **Estimate (95% CI)** | **I² (%)** | **PI** | **Power** | **P within** | **P between** | **Notes** |
| --- | --- | --- | --- | --- | --- | --- | --- | --- | --- | --- | --- |
| **Dose analyses** | Early follicular (EF) | 3 mg/kg | 5 | 23 | 0.45 (0.04 to 0.85) | 71.1 | -0.69 to 1.59 | 0.580 | 0.033 | Ref | Reference category for p between |
| **Dose analyses** | Early follicular (EF) | 4 mg/kg | 3 | 14 | 0.54 (0.02 to 1.07) | 24.7 | 0.00 to 0.95 | 0.830 | 0.044 | 0.773 | p between versus 3 mg/kg |
| **Dose analyses** | Early follicular (EF) | >=5 mg/kg | 2 | 7 | 0.65 (0.01 to 1.30) | 71.2 | -0.90 to 2.22 | 0.530 | 0.047 | 0.585 | p between versus 3 mg/kg |
| **Dose analyses** | Early follicular (EF) | Between-category omnibus test |  |  | F(2, 41) = 0.16 | Not applicable | Not applicable |  |  | 0.854 | Reference category = 3 mg/kg |
| **Dose analyses** | Luteal / mid-luteal (LP/ML/MLP) | 3 mg/kg | 7 | 42 | 0.21 (0.07 to 0.36) | 5.5 | -0.01 to 0.34 | 0.860 | 0.004 | Ref | Reference category for p between |
| **Dose analyses** | Luteal / mid-luteal (LP/ML/MLP) | >=5 mg/kg | 3 | 13 | 0.31 (0.10 to 0.52) | 52.0 | -0.40 to 1.16 | 0.450 | 0.005 | 0.398 | p between versus 3 mg/kg |
| **Dose analyses** | Luteal / mid-luteal (LP/ML/MLP) | Between-category omnibus test |  |  | F(1, 53) = 0.73 | Not applicable | Not applicable |  |  | 0.398 | Reference category = 3 mg/kg |
| **Outlier sensitivity - Dose analyses** | Early follicular (EF) | 3 mg/kg | 5 | 22 | 0.46 (0.10 to 0.83) | 62.9 | -0.49 to 1.42 | 0.590 | 0.013 | Ref | Reference category for p between |
| **Outlier sensitivity - Dose analyses** | Early follicular (EF) | 4 mg/kg | 2 | 12 | 0.41 (-0.14 to 0.96) | 13.8 | 0.02 to 0.76 | 0.740 | 0.136 | 0.870 | p between versus 3 mg/kg |
| **Outlier sensitivity - Dose analyses** | Early follicular (EF) | >=5 mg/kg | 2 | 6 | 0.77 (0.18 to 1.36) | 40.9 | -0.15 to 1.74 | 0.800 | 0.012 | 0.372 | p between versus 3 mg/kg |
| **Outlier sensitivity - Dose analyses** | Early follicular (EF) | Between-category omnibus test |  |  | F(2, 37) = 0.51 | Not applicable | Not applicable |  |  | 0.606 | Reference category = 3 mg/kg |
| **Outlier sensitivity - Dose analyses** | Luteal / mid-luteal (LP/ML/MLP) | 3 mg/kg | 6 | 36 | 0.19 (0.06 to 0.32) | 7.8 | -0.02 to 0.40 | 0.800 | 0.005 | Ref | Reference category for p between |
| **Outlier sensitivity - Dose analyses** | Luteal / mid-luteal (LP/ML/MLP) | >=5 mg/kg | 1 | 3 | 0.58 (0.02 to 1.14) | 0.0 | -0.56 to 1.72 | Not estimable | 0.043 | 0.175 | p between versus 3 mg/kg |
| **Outlier sensitivity - Dose analyses** | Luteal / mid-luteal (LP/ML/MLP) | Between-category omnibus test |  |  | F(1, 37) = 1.91 | Not applicable | Not applicable |  |  | 0.175 | Reference category = 3 mg/kg |
| **Exercise-type analyses** | Early follicular (EF) | Endurance / repeated-output | 7 | 19 | 0.73 (0.45 to 1.01) | 58.5 | -0.08 to 1.78 | 1.000 | <0.001 | Ref | Reference category for p between |
| **Exercise-type analyses** | Early follicular (EF) | Neuromuscular / maximal-output | 8 | 25 | 0.37 (0.12 to 0.62) | 53.2 | -0.33 to 1.07 | 0.730 | 0.005 | 0.013 | p between versus Endurance / repeated-output |
| **Exercise-type analyses** | Early follicular (EF) | Between-category omnibus test |  |  | F(1, 42) = 6.66 | Not applicable | Not applicable |  |  | 0.013 | Reference category = Endurance / repeated-output |
| **Exercise-type analyses** | Luteal / mid-luteal (LP/ML/MLP) | Endurance / repeated-output | 5 | 17 | 0.20 (-0.03 to 0.42) | 32.2 | -0.27 to 0.66 | 0.470 | 0.085 | Ref | Reference category for p between |
| **Exercise-type analyses** | Luteal / mid-luteal (LP/ML/MLP) | Neuromuscular / maximal-output | 8 | 38 | 0.26 (0.11 to 0.41) | 14.2 | -0.02 to 0.54 | 0.880 | 0.001 | 0.602 | p between versus Endurance / repeated-output |
| **Exercise-type analyses** | Luteal / mid-luteal (LP/ML/MLP) | Between-category omnibus test |  |  | F(1, 53) = 0.27 | Not applicable | Not applicable |  |  | 0.602 | Reference category = Endurance / repeated-output |
| **Outlier sensitivity - Exercise-type analyses** | Early follicular (EF) | Endurance / repeated-output | 4 | 13 | 0.70 (0.39 to 1.01) | 28.8 | 0.21 to 1.35 | 0.990 | <0.001 | Ref | Reference category for p between |
| **Outlier sensitivity - Exercise-type analyses** | Early follicular (EF) | Neuromuscular / maximal-output | 8 | 24 | 0.36 (0.14 to 0.59) | 49.0 | -0.26 to 1.02 | 0.770 | 0.002 | 0.043 | p between versus Endurance / repeated-output |
| **Outlier sensitivity - Exercise-type analyses** | Early follicular (EF) | Between-category omnibus test |  |  | F(1, 35) = 4.41 | Not applicable | Not applicable |  |  | 0.043 | Reference category = Endurance / repeated-output |
| **Outlier sensitivity - Exercise-type analyses** | Luteal / mid-luteal (LP/ML/MLP) | Endurance / repeated-output | 3 | 14 | 0.10 (-0.11 to 0.30) | 0.0 | -0.06 to 0.23 | 0.100 | 0.355 | Ref | Reference category for p between |
| **Outlier sensitivity - Exercise-type analyses** | Luteal / mid-luteal (LP/ML/MLP) | Neuromuscular / maximal-output | 7 | 35 | 0.21 (0.08 to 0.34) | 7.5 | 0.00 to 0.42 | 0.830 | 0.002 | 0.348 | p between versus Endurance / repeated-output |
| **Outlier sensitivity - Exercise-type analyses** | Luteal / mid-luteal (LP/ML/MLP) | Between-category omnibus test |  |  | F(1, 47) = 0.90 | Not applicable | Not applicable |  |  | 0.348 | Reference category = Endurance / repeated-output |
| **Phase-verification analyses** | Early follicular (EF) | No verification / cycle counting | 7 | 33 | 0.68 (0.45 to 0.91) | 41.9 | 0.05 to 1.29 | 1.000 | <0.001 | Ref | Reference category for p between |
| **Phase-verification analyses** | Early follicular (EF) | Hormonal verification | 3 | 11 | 0.07 (-0.31 to 0.44) | 44.5 | -0.64 to 0.62 | 0.010 | 0.727 | 0.008 | p between versus No verification / cycle counting |
| **Phase-verification analyses** | Early follicular (EF) | Between-category omnibus test |  |  | F(1, 42) = 7.89 | Not applicable | Not applicable |  |  | 0.008 | Reference category = No verification / cycle counting |
| **Phase-verification analyses** | Luteal / mid-luteal (LP/ML/MLP) | No verification / cycle counting | 5 | 42 | 0.26 (0.07 to 0.44) | 38.8 | -0.23 to 0.78 | 0.700 | 0.008 | Ref | Reference category for p between |
| **Phase-verification analyses** | Luteal / mid-luteal (LP/ML/MLP) | Hormonal verification | 4 | 13 | 0.23 (-0.02 to 0.48) | 0.0 | 0.04 to 0.38 | 0.480 | 0.074 | 0.871 | p between versus No verification / cycle counting |
| **Phase-verification analyses** | Luteal / mid-luteal (LP/ML/MLP) | Between-category omnibus test |  |  | F(1, 53) = 0.03 | Not applicable | Not applicable |  |  | 0.871 | Reference category = No verification / cycle counting |
| **Outlier sensitivity - Phase-verification analyses** | Early follicular (EF) | No verification / cycle counting | 6 | 31 | 0.65 (0.38 to 0.93) | 45.4 | -0.01 to 1.32 | 0.990 | <0.001 | Ref | Reference category for p between |
| **Outlier sensitivity - Phase-verification analyses** | Early follicular (EF) | Hormonal verification | 1 | 7 | 0.04 (-0.60 to 0.67) | 0.0 | -0.22 to 0.30 | Not estimable | 0.908 | 0.079 | p between versus No verification / cycle counting |
| **Outlier sensitivity - Phase-verification analyses** | Early follicular (EF) | Between-category omnibus test |  |  | F(1, 36) = 3.26 | Not applicable | Not applicable |  |  | 0.079 | Reference category = No verification / cycle counting |
| **Outlier sensitivity - Phase-verification analyses** | Luteal / mid-luteal (LP/ML/MLP) | No verification / cycle counting | 4 | 37 | 0.15 (0.00 to 0.29) | 14.8 | -0.13 to 0.43 | 0.520 | 0.048 | Ref | Reference category for p between |
| **Outlier sensitivity - Phase-verification analyses** | Luteal / mid-luteal (LP/ML/MLP) | Hormonal verification | 2 | 10 | 0.20 (-0.05 to 0.45) | 0.0 | -0.02 to 0.40 | 0.250 | 0.108 | 0.706 | p between versus No verification / cycle counting |
| **Outlier sensitivity - Phase-verification analyses** | Luteal / mid-luteal (LP/ML/MLP) | Between-category omnibus test |  |  | F(1, 45) = 0.14 | Not applicable | Not applicable |  |  | 0.706 | Reference category = No verification / cycle counting |
| **Timing-category analyses** | Early follicular (EF) | 60 min | 9 | 38 | 0.46 (0.19 to 0.72) | 57.6 | -0.33 to 1.24 | 0.930 | 0.001 | Ref | Reference category for p between |
| **Timing-category analyses** | Early follicular (EF) | 45 min | 1 | 6 | 0.97 (0.23 to 1.72) | 26.5 | 0.29 to 1.66 | Not estimable | 0.012 | 0.194 | p between versus 60 min |
| **Timing-category analyses** | Early follicular (EF) | Between-category omnibus test |  |  | F(1, 42) = 1.74 | Not applicable | Not applicable |  |  | 0.194 | Reference category = 60 min |
| **Timing-category analyses** | Luteal / mid-luteal (LP/ML/MLP) | 60 min | 8 | 43 | 0.28 (0.13 to 0.43) | 20.3 | -0.05 to 0.61 | 0.930 | <0.001 | Ref | Reference category for p between |
| **Timing-category analyses** | Luteal / mid-luteal (LP/ML/MLP) | 30 min | 1 | 12 | 0.05 (-0.28 to 0.38) | 0.0 | -0.12 to 0.21 | Not estimable | 0.763 | 0.204 | p between versus 60 min |
| **Timing-category analyses** | Luteal / mid-luteal (LP/ML/MLP) | Between-category omnibus test |  |  | F(1, 53) = 1.66 | Not applicable | Not applicable |  |  | 0.204 | Reference category = 60 min |
| **Outlier sensitivity - Timing-category analyses** | Luteal / mid-luteal (LP/ML/MLP) | 60 min | 7 | 38 | 0.20 (0.08 to 0.32) | 7.2 | 0.00 to 0.40 | 0.820 | 0.002 | Ref | Reference category for p between |
| **Outlier sensitivity - Timing-category analyses** | Luteal / mid-luteal (LP/ML/MLP) | 30 min | 1 | 11 | 0.02 (-0.20 to 0.24) | 0.0 | -0.15 to 0.20 | Not estimable | 0.848 | 0.164 | p between versus 60 min |
| **Outlier sensitivity - Timing-category analyses** | Luteal / mid-luteal (LP/ML/MLP) | Between-category omnibus test |  |  | F(1, 47) = 2.00 | Not applicable | Not applicable |  |  | 0.164 | Reference category = 60 min |

**Note:** Main Table 3 is restricted to EF and LP/ML/MLP strata. Timing is treated as a categorical moderator in the main exploratory table. Continuous timing meta-regression is reported in Supplementary Table S3. Outlier sensitivity rows were based on moderator-specific diagnostic models at r = 0.5; therefore, cleaned effect-size counts may differ across dose, exercise-type, phase-verification, and timing analyses and are not expected to match the Table 2 outlier-clean sample sizes. EF, early follicular; LP/ML/MLP, luteal / mid-luteal; I², inconsistency statistic; PI, prediction interval; F, omnibus moderator test statistic from the three-level moderator model using t-distribution inference.

**Electronic Supplementary Material Appendix S5 (Table. Sparse-strata exploratory within-stage moderator analyses for LFP/PO and HC/OCP strata).**

| **Section** | **Stratum** | **Analysis** | **Studies (n)** | **Effect sizes (n)** | **Estimate (95% CI)** | **I² (%)** | **PI** | **Power** | **P within** | **P between** | **Notes** |
| --- | --- | --- | --- | --- | --- | --- | --- | --- | --- | --- | --- |
| **Dose analyses** | **Hormonal contraceptive / oral contraceptive (HC/OCP)** | 3 mg/kg | 3 | 5 | 0.28 (0.00 to 0.55) | 0.0 | -0.03 to 0.64 | 0.260 | 0.047 | Ref | Reference category for p between |
| **Dose analyses** | **Hormonal contraceptive / oral contraceptive (HC/OCP)** | >=5 mg/kg | 3 | 26 | 0.25 (0.06 to 0.44) | 12.6 | -0.07 to 0.60 | 0.770 | 0.013 | 0.845 | p between versus 3 mg/kg |
| **Dose analyses** | **Hormonal contraceptive / oral contraceptive (HC/OCP)** | **Between-category omnibus test** |  |  | F(1, 29) = 0.04 | Not applicable | Not applicable |  |  | 0.845 | Reference category = 3 mg/kg |
| **Dose analyses** | **Late follicular / peri-ovulatory (LFP/PO)** | 3 mg/kg | 3 | 11 | 0.25 (0.06 to 0.44) | 0.0 | 0.06 to 0.44 | 0.510 | 0.014 | Ref | Reference category for p between |
| **Dose analyses** | **Late follicular / peri-ovulatory (LFP/PO)** | >=5 mg/kg | 1 | 3 | 0.51 (-0.09 to 1.11) | 0.0 | -0.68 to 1.70 | Not estimable | 0.090 | 0.382 | p between versus 3 mg/kg |
| **Dose analyses** | **Late follicular / peri-ovulatory (LFP/PO)** | **Between-category omnibus test** |  |  | F(1, 12) = 0.82 | Not applicable | Not applicable |  |  | 0.382 | Reference category = 3 mg/kg |
| **Outlier sensitivity - Dose analyses** | **Late follicular / peri-ovulatory (LFP/PO)** | 3 mg/kg | 3 | 10 | 0.21 (0.01 to 0.41) | 0.0 | 0.01 to 0.41 | 0.320 | 0.039 | Ref | Reference category for p between |
| **Outlier sensitivity - Dose analyses** | **Late follicular / peri-ovulatory (LFP/PO)** | >=5 mg/kg | 1 | 3 | 0.51 (-0.10 to 1.12) | 0.0 | -0.68 to 1.70 | Not estimable | 0.092 | 0.322 | p between versus 3 mg/kg |
| **Outlier sensitivity - Dose analyses** | **Late follicular / peri-ovulatory (LFP/PO)** | **Between-category omnibus test** |  |  | F(1, 11) = 1.08 | Not applicable | Not applicable |  |  | 0.322 | Reference category = 3 mg/kg |
| **Exercise-type analyses** | **Hormonal contraceptive / oral contraceptive (HC/OCP)** | Endurance / repeated-output | 4 | 7 | 0.47 (0.25 to 0.69) | 18.1 | -0.03 to 0.90 | 0.780 | <0.001 | Ref | Reference category for p between |
| **Exercise-type analyses** | **Hormonal contraceptive / oral contraceptive (HC/OCP)** | Neuromuscular / maximal-output | 2 | 24 | 0.15 (0.02 to 0.28) | 0.0 | 0.02 to 0.28 | 0.390 | 0.028 | Not estimable | p between versus Endurance / repeated-output not estimable because the comparison model was sparse or singular |
| **Exercise-type analyses** | **Hormonal contraceptive / oral contraceptive (HC/OCP)** | **Between-category omnibus test** |  |  | Not estimable | Not applicable | Not applicable |  |  |  | Reference category = Endurance / repeated-output |
| **Exercise-type analyses** | **Late follicular / peri-ovulatory (LFP/PO)** | Endurance / repeated-output | 1 | 1 | 0.36 (-0.67 to 1.40) | Not estimable | Not estimable | Not estimable | 0.461 | Ref | Reference category for p between |
| **Exercise-type analyses** | **Late follicular / peri-ovulatory (LFP/PO)** | Neuromuscular / maximal-output | 4 | 13 | 0.27 (0.09 to 0.45) | 0.0 | 0.09 to 0.45 | 0.750 | 0.007 | 0.849 | p between versus Endurance / repeated-output |
| **Exercise-type analyses** | **Late follicular / peri-ovulatory (LFP/PO)** | **Between-category omnibus test** |  |  | F(1, 12) = 0.04 | Not applicable | Not applicable |  |  | 0.849 | Reference category = Endurance / repeated-output |
| **Outlier sensitivity - Exercise-type analyses** | **Hormonal contraceptive / oral contraceptive (HC/OCP)** | Endurance / repeated-output | 3 | 6 | 0.54 (0.29 to 0.80) | Not estimable | Not estimable | Not estimable | <0.001 | Ref | Reference category for p between |
| **Outlier sensitivity - Exercise-type analyses** | **Hormonal contraceptive / oral contraceptive (HC/OCP)** | Neuromuscular / maximal-output | 2 | 24 | 0.15 (0.02 to 0.28) | 0.0 | 0.02 to 0.28 | 0.390 | 0.028 | 0.008 | p between versus Endurance / repeated-output |
| **Outlier sensitivity - Exercise-type analyses** | **Hormonal contraceptive / oral contraceptive (HC/OCP)** | **Between-category omnibus test** |  |  | F(1, 28) = 8.17 | Not applicable | Not applicable |  |  | 0.008 | Reference category = Endurance / repeated-output |
| **Phase-verification analyses** | **Hormonal contraceptive / oral contraceptive (HC/OCP)** | No verification / cycle counting | 4 | 30 | 0.26 (0.09 to 0.43) | 8.7 | 0.00 to 0.52 | 0.700 | 0.005 | Ref | Reference category for p between |
| **Phase-verification analyses** | **Hormonal contraceptive / oral contraceptive (HC/OCP)** | Hormonal verification | 1 | 1 | 0.26 (-0.39 to 0.92) | Not estimable | Not estimable | Not estimable | 0.416 | 0.983 | p between versus No verification / cycle counting |
| **Phase-verification analyses** | **Hormonal contraceptive / oral contraceptive (HC/OCP)** | **Between-category omnibus test** |  |  | F(1, 29) = 0.00 | Not applicable | Not applicable |  |  | 0.983 | Reference category = No verification / cycle counting |
| **Phase-verification analyses** | **Late follicular / peri-ovulatory (LFP/PO)** | No verification / cycle counting | 1 | 3 | 0.51 (-0.09 to 1.11) | 0.0 | -0.68 to 1.70 | Not estimable | 0.090 | Ref | Reference category for p between |
| **Phase-verification analyses** | **Late follicular / peri-ovulatory (LFP/PO)** | Hormonal verification | 3 | 11 | 0.25 (0.06 to 0.44) | 0.0 | 0.06 to 0.44 | 0.510 | 0.014 | 0.382 | p between versus No verification / cycle counting |
| **Phase-verification analyses** | **Late follicular / peri-ovulatory (LFP/PO)** | **Between-category omnibus test** |  |  | F(1, 12) = 0.82 | Not applicable | Not applicable |  |  | 0.382 | Reference category = No verification / cycle counting |
| **Outlier sensitivity - Phase-verification analyses** | **Late follicular / peri-ovulatory (LFP/PO)** | No verification / cycle counting | 1 | 3 | 0.51 (-0.10 to 1.12) | 0.0 | -0.68 to 1.70 | Not estimable | 0.092 | Ref | Reference category for p between |
| **Outlier sensitivity - Phase-verification analyses** | **Late follicular / peri-ovulatory (LFP/PO)** | Hormonal verification | 3 | 10 | 0.21 (0.01 to 0.41) | 0.0 | 0.01 to 0.41 | 0.320 | 0.039 | 0.322 | p between versus No verification / cycle counting |
| **Outlier sensitivity - Phase-verification analyses** | **Late follicular / peri-ovulatory (LFP/PO)** | **Between-category omnibus test** |  |  | F(1, 11) = 1.08 | Not applicable | Not applicable |  |  | 0.322 | Reference category = No verification / cycle counting |
| **Timing-category analyses** | **Hormonal contraceptive / oral contraceptive (HC/OCP)** | 60 min | 3 | 24 | 0.18 (0.05 to 0.31) | Not estimable | Not estimable | Not estimable | 0.010 | Ref | Reference category for p between |
| **Timing-category analyses** | **Hormonal contraceptive / oral contraceptive (HC/OCP)** | 30 min | 1 | 6 | 0.40 (0.16 to 0.64) | 29.3 | -0.18 to 1.00 | Not estimable | 0.002 | 0.104 | p between versus 60 min |
| **Timing-category analyses** | **Hormonal contraceptive / oral contraceptive (HC/OCP)** | 90 min | 1 | 1 | 0.26 (-0.36 to 0.89) | Not estimable | Not estimable | Not estimable | 0.395 | 0.783 | p between versus 60 min |
| **Timing-category analyses** | **Hormonal contraceptive / oral contraceptive (HC/OCP)** | **Between-category omnibus test** |  |  | F(2, 28) = 1.42 | Not applicable | Not applicable |  |  | 0.258 | Reference category = 60 min |
| **Outlier sensitivity - Timing-category analyses** | **Hormonal contraceptive / oral contraceptive (HC/OCP)** | 60 min | 3 | 24 | 0.18 (0.05 to 0.31) | Not estimable | Not estimable | Not estimable | 0.010 | Ref | Reference category for p between |
| **Outlier sensitivity - Timing-category analyses** | **Hormonal contraceptive / oral contraceptive (HC/OCP)** | 30 min | 1 | 6 | 0.40 (0.16 to 0.64) | 29.3 | -0.18 to 1.00 | Not estimable | 0.002 | 0.104 | p between versus 60 min |
| **Outlier sensitivity - Timing-category analyses** | **Hormonal contraceptive / oral contraceptive (HC/OCP)** | **Between-category omnibus test** |  |  | F(1, 28) = 2.83 | Not applicable | Not applicable |  |  | 0.104 | Reference category = 60 min |
| **Continuous timing meta-regression** | **Early follicular (EF)** | Timing slope (per min) | 10 | 44 | -0.03 (-0.09 to 0.02) | 57.7 | Not applicable | 0.250 |  | 0.194 | Continuous exploratory meta-regression; timing range 45-60 min. P between reports the test for the continuous timing slope. |
| **Continuous timing meta-regression** | **Hormonal contraceptive / oral contraceptive (HC/OCP)** | Timing slope (per min) | 5 | 31 | -0.01 (-0.01 to 0.00) | 0.0 | Not applicable | 0.180 |  | 0.161 | Continuous exploratory meta-regression; timing range 30-90 min. P between reports the test for the continuous timing slope. |
| **Continuous timing meta-regression** | **Luteal / mid-luteal (LP/ML/MLP)** | Timing slope (per min) | 9 | 55 | 0.01 (0.00 to 0.02) | 21.0 | Not applicable | 0.380 |  | 0.204 | Continuous exploratory meta-regression; timing range 30-60 min. P between reports the test for the continuous timing slope. |
| **Outlier sensitivity - Continuous timing meta-regression** | **Luteal / mid-luteal (LP/ML/MLP)** | Timing slope (per min) | 8 | 49 | 0.01 (0.00 to 0.01) | 7.5 | Not applicable | 0.350 |  | 0.164 | Continuous exploratory meta-regression; timing range 30-60 min. P between reports the test for the continuous timing slope. |
| **Habitual caffeine intake analyses** | **Overall** | Low | 14 | 102 | 0.37 (0.21 to 0.53) | 44.1 | -0.17 to 0.91 | 1.000 | <0.001 | Ref | Reference category for p between Exploratory study-level moderator; intake categories were unevenly distributed across menstrual-cycle and contraceptive strata. |
| **Habitual caffeine intake analyses** | **Overall** | Moderate | 5 | 35 | 0.42 (0.14 to 0.70) | 40.7 | -0.21 to 1.04 | 0.800 | 0.004 | 0.763 | p between versus Low Exploratory study-level moderator; intake categories were unevenly distributed across menstrual-cycle and contraceptive strata. |
| **Habitual caffeine intake analyses** | **Overall** | High | 1 | 7 | 0.29 (-0.26 to 0.85) | 0.0 | 0.05 to 0.54 | Not estimable | 0.298 | 0.798 | p between versus Low Single-study category; interpreted descriptively. Exploratory study-level moderator; intake categories were unevenly distributed across menstrual-cycle and contraceptive strata. |
| **Habitual caffeine intake analyses** | **Overall** | **Between-category omnibus test** |  |  | F(2, 141) = 0.09 | Not applicable | Not applicable |  |  | 0.913 | Reference category = Low |
| **Habitual caffeine intake analyses** | **Early follicular (EF)** | Low | 7 | 26 | 0.49 (0.15 to 0.83) | 73.4 | -0.64 to 1.66 | 0.840 | 0.006 | Ref | Reference category for p between |
| **Habitual caffeine intake analyses** | **Early follicular (EF)** | Moderate | 2 | 11 | 0.72 (0.11 to 1.33) | 32.8 | 0.05 to 1.40 | 0.790 | 0.022 | 0.514 | p between versus Low |
| **Habitual caffeine intake analyses** | **Early follicular (EF)** | High | 1 | 7 | 0.29 (-0.53 to 1.12) | 0.0 | 0.05 to 0.54 | Not estimable | 0.475 | 0.659 | p between versus Low Single-study category; interpreted descriptively. |
| **Habitual caffeine intake analyses** | **Early follicular (EF)** | **Between-category omnibus test** |  |  | F(2, 41) = 0.38 | Not applicable | Not applicable |  |  | 0.684 | Reference category = Low |
| **Habitual caffeine intake analyses** | **Hormonal contraceptive / oral contraceptive (HC/OCP)** | Low | 2 | 7 | Not estimable | Not estimable | Not estimable | Not estimable |  | Not estimable | Sparse within-stage comparison; interpreted descriptively. Model could not be estimated. |
| **Habitual caffeine intake analyses** | **Hormonal contraceptive / oral contraceptive (HC/OCP)** | Moderate | 3 | 24 | Not estimable | Not estimable | Not estimable | Not estimable |  | Not estimable | Sparse within-stage comparison; interpreted descriptively. Model could not be estimated. |
| **Habitual caffeine intake analyses** | **Hormonal contraceptive / oral contraceptive (HC/OCP)** | **Between-category omnibus test** |  |  | Not estimable | Not applicable | Not applicable |  |  | Not estimable | Sparse within-stage comparison; interpreted descriptively. Between-category model could not be estimated. |
| **Habitual caffeine intake analyses** | **Late follicular / peri-ovulatory (LFP/PO)** | Low only | 4 | 14 | Not estimable | Not estimable | Not estimable | Not estimable |  | Not applicable | Not tested for between-category differences because only Low habitual caffeine intake was represented. |
| **Habitual caffeine intake analyses** | **Luteal / mid-luteal (LP/ML/MLP)** | Low only | 9 | 55 | Not estimable | Not estimable | Not estimable | Not estimable |  | Not applicable | Not tested for between-category differences because only Low habitual caffeine intake was represented. |
| **Outlier sensitivity - Habitual caffeine intake analyses** | **Overall** | Low | 13 | 96 | 0.34 (0.18 to 0.50) | 39.2 | -0.15 to 0.82 | 0.990 | <0.001 | Ref | Reference category for p between Exploratory study-level moderator; intake categories were unevenly distributed across menstrual-cycle and contraceptive strata. |
| **Outlier sensitivity - Habitual caffeine intake analyses** | **Overall** | Moderate | 3 | 30 | 0.48 (0.16 to 0.81) | 47.4 | -0.26 to 1.26 | 0.660 | 0.004 | 0.431 | p between versus Low Exploratory study-level moderator; intake categories were unevenly distributed across menstrual-cycle and contraceptive strata. |
| **Outlier sensitivity - Habitual caffeine intake analyses** | **Overall** | **Between-category omnibus test** |  |  | F(1, 124) = 0.62 | Not applicable | Not applicable |  |  | 0.431 | Reference category = Low |
| **Outlier sensitivity - Habitual caffeine intake analyses** | **Early follicular (EF)** | Low | 5 | 22 | 0.50 (0.12 to 0.87) | 65.7 | -0.49 to 1.49 | 0.670 | 0.010 | Ref | Reference category for p between |
| **Outlier sensitivity - Habitual caffeine intake analyses** | **Early follicular (EF)** | Moderate | 2 | 10 | 0.81 (0.22 to 1.41) | 0.0 | 0.55 to 1.09 | 0.970 | 0.009 | 0.370 | p between versus Low |
| **Outlier sensitivity - Habitual caffeine intake analyses** | **Early follicular (EF)** | High | 1 | 7 | 0.29 (-0.50 to 1.09) | 0.0 | 0.05 to 0.54 | Not estimable | 0.461 | 0.638 | p between versus Low Single-study category; interpreted descriptively. |
| **Outlier sensitivity - Habitual caffeine intake analyses** | **Early follicular (EF)** | **Between-category omnibus test** |  |  | F(2, 36) = 0.66 | Not applicable | Not applicable |  |  | 0.525 | Reference category = Low |

**Note:** These sparse-strata results are kept outside the main table because the within-stratum evidence base was limited. Continuous timing rows report exploratory timing slope models and their r = 0.5 outlier-clean sensitivity analyses. Habitual caffeine intake was examined as an exploratory study-level moderator. Intake categories were unevenly distributed across menstrual-cycle and hormonal-contraceptive strata; therefore, within-stage habitual-caffeine analyses should be interpreted descriptively. In outlier-clean habitual-caffeine analyses, categories not retained after model-specific outlier exclusion were not included in the corresponding omnibus comparison. Categorical omnibus tests are reported as F statistics from three-level moderator models using t-distribution inference. Continuous timing rows report slope estimates and the corresponding moderator test p-values.

**Electronic Supplementary Material Appendix S6 (Leave-one-out sensitivity analysis for the overall model)**


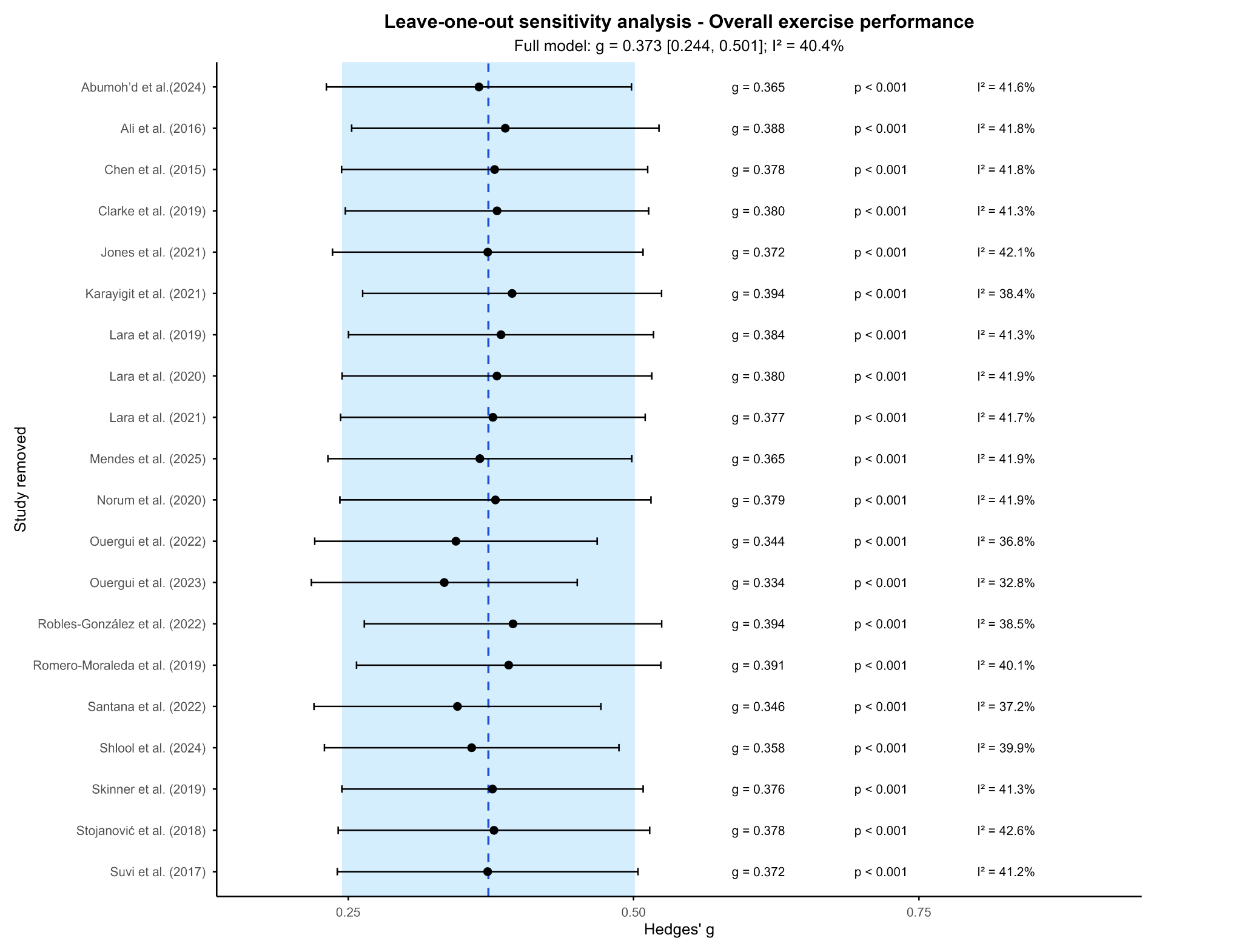


**Electronic Supplementary Material S7 (Leave-one-out sensitivity analyses for menstrual-cycle and hormonal-contraceptive subgroups)**

| **Early follicular (EF)**  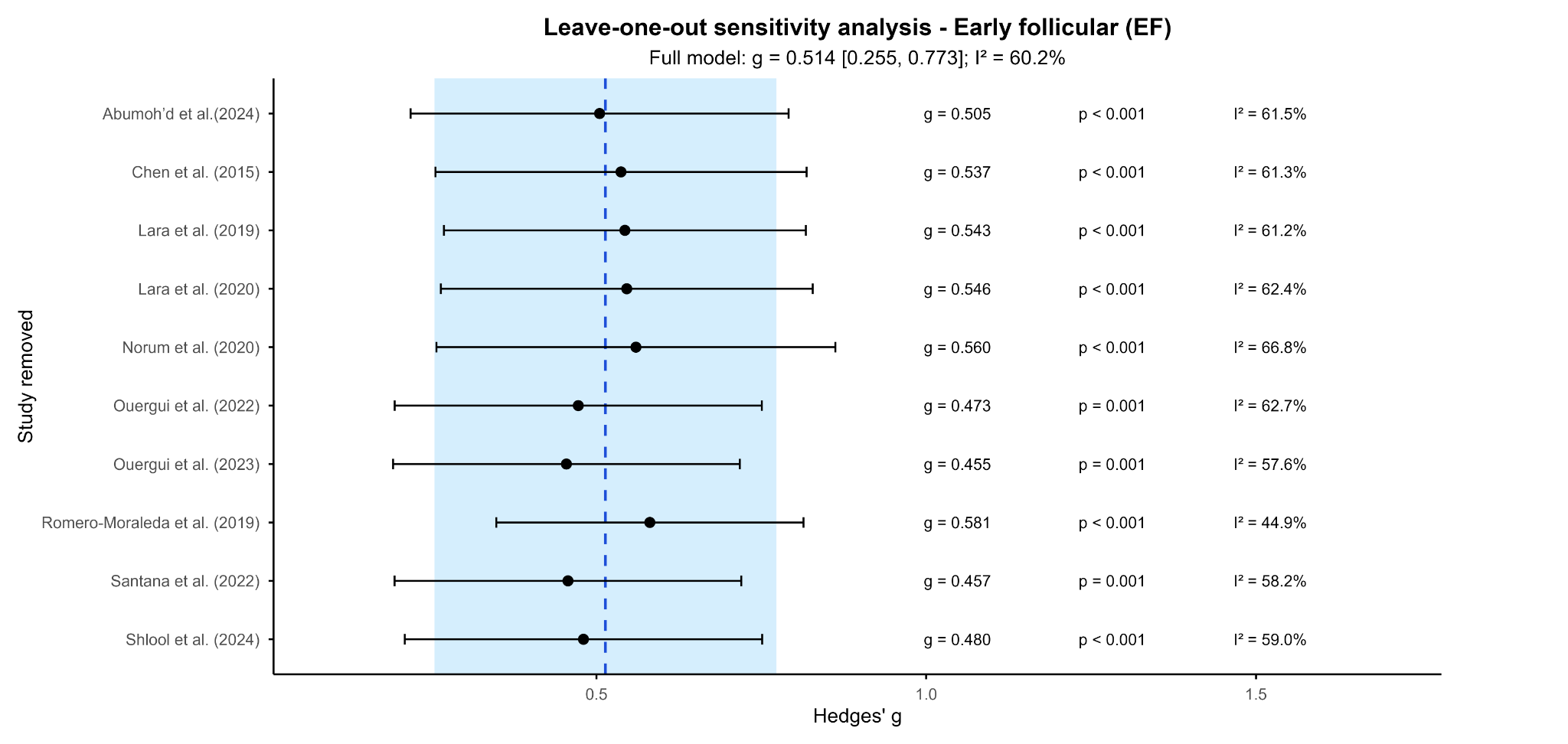 | **Hormonal contraceptive / oral contraceptive users (HC/OCP)**  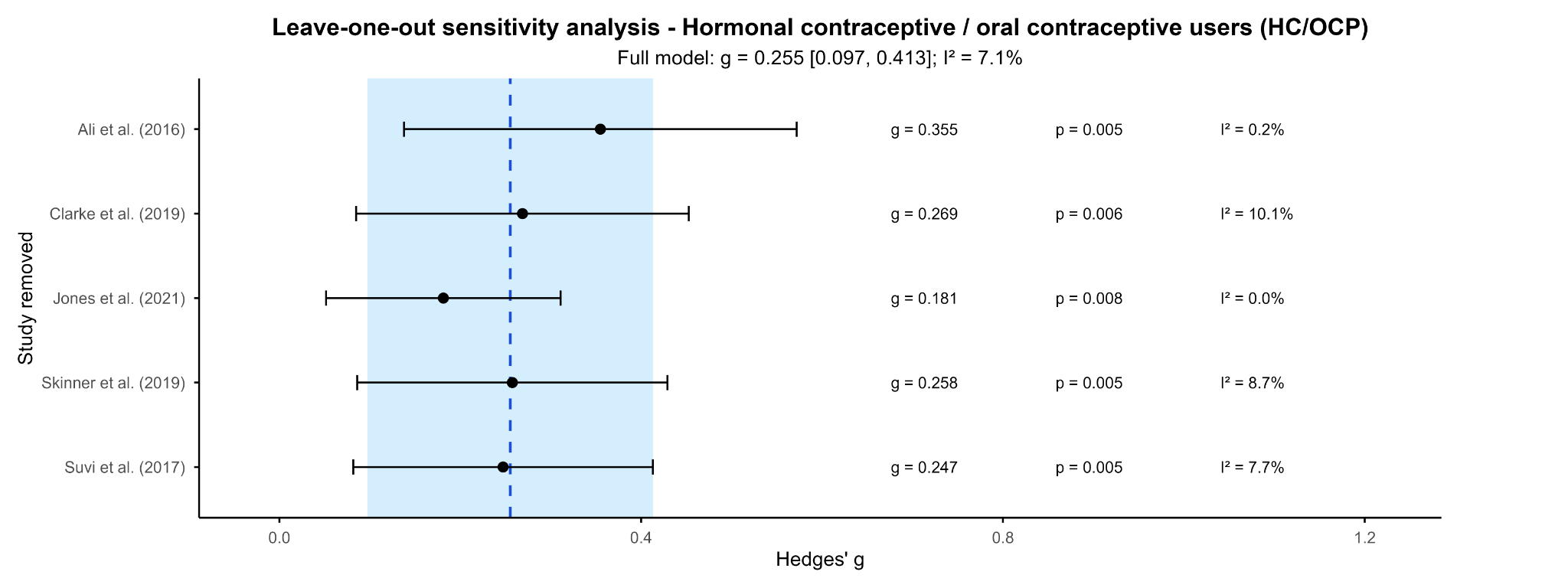 |
| --- | --- |
| **Late follicular / peri-ovulatory (LFP/PO)**  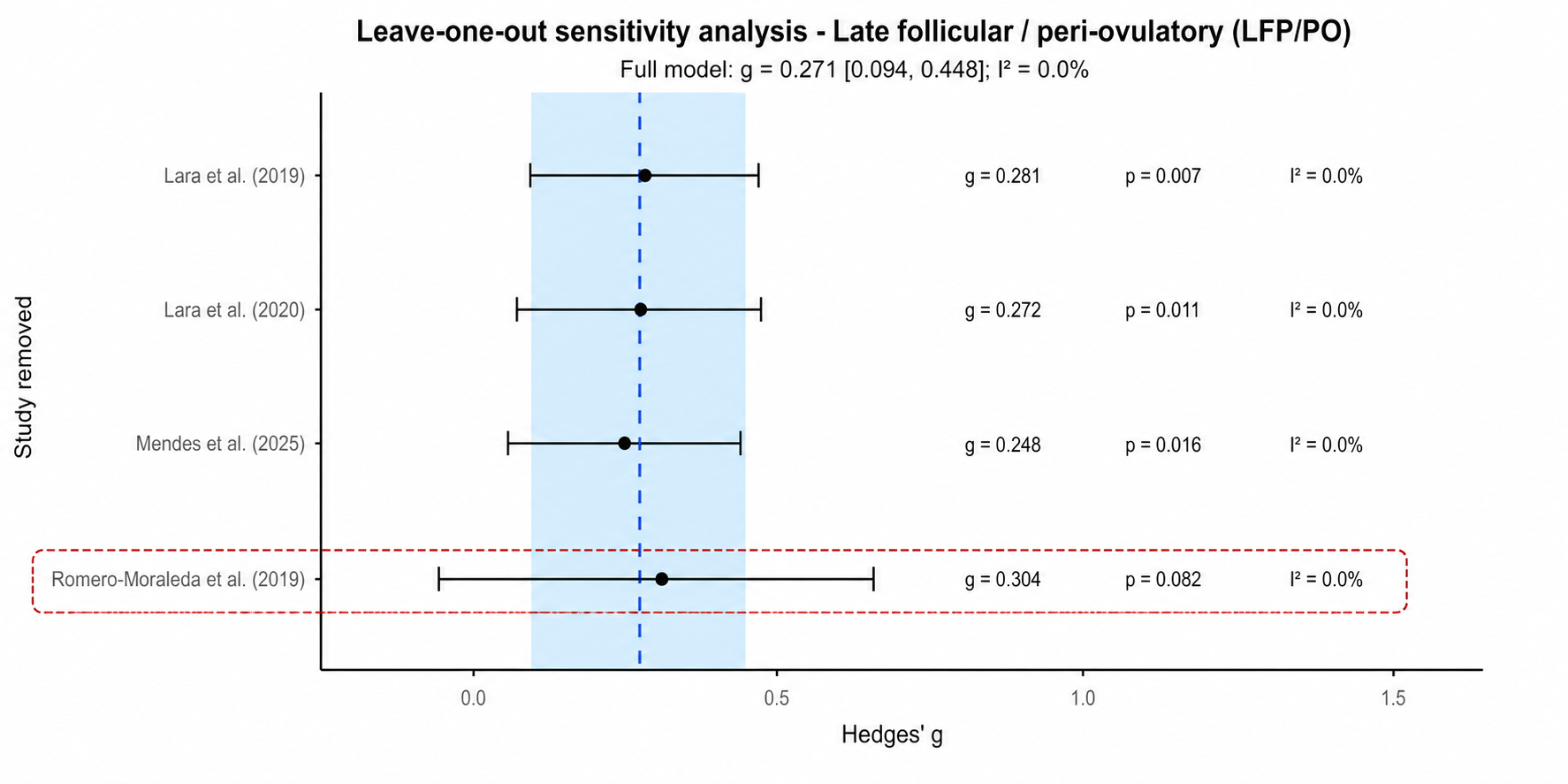 | **Luteal / mid-luteal (LP/ML/MLP)**  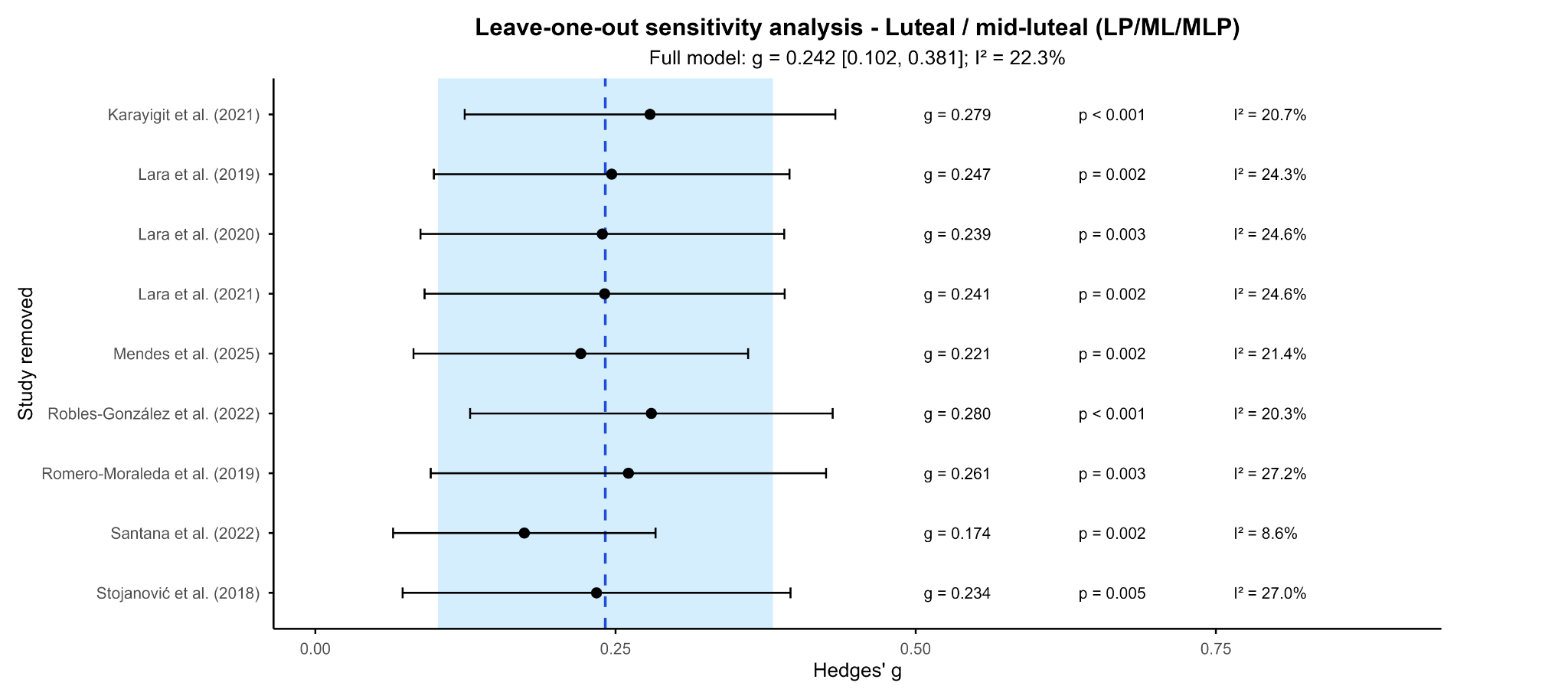 |

Notes: The first section shows the leave-one-out sensitivity analysis for the overall exercise-performance model. The second section shows the corresponding leave-one-out sensitivity analyses for the four menstrual-cycle/contraceptive subgroups.

**Electronic Supplementary Material Appendix S8 (RoB 2 Assessment of Risk of Bias in the Included Studies)**

**
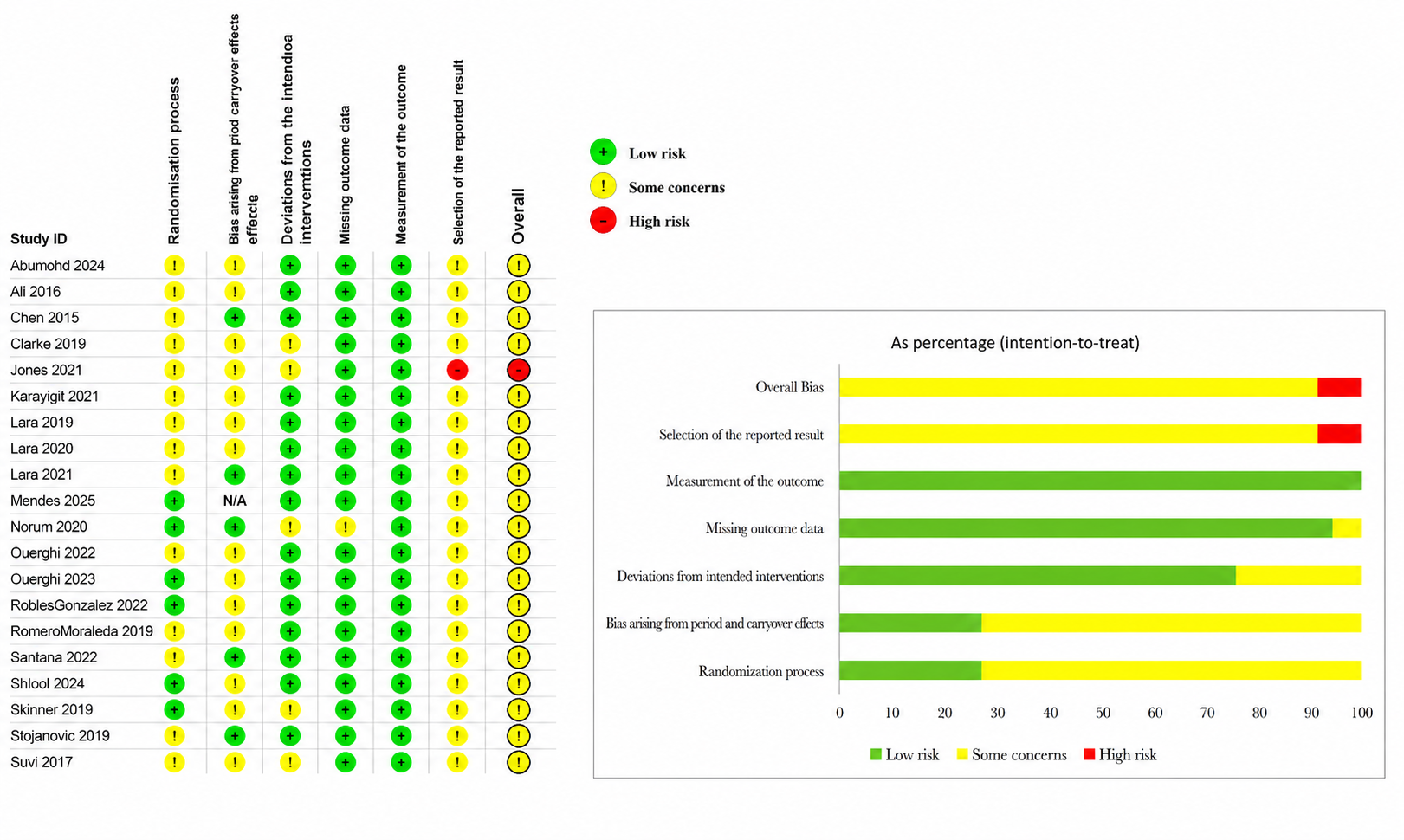
**

**Electronic Supplementary Material Appendix S9 (Contour-enhanced funnel plots for primary, subgroup, and exploratory analyses)**
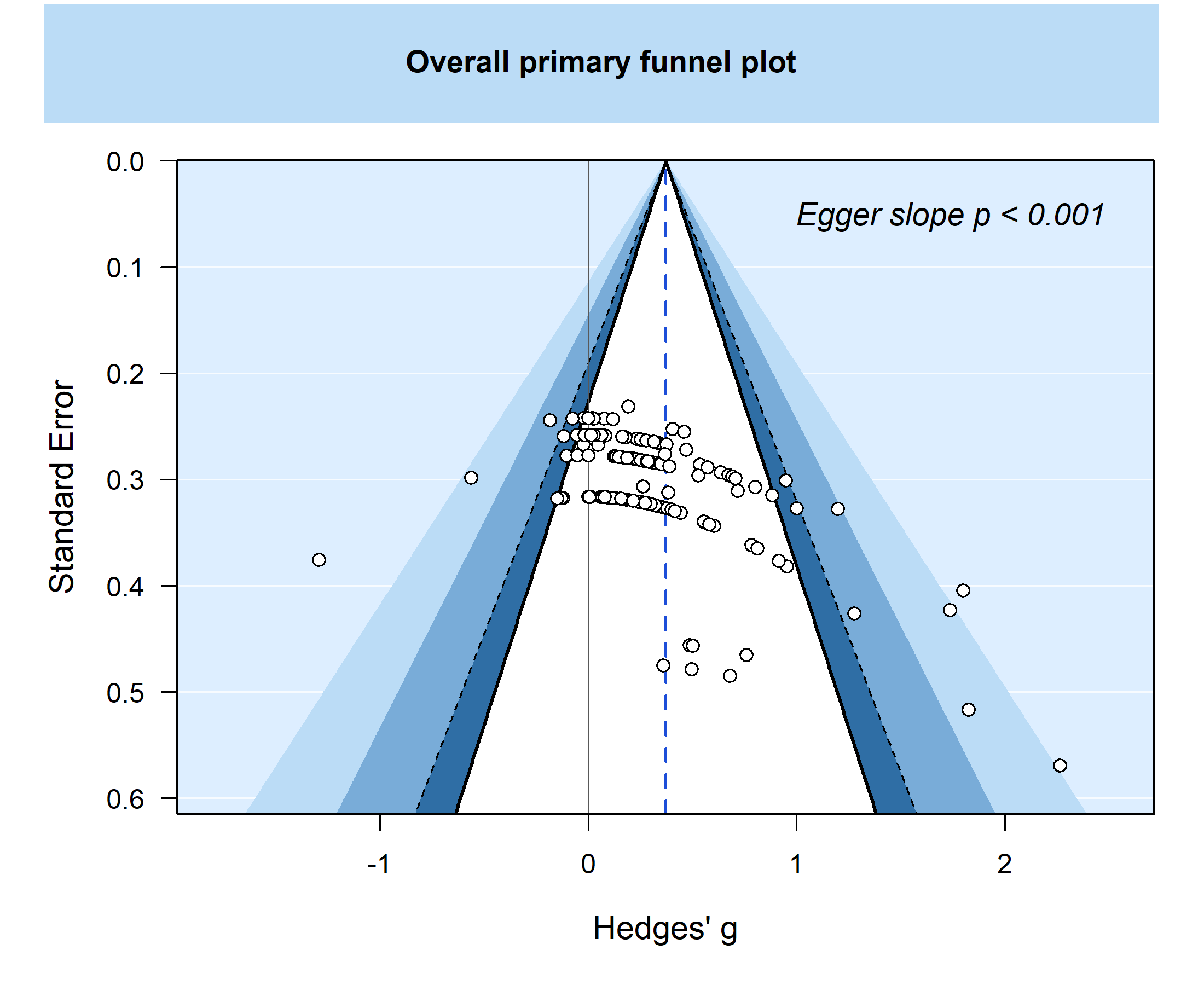


| **A**  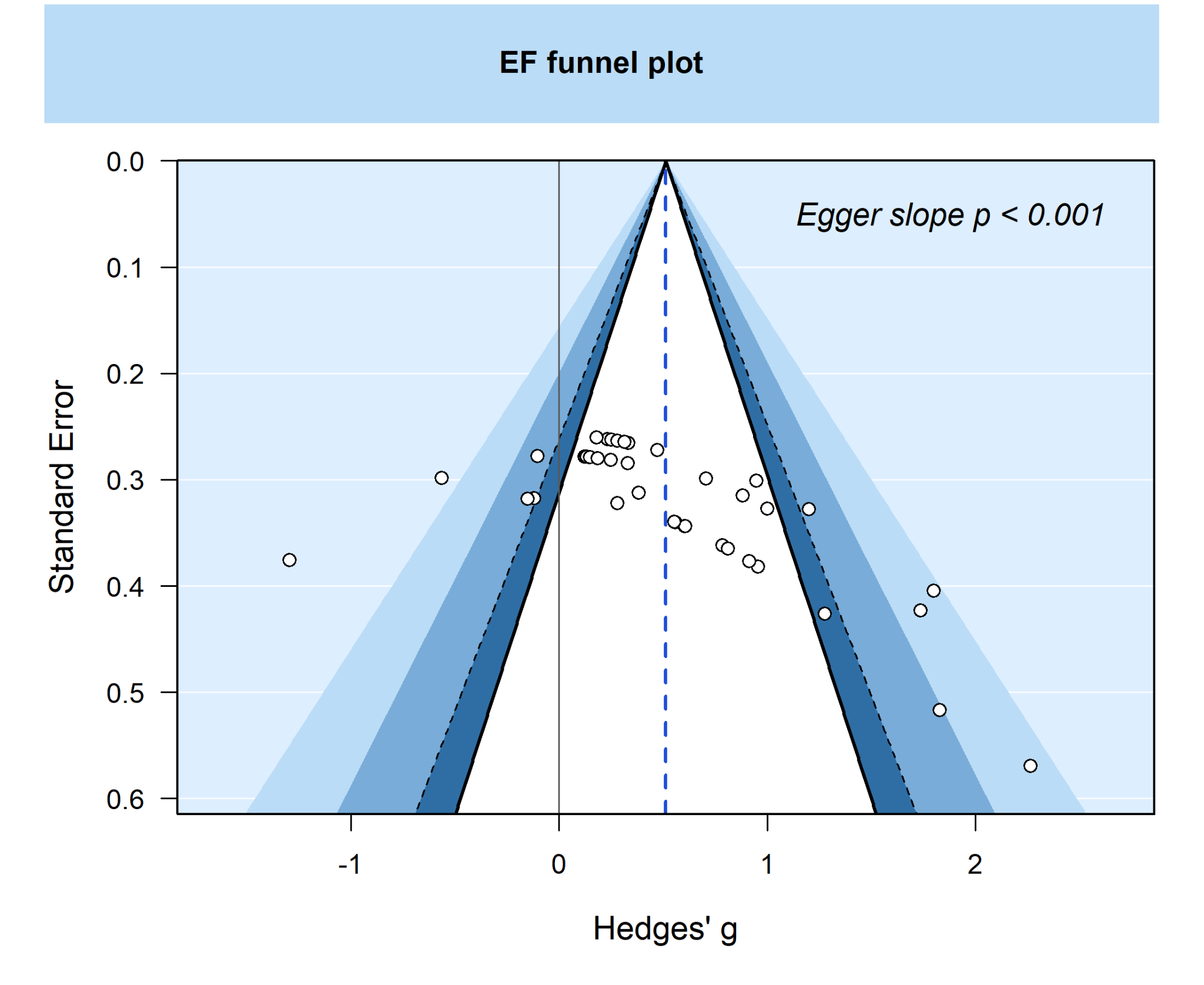 | **B**  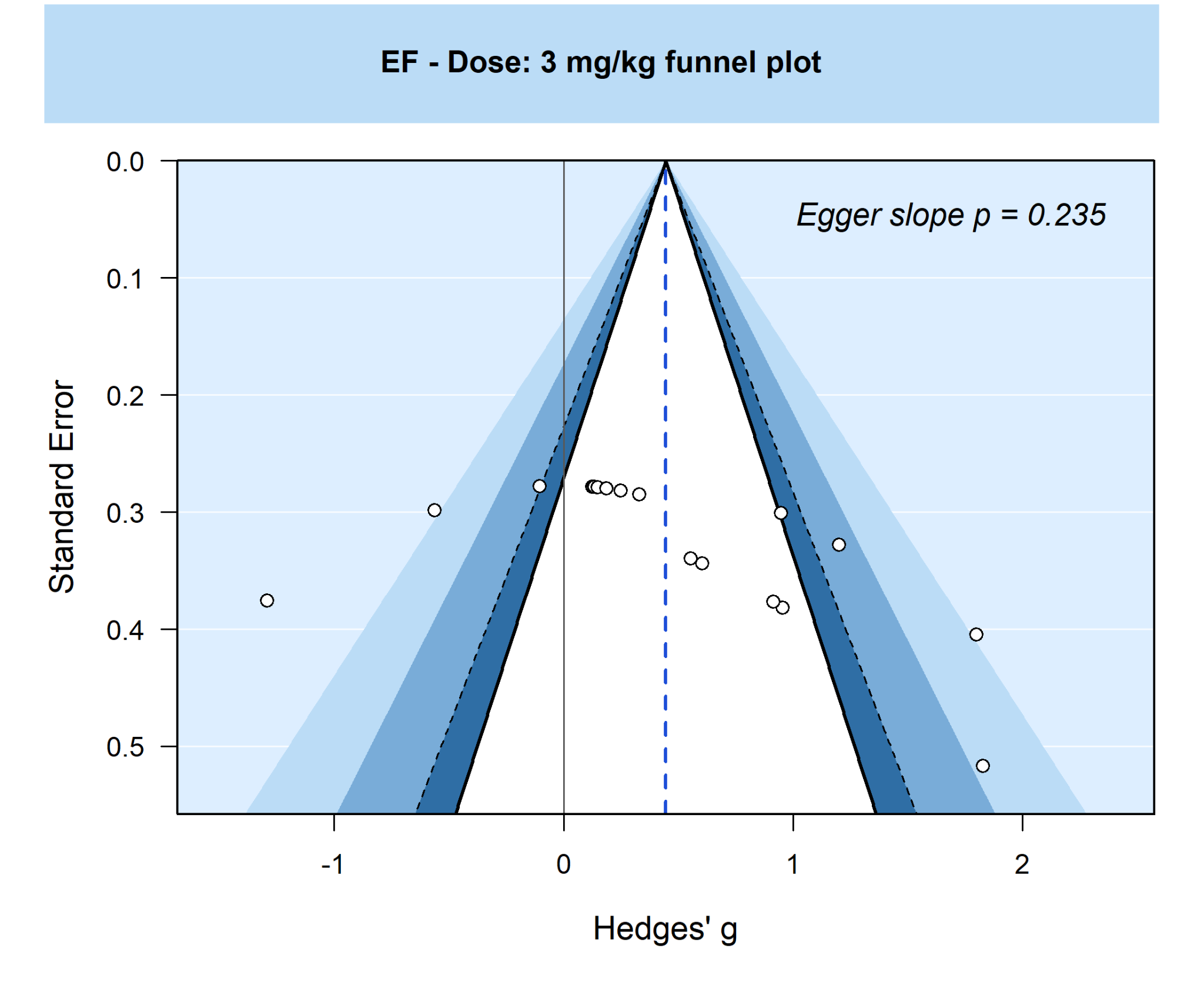 | **C**  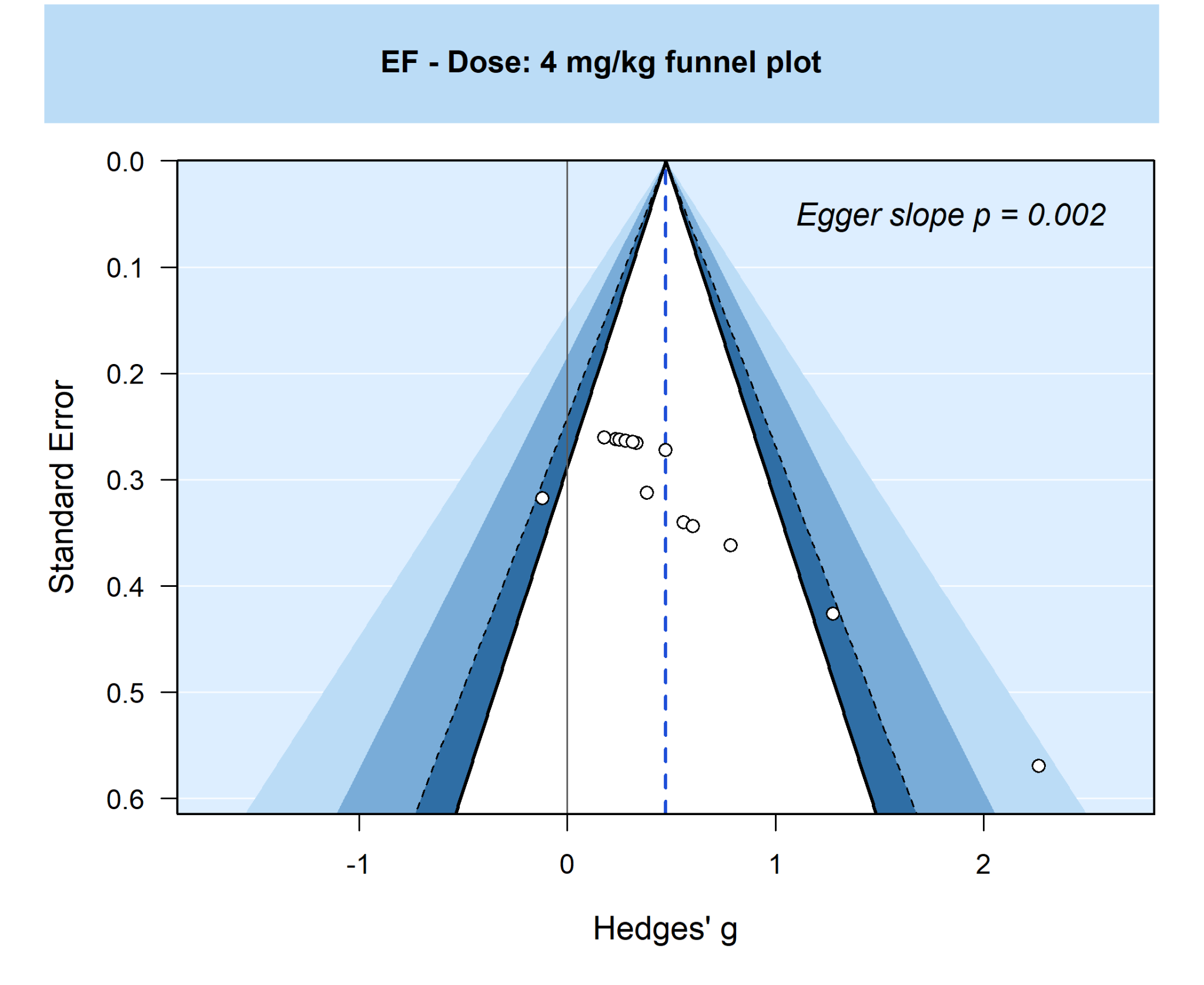 | **D**  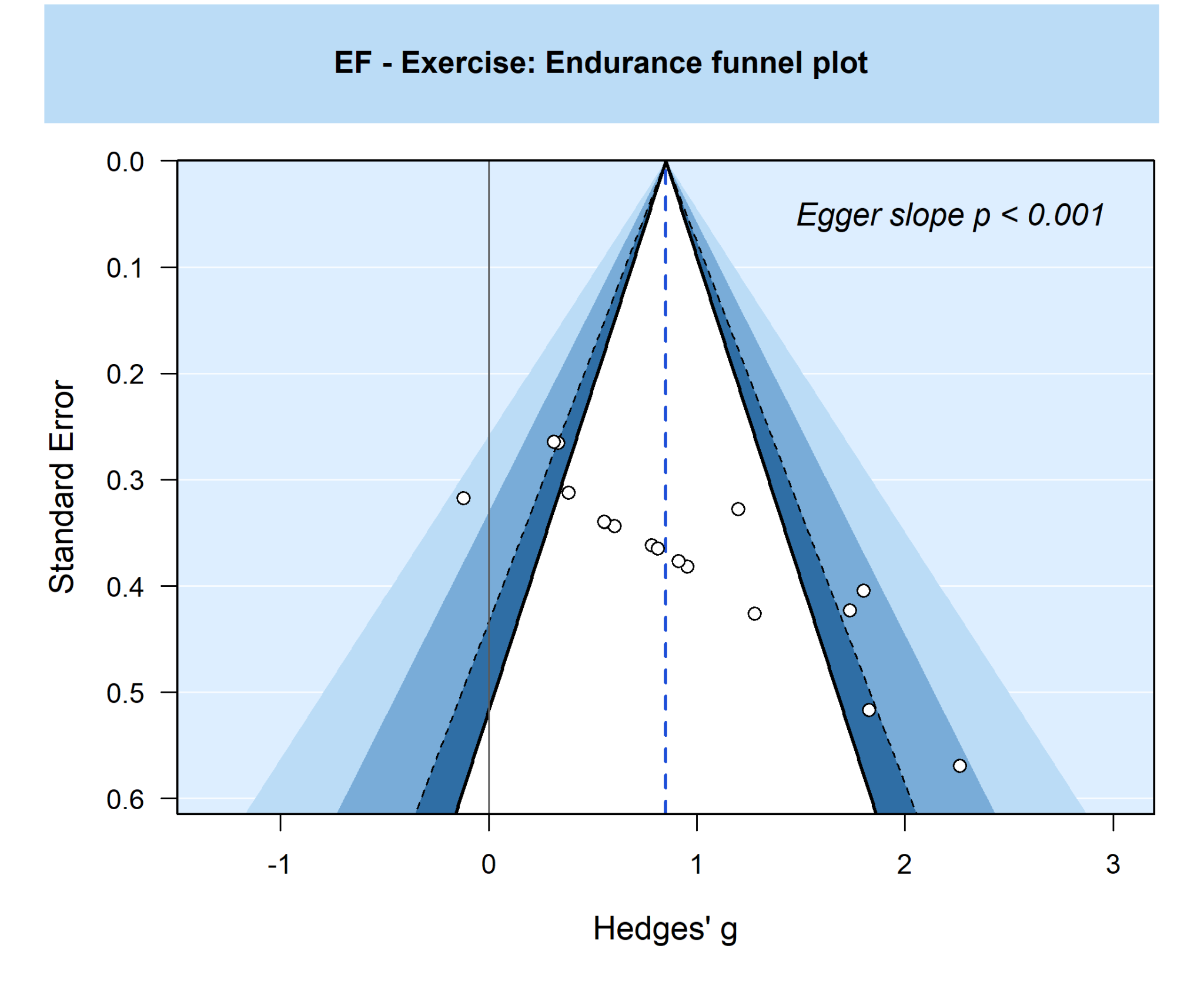 |
| --- | --- | --- | --- |
| **E**  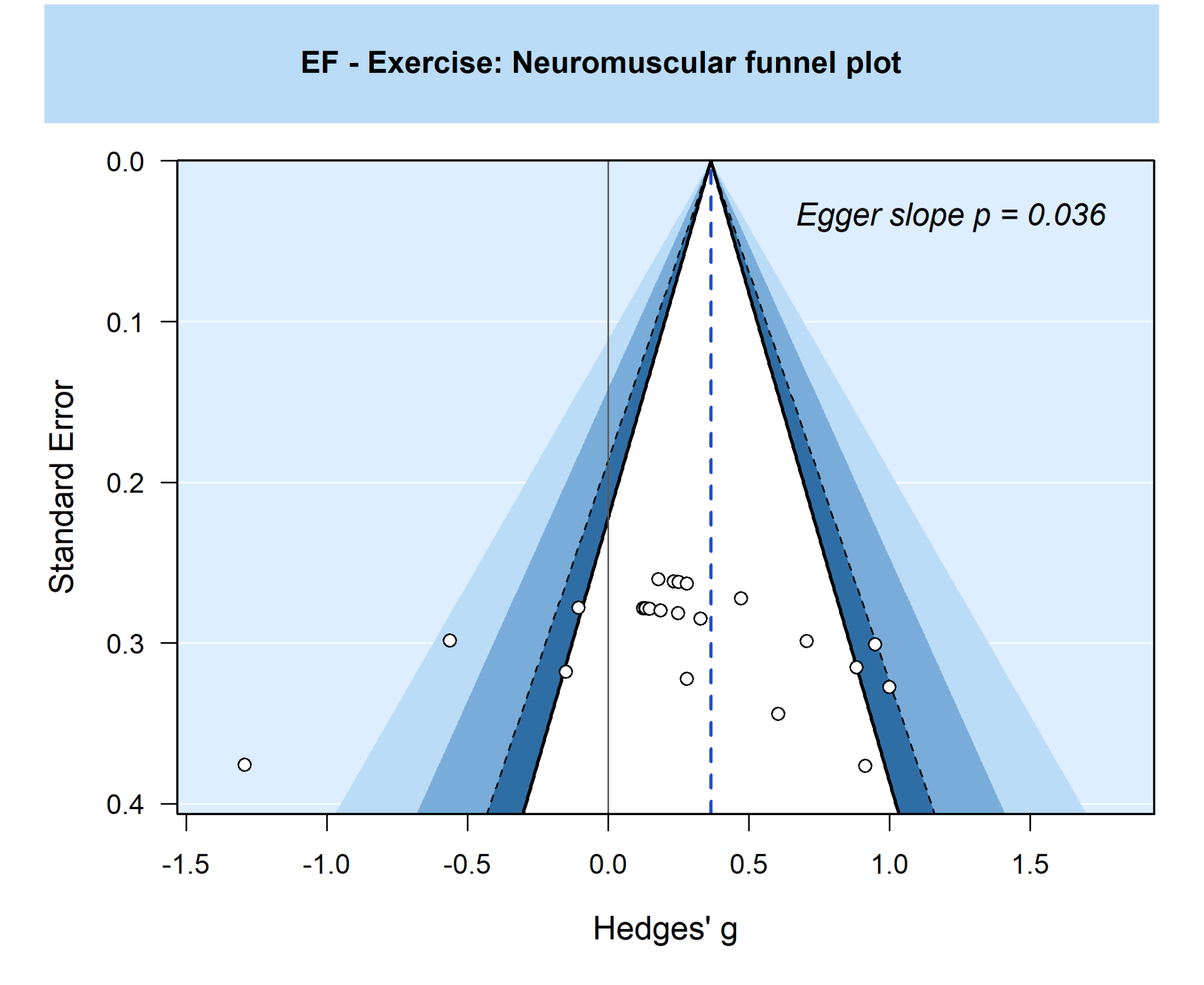 | **F**  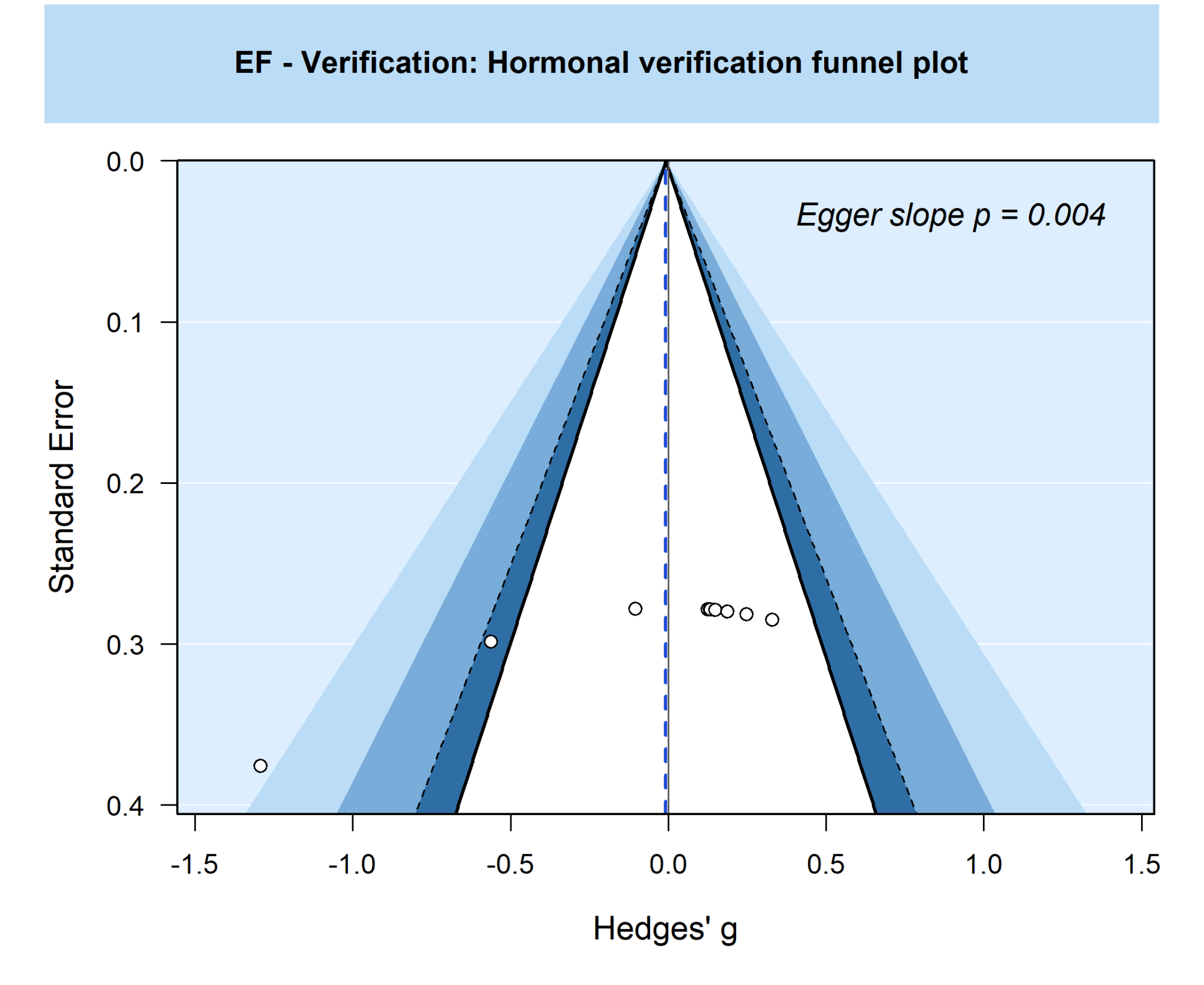 | **G**  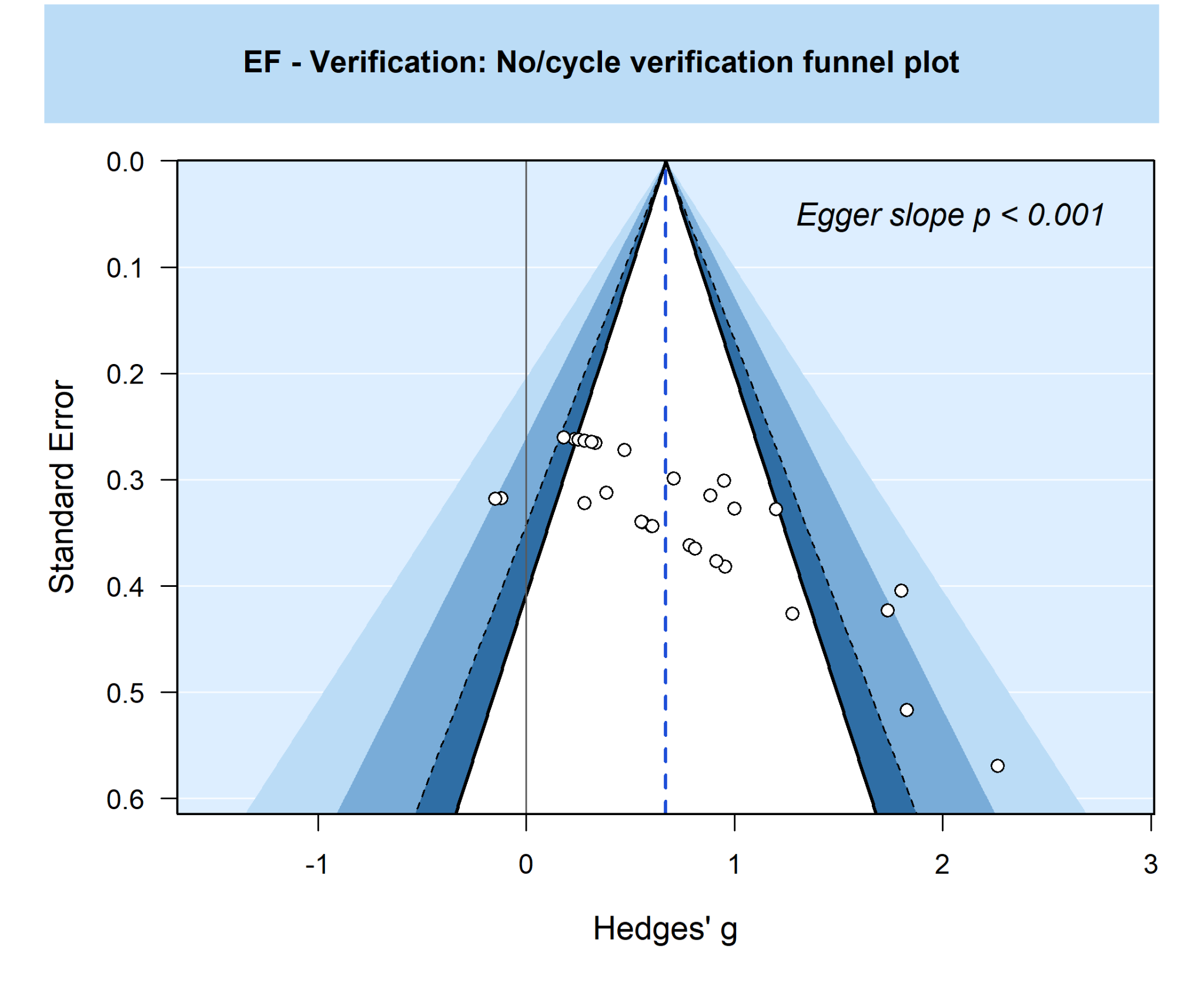 | **H**  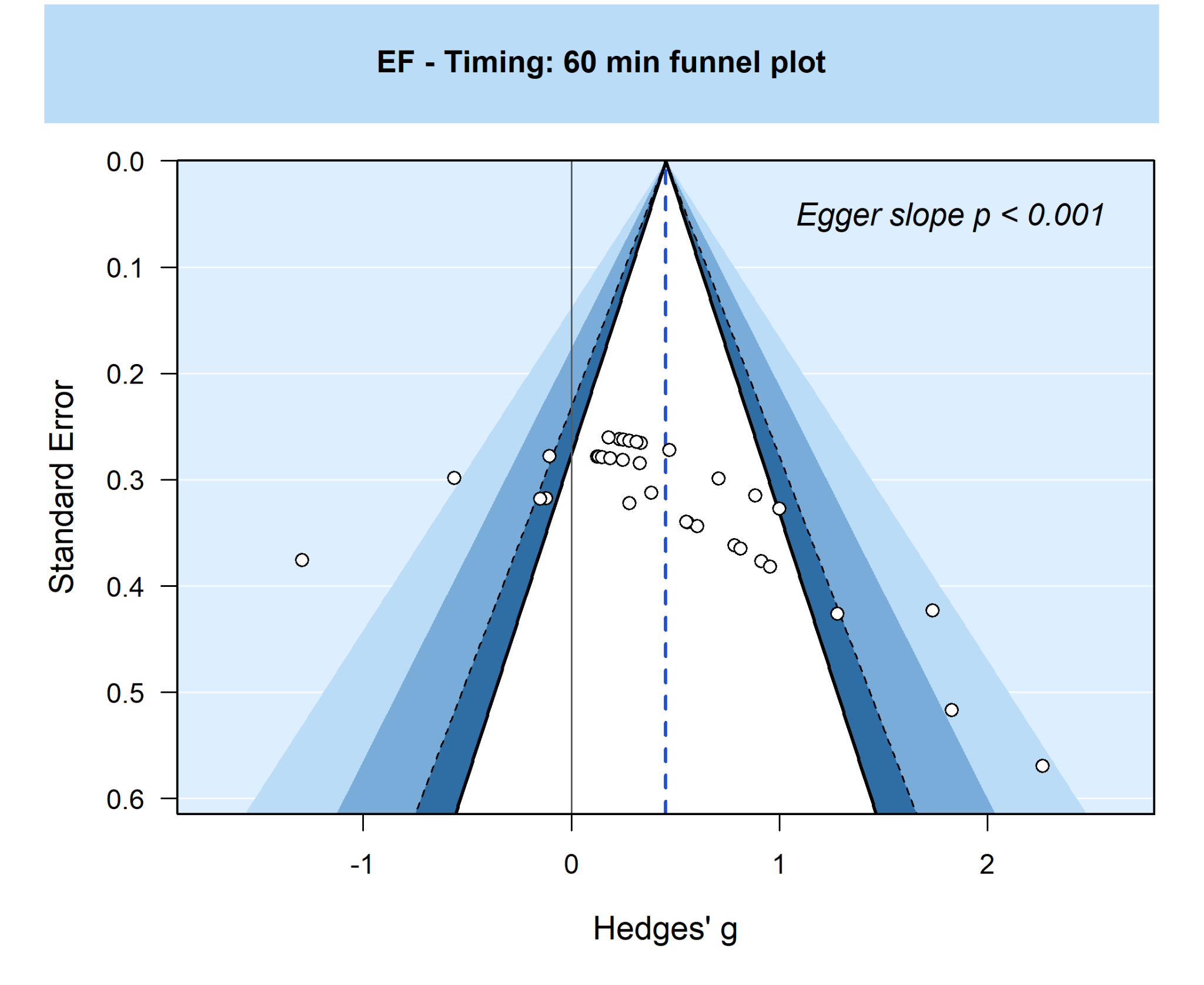 |
| **I**  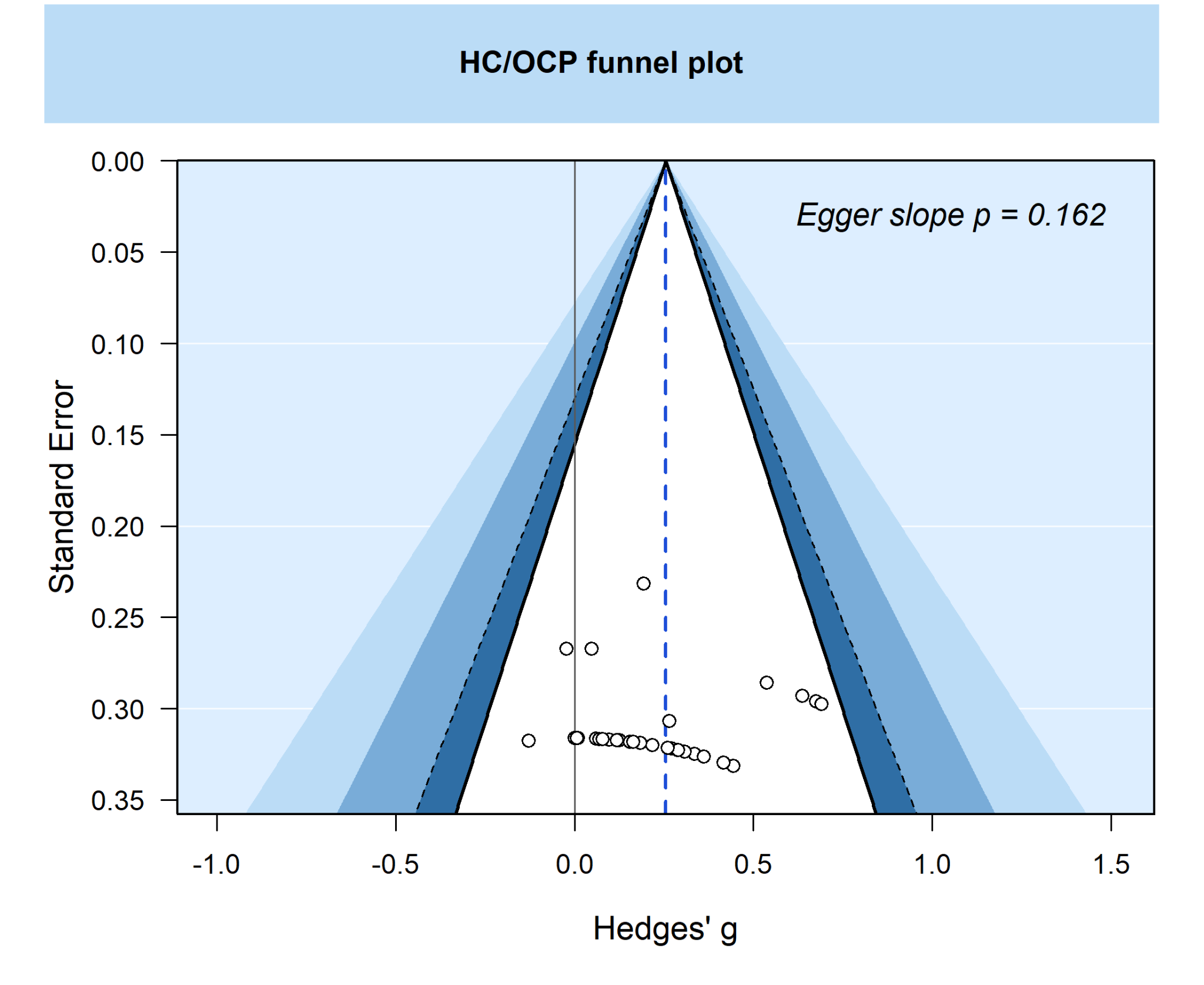 | **J**  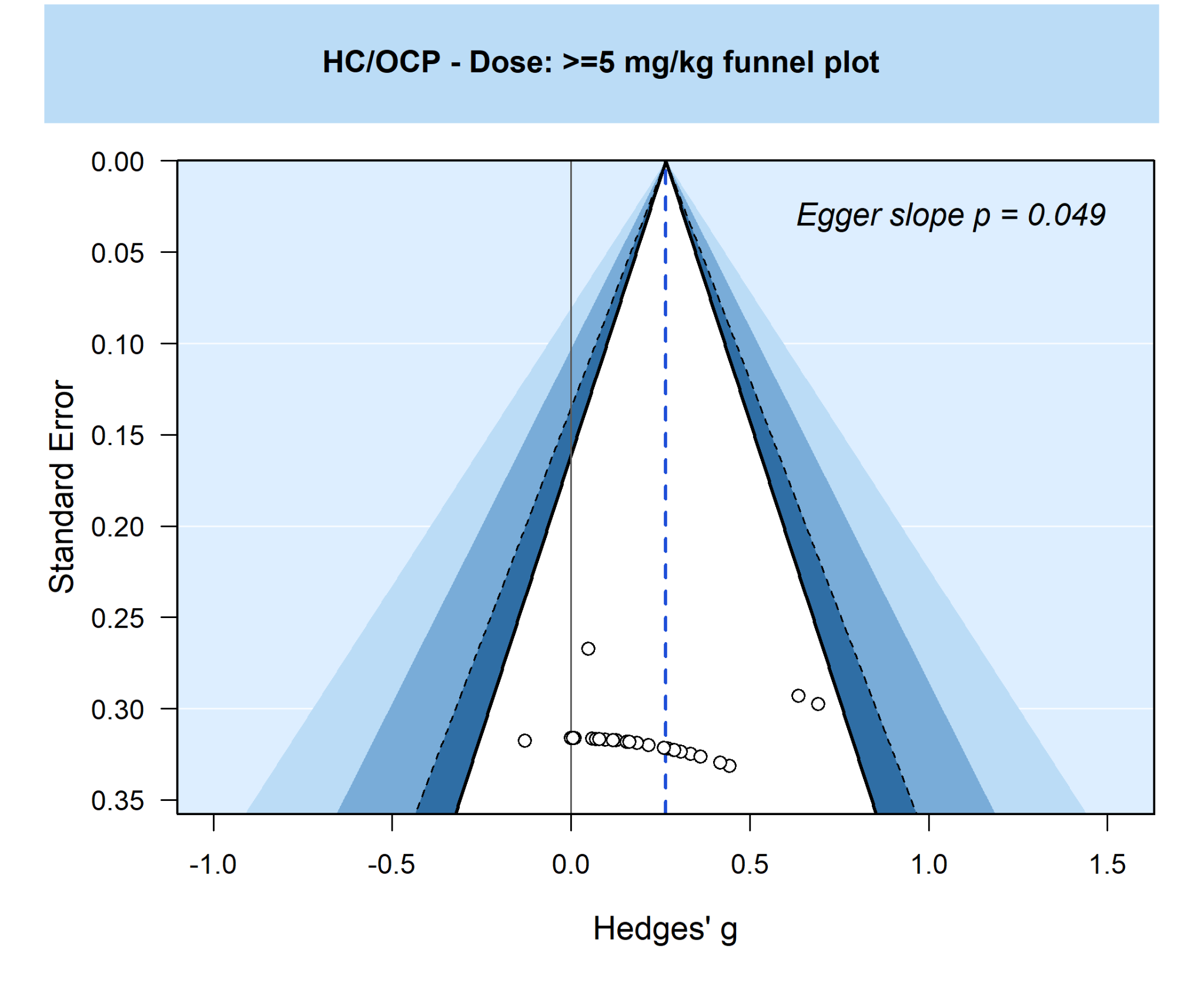 | **K**  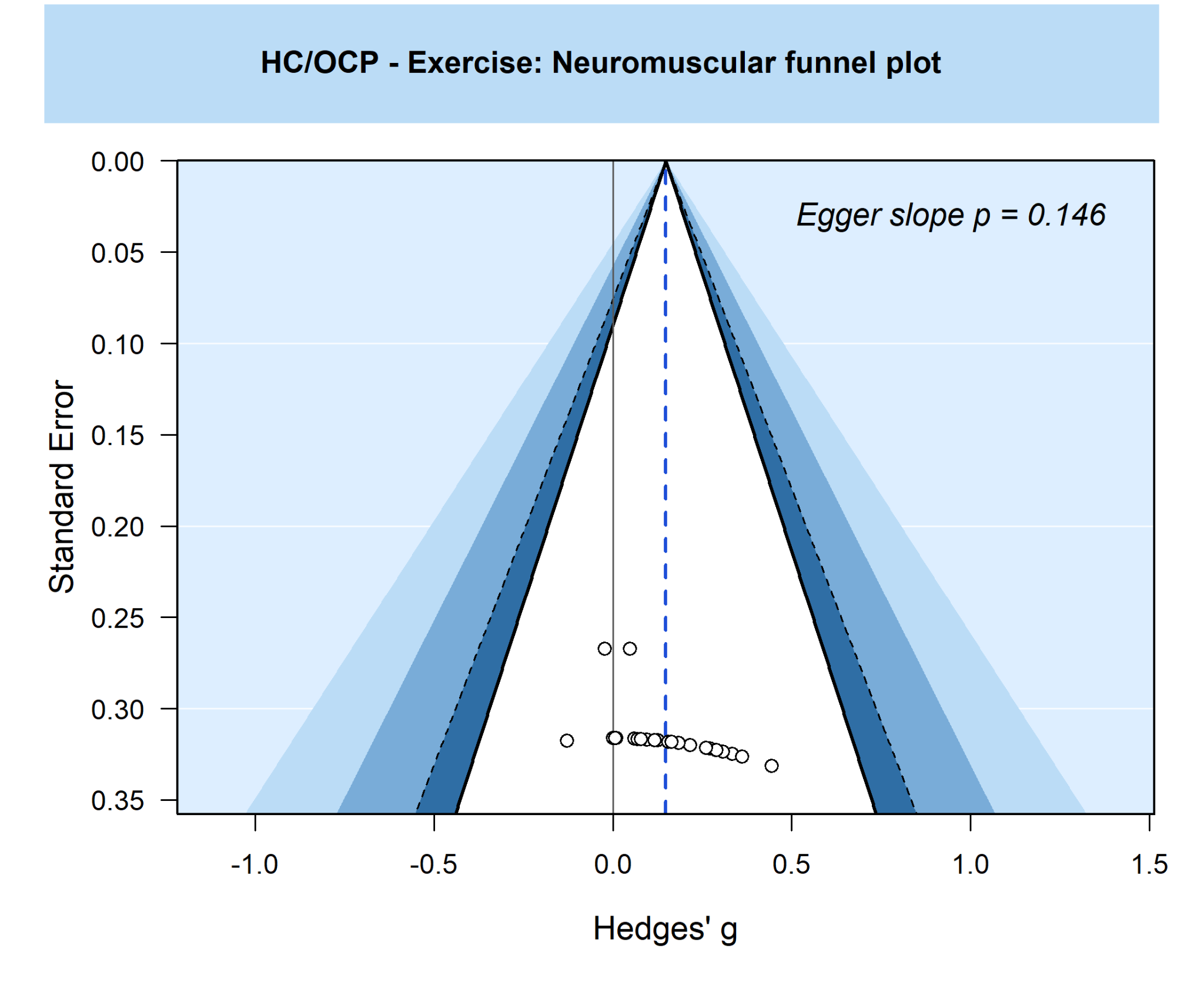 | **L**  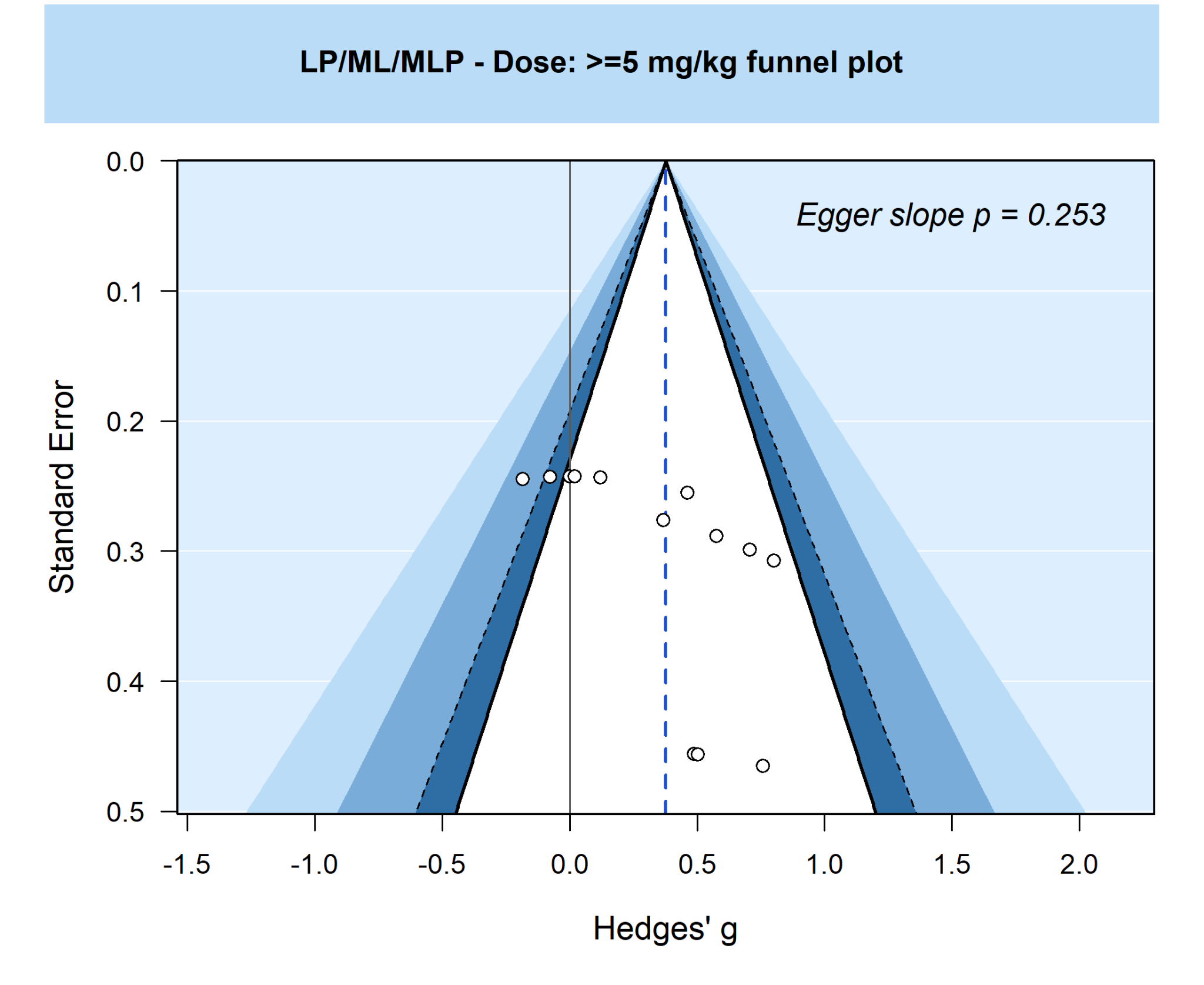 |
| **M**  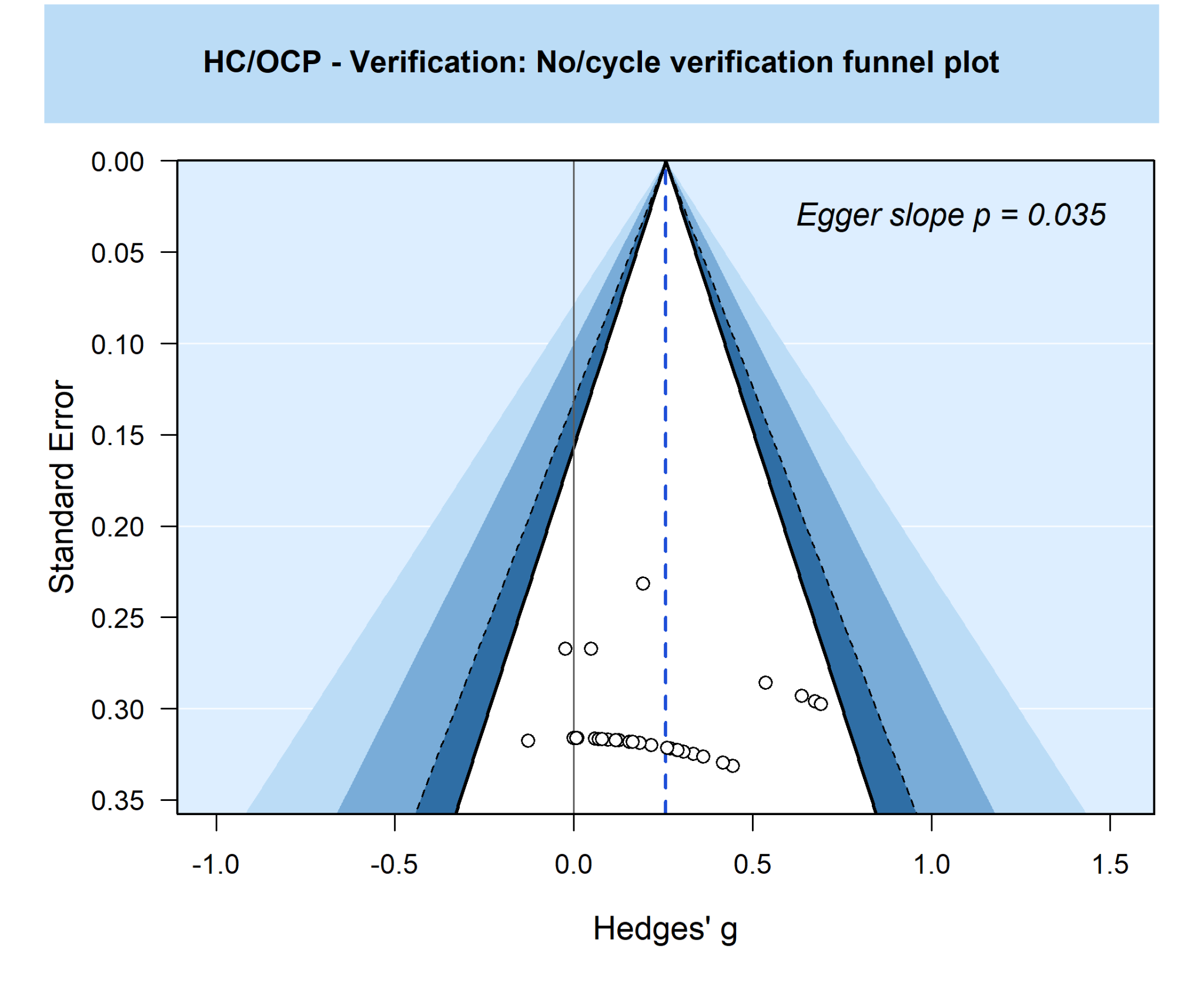 | **N**  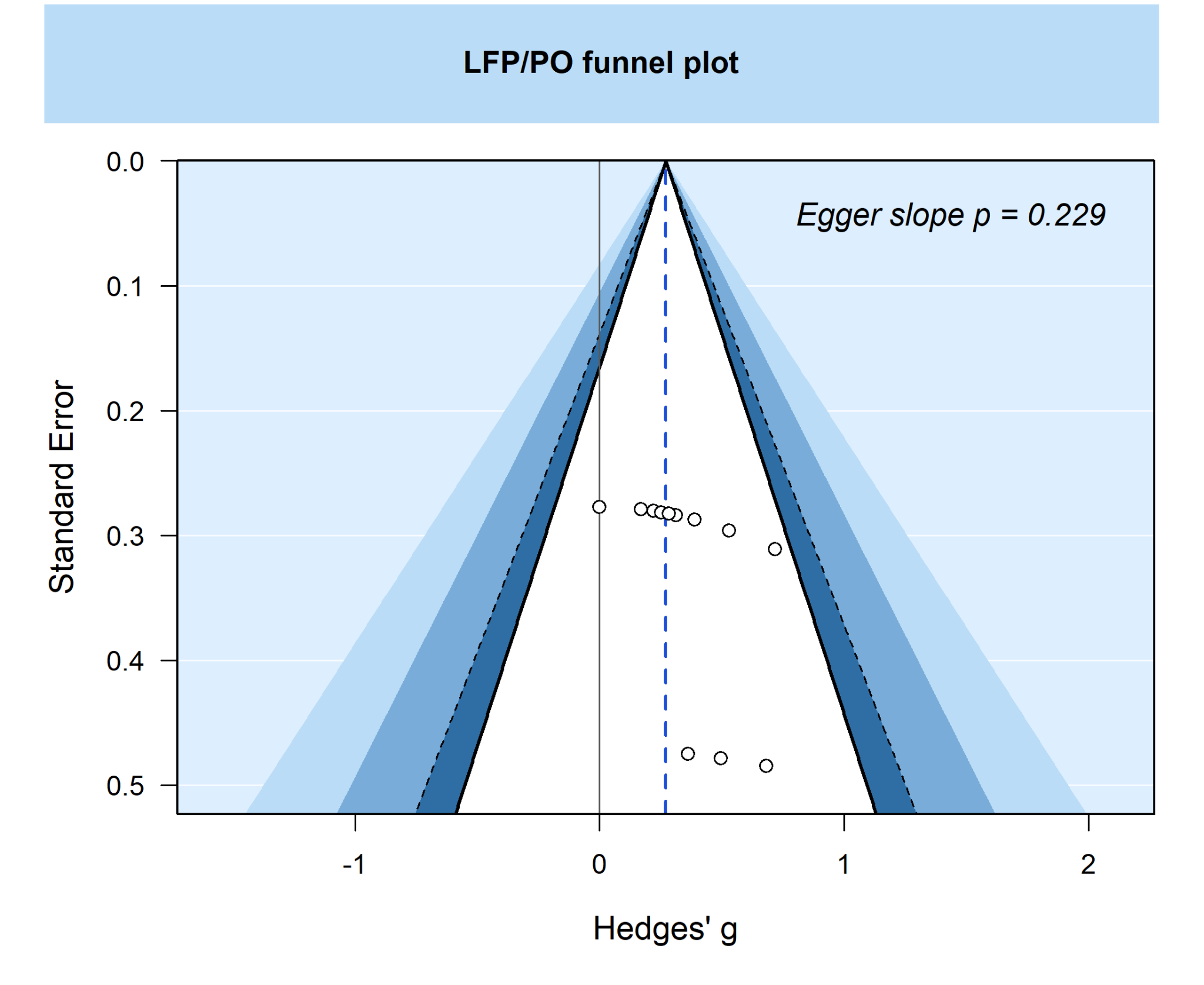 | **O**  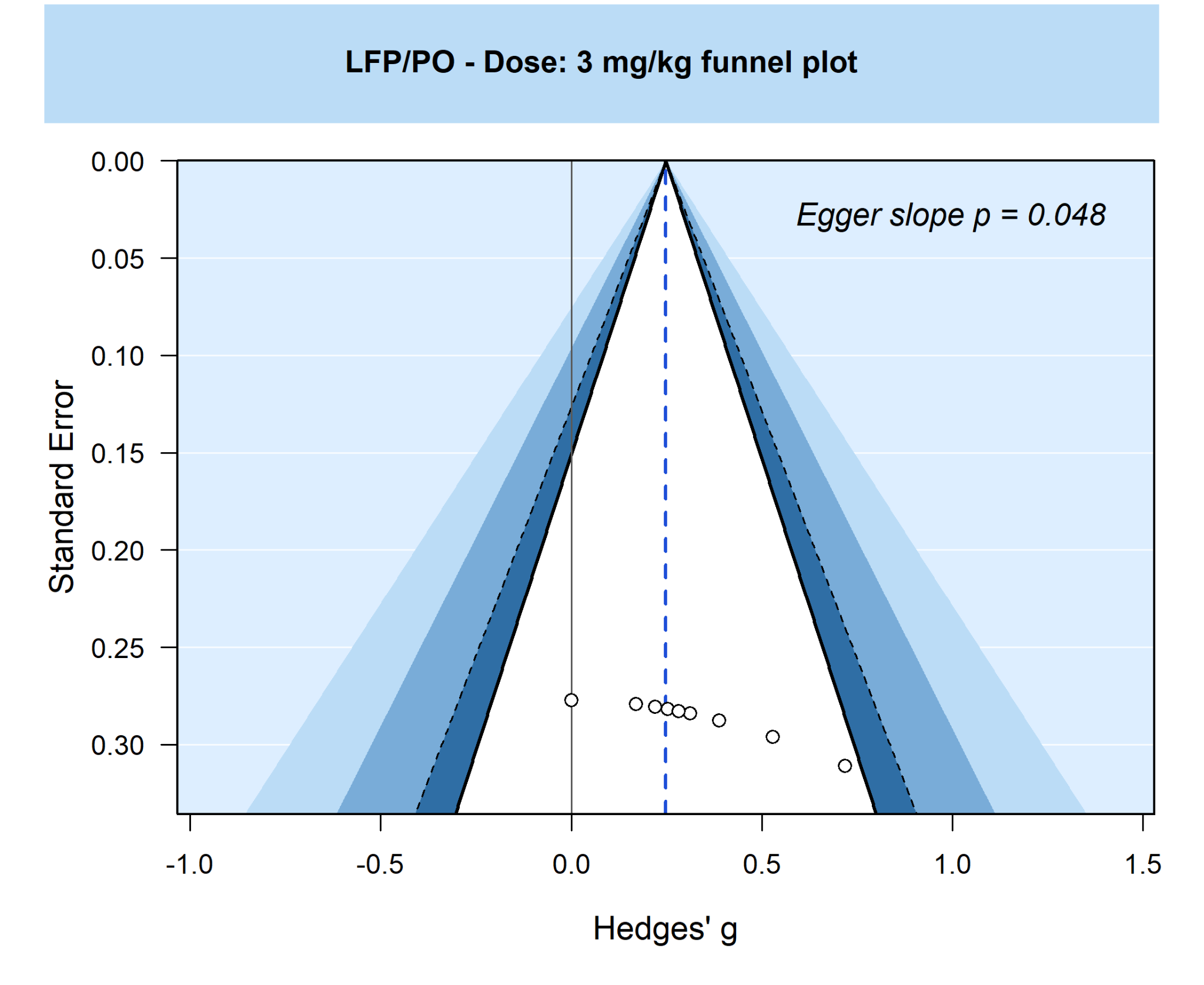 | **P**  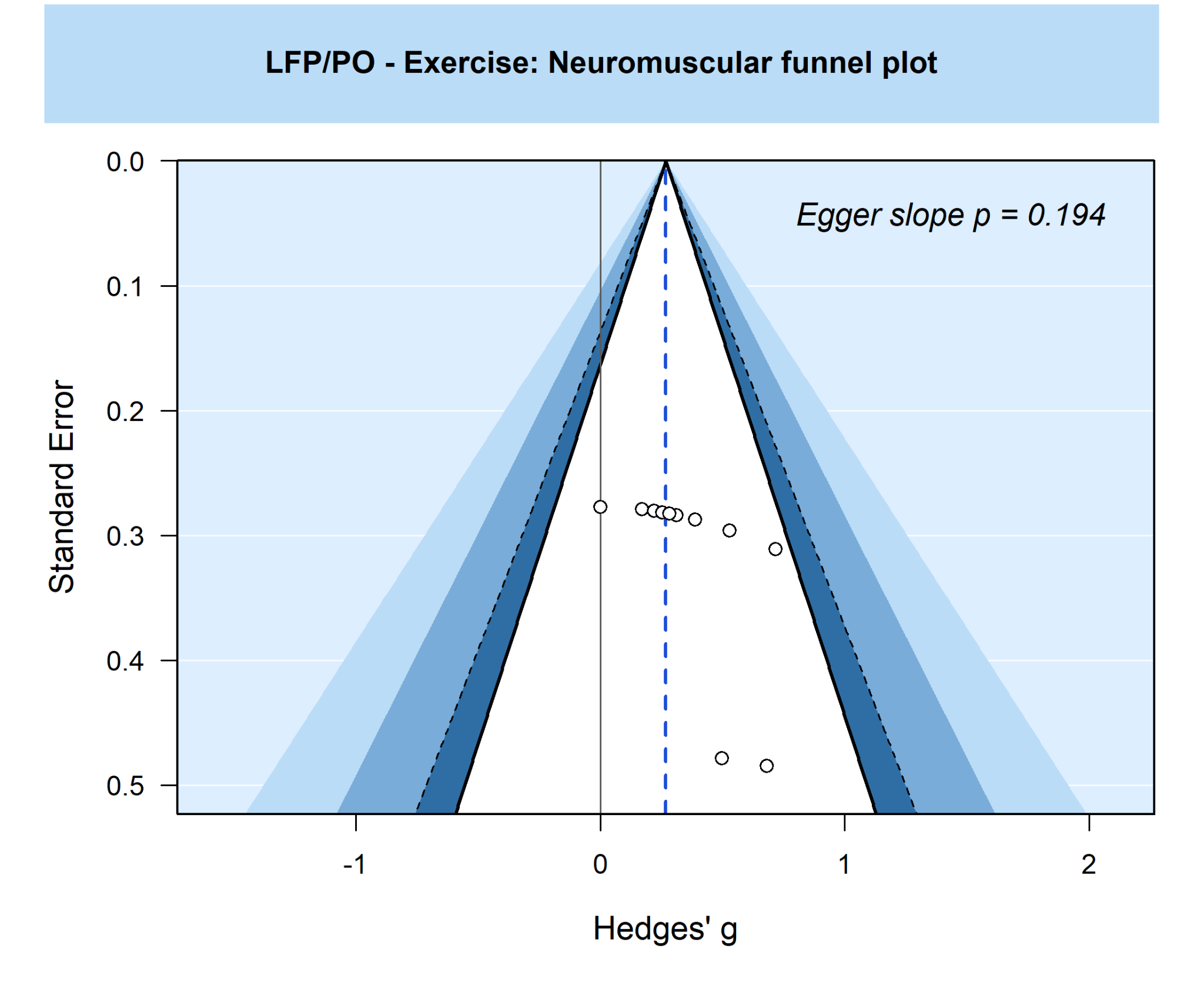 |
| **Q**  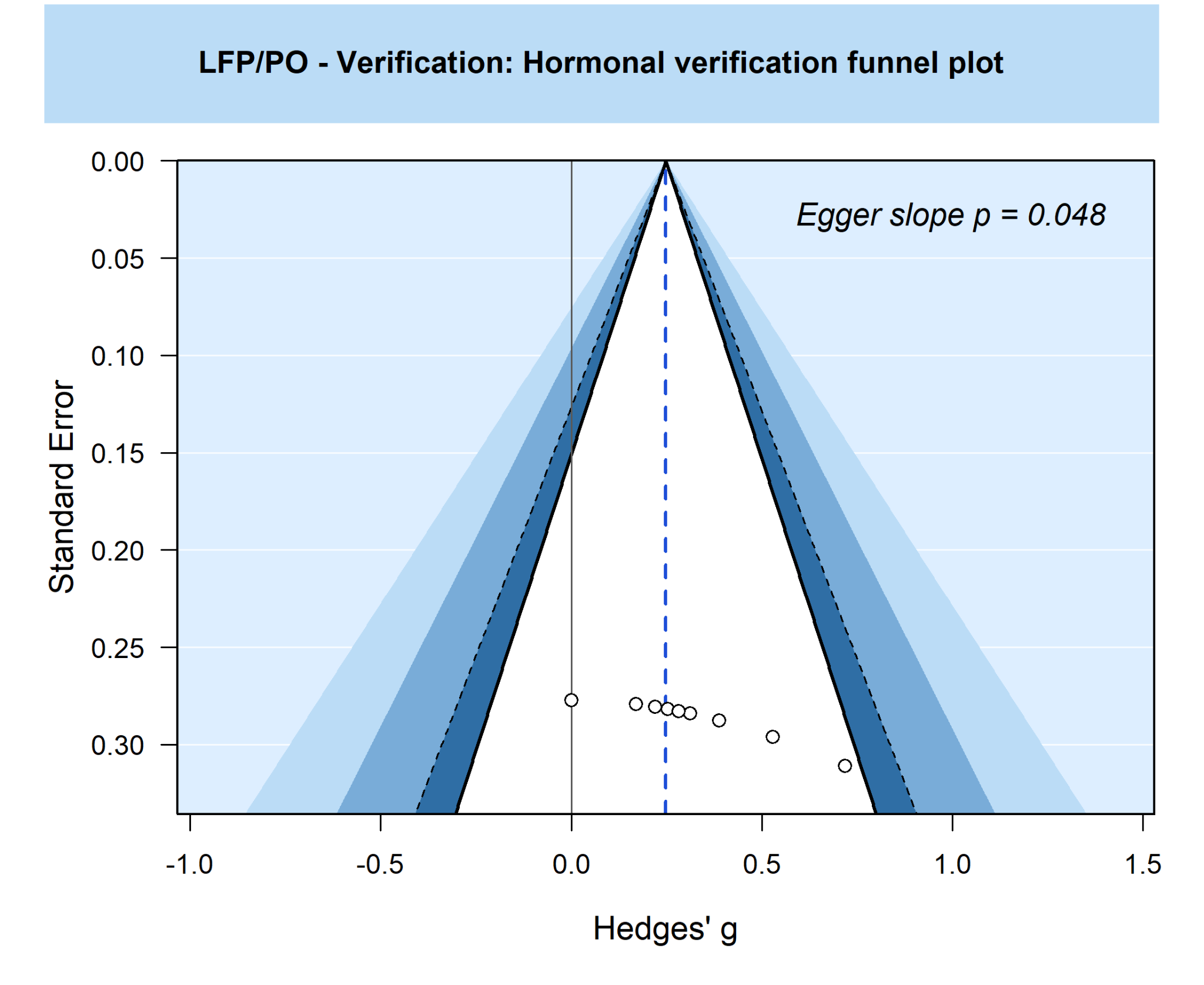 | **R**  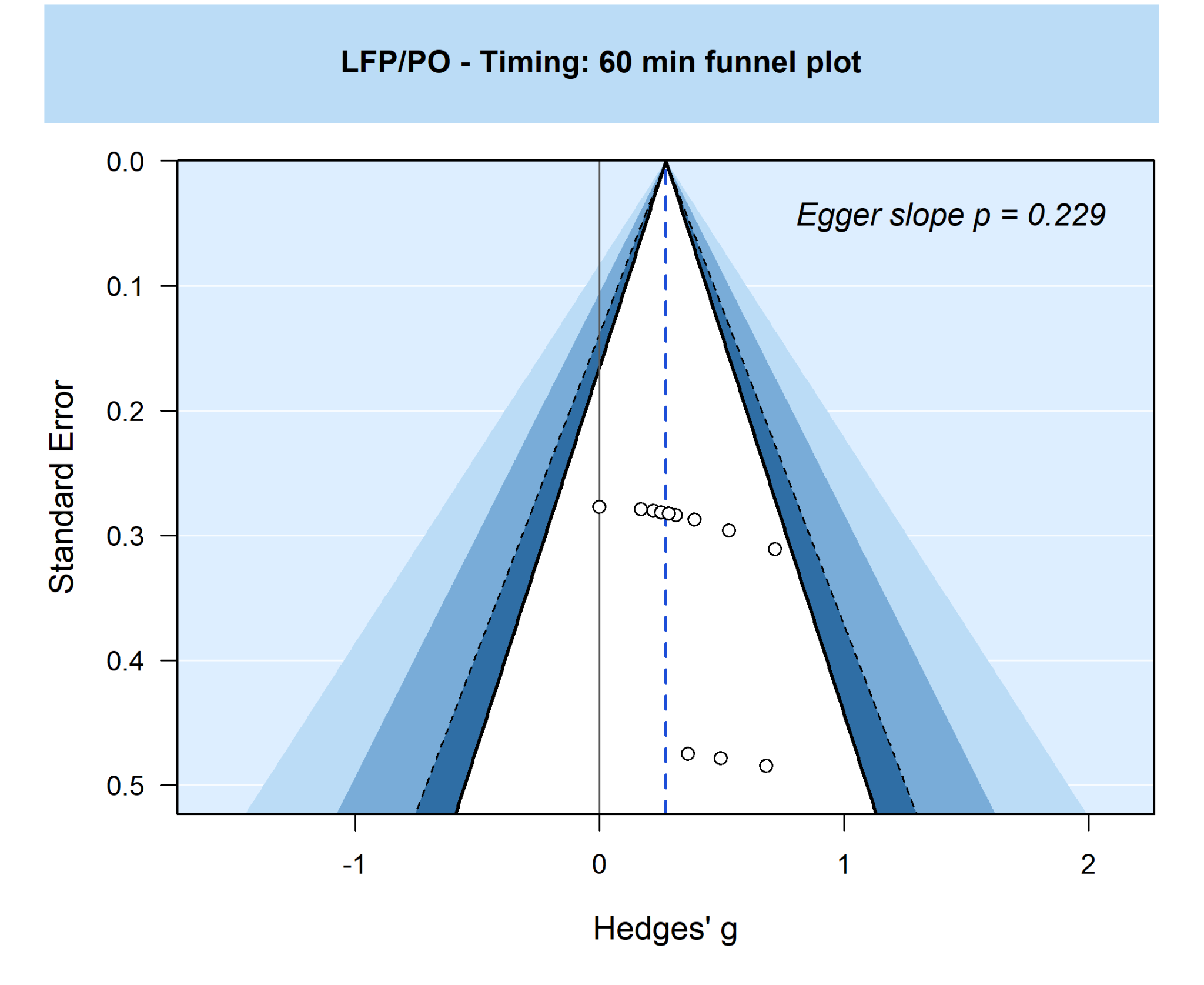 | **S**  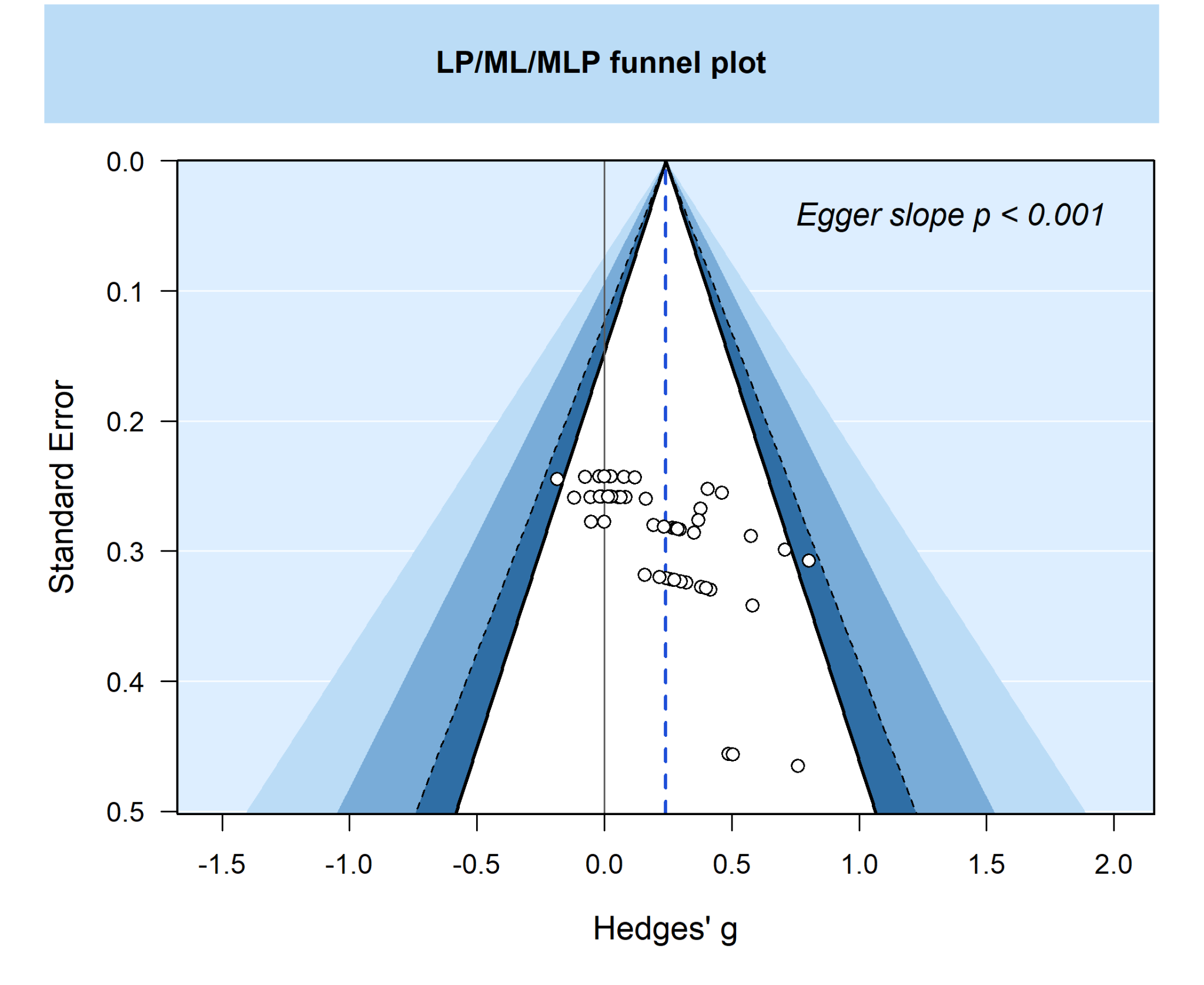 | **T**  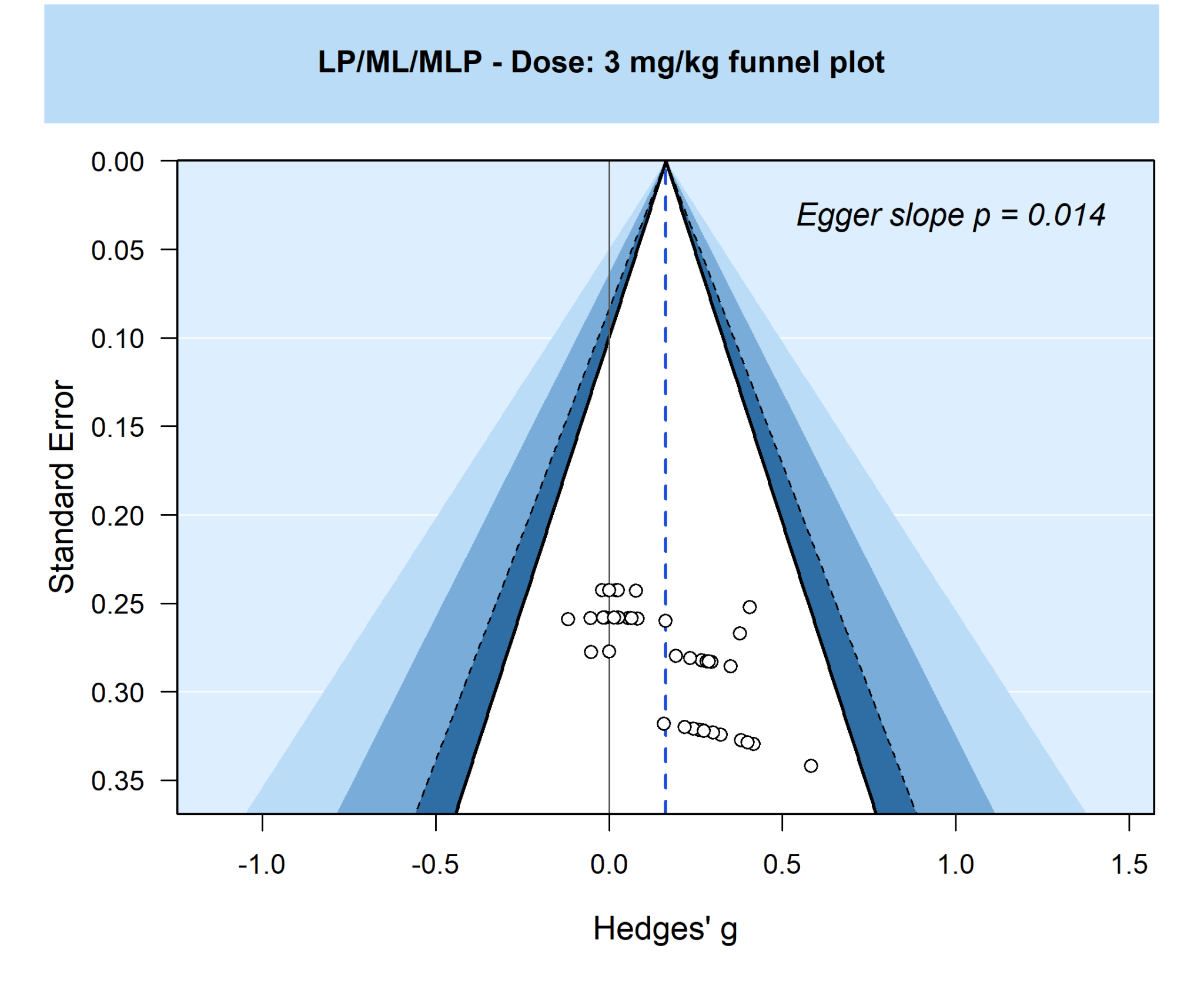 |
| **U**  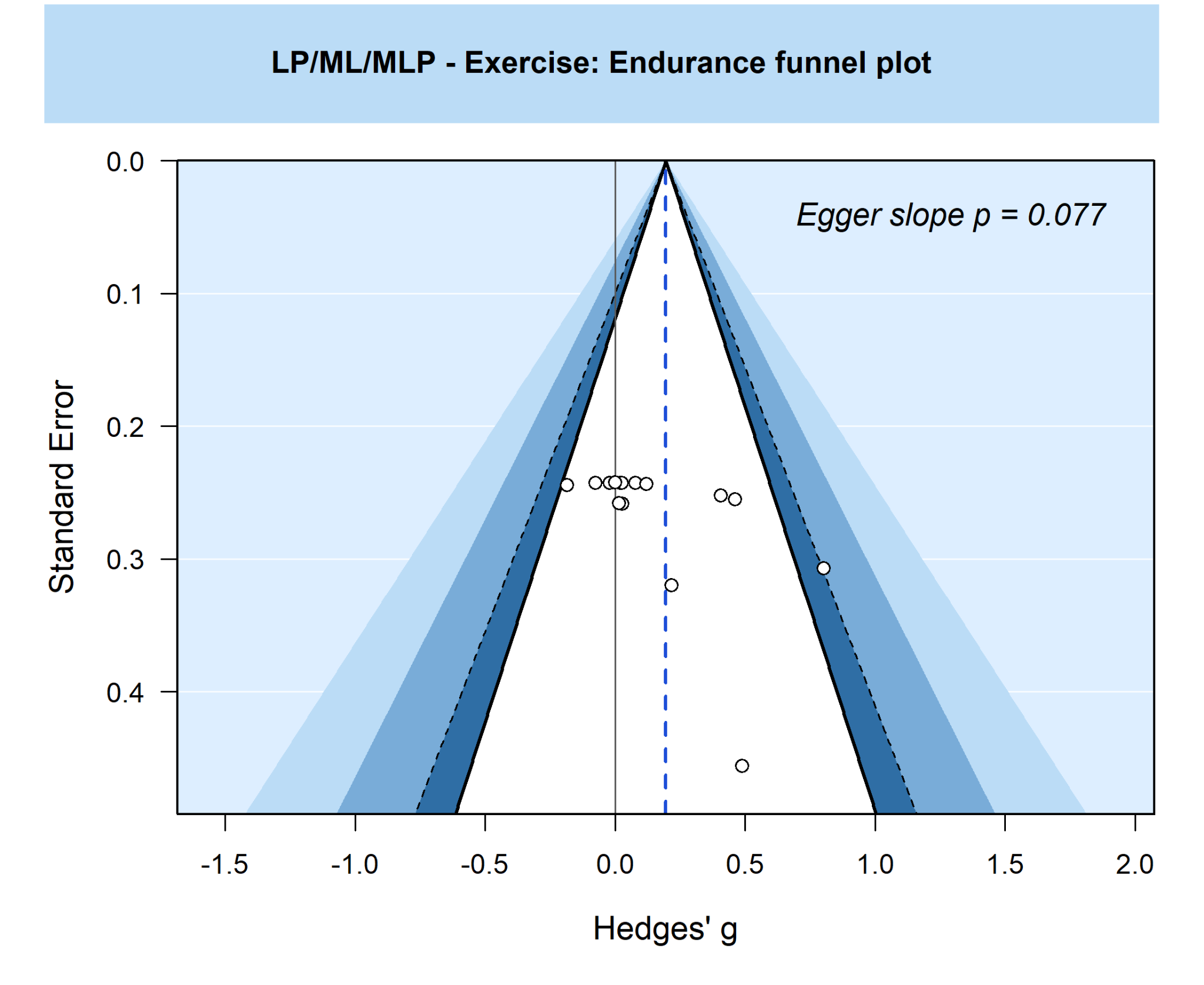 | **V**  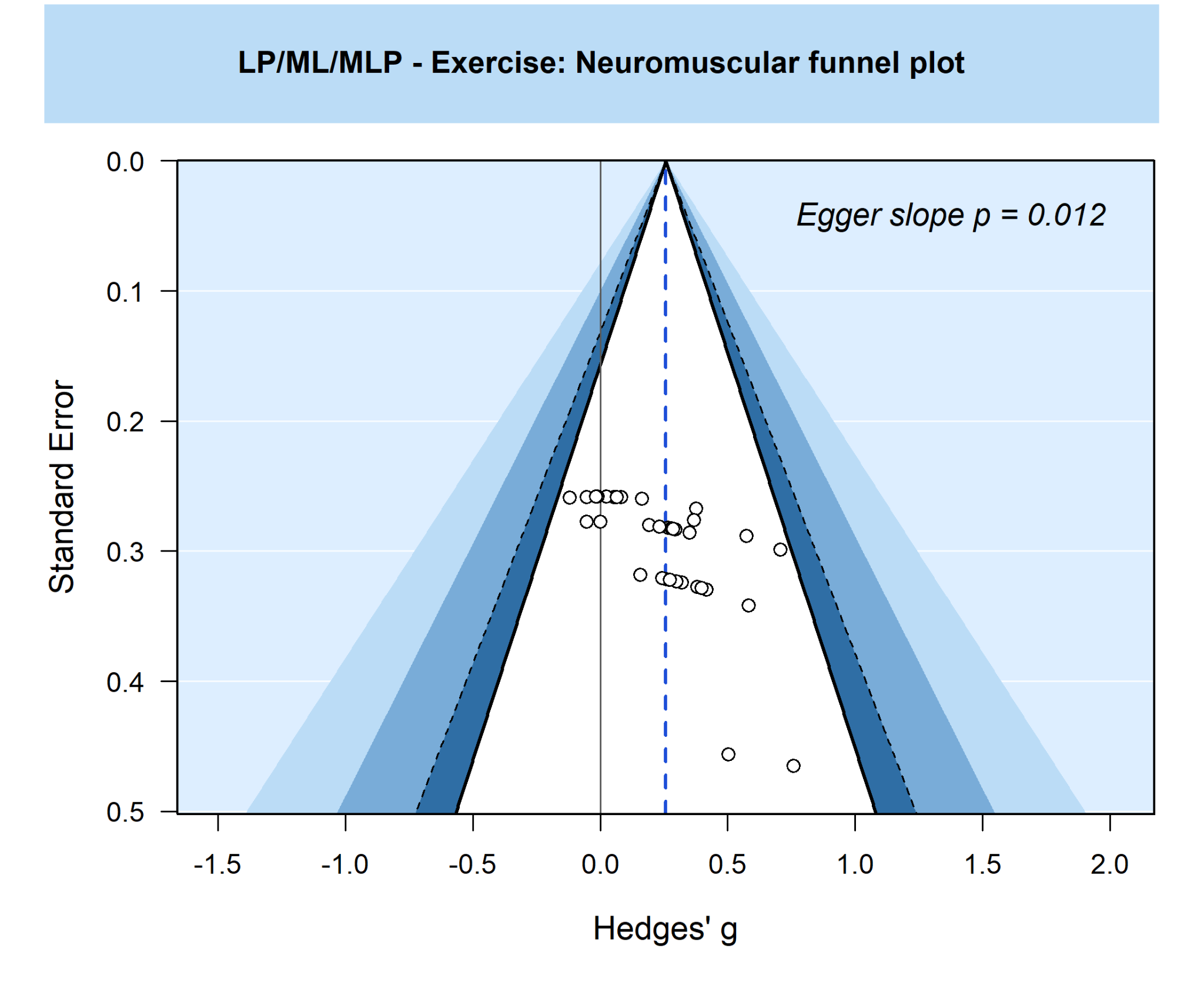 | **W**  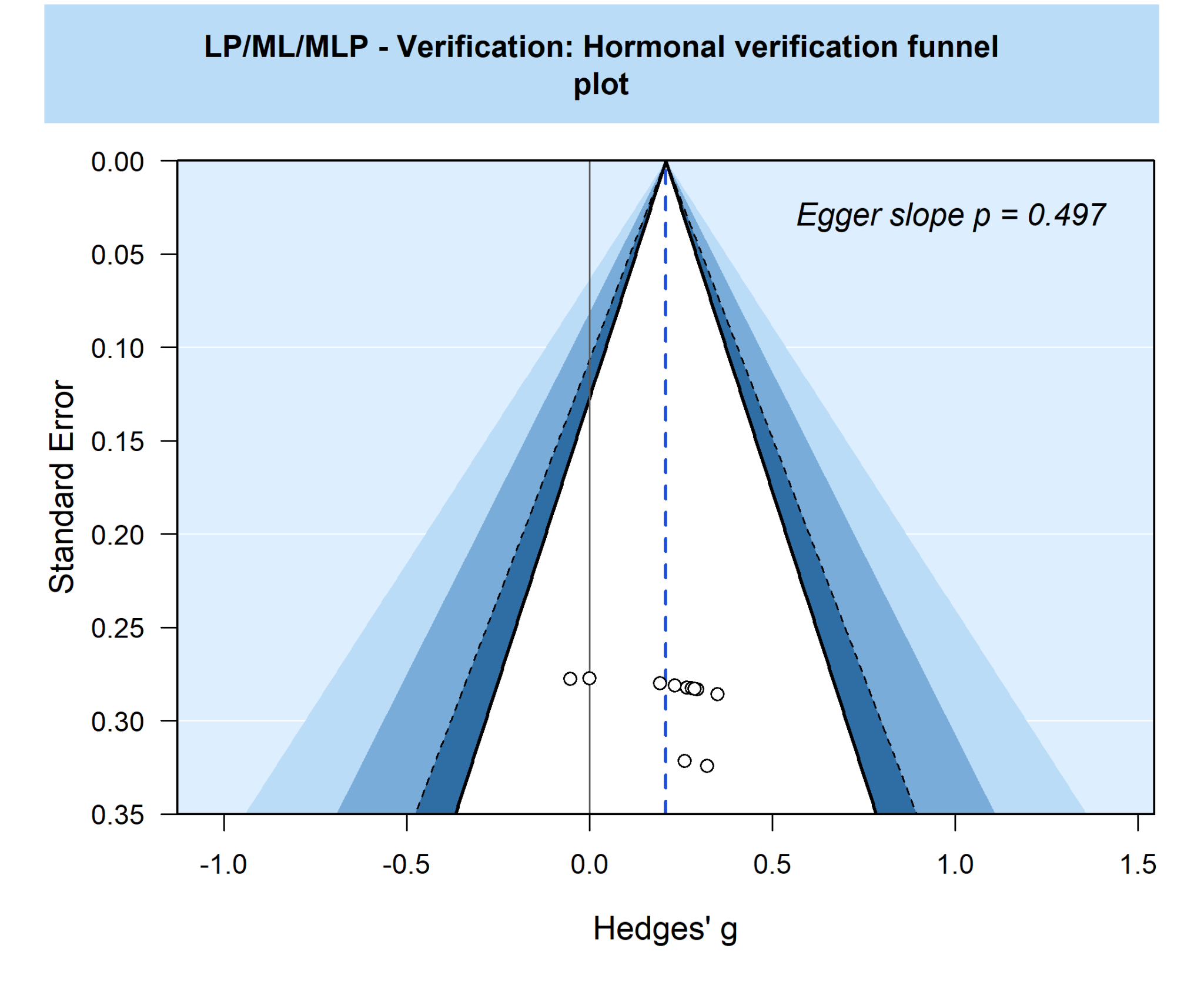 | **X**  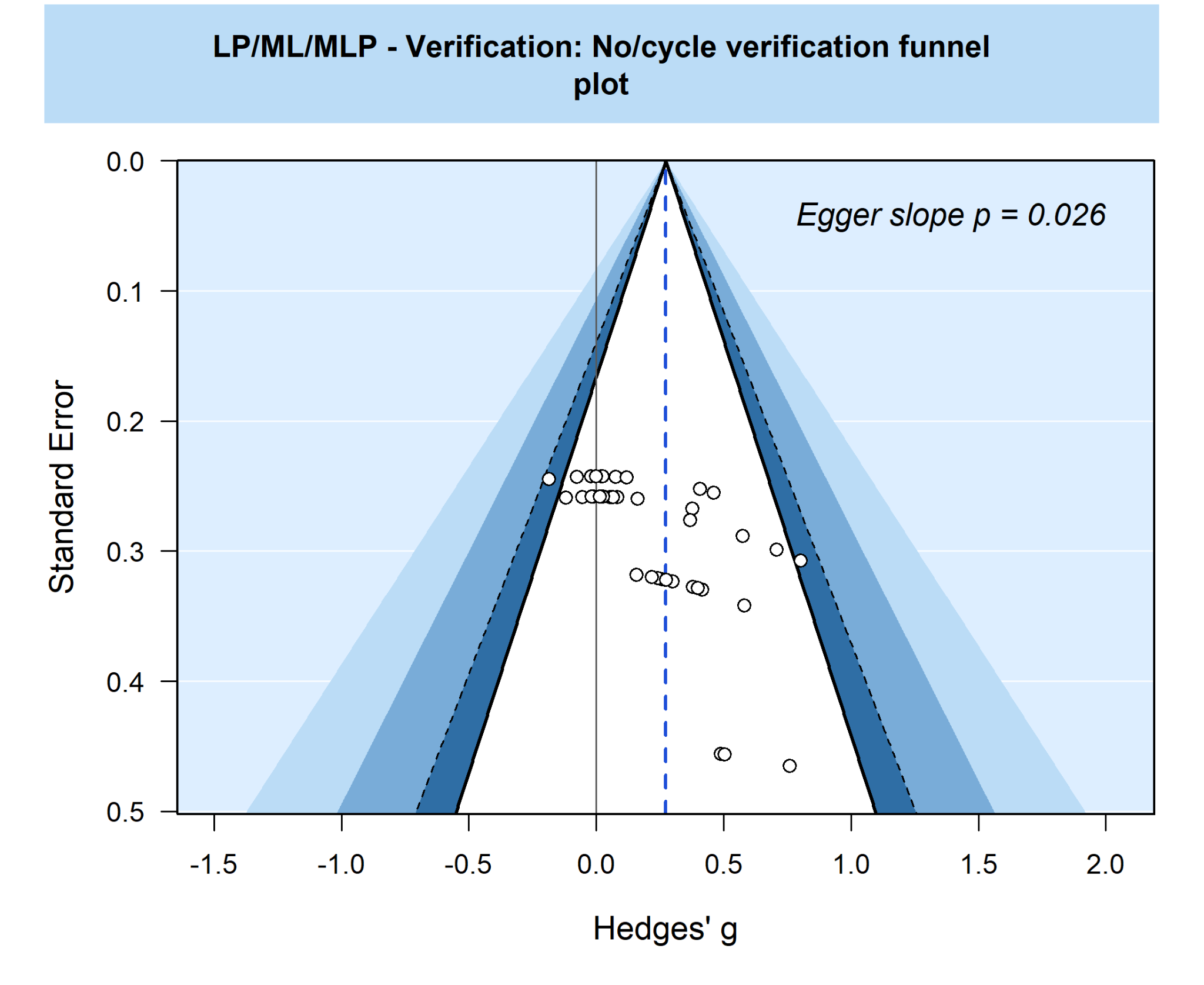 |
|  | **Y**  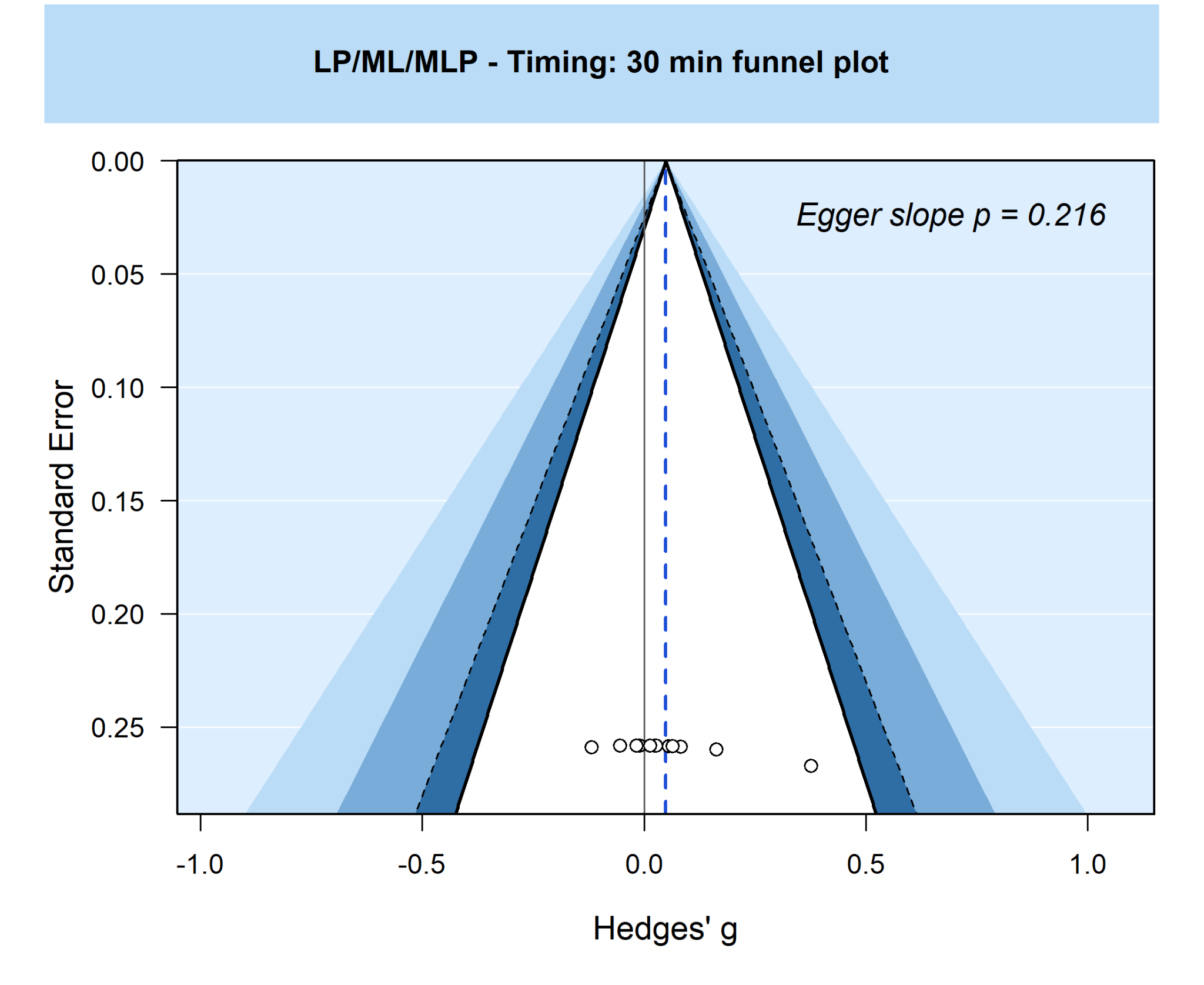 | **Z**  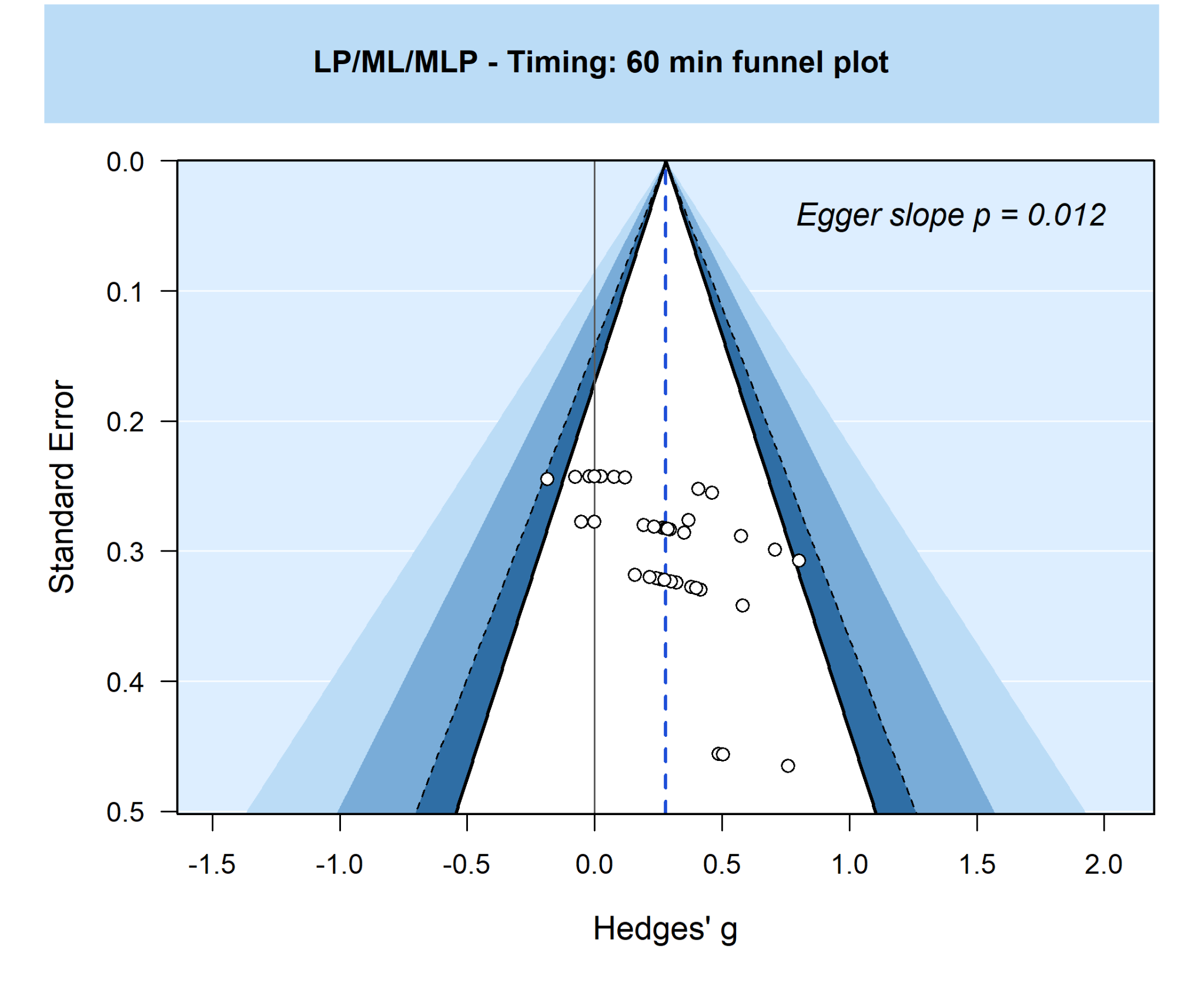 |  |

**Notes:** Open circles represent individual effect sizes. Panels A–H show EF analyses: overall, 3 mg/kg, 4 mg/kg, endurance, neuromuscular, hormonal verification, no/cycle verification, and 60 min. Panels I–M show HC/OCP analyses: overall, ≥5 mg/kg, neuromuscular, no/cycle verification, and 60 min. Panels N–R show LFP/PO analyses: overall, 3 mg/kg, neuromuscular, hormonal verification, and 60 min. Panels S–Z show LP/ML/MLP analyses: overall, 3 mg/kg, endurance, neuromuscular, hormonal verification, no/cycle verification, 30 min, and 60 min. Abbreviations: EF, early follicular phase; LFP/PO, late follicular/peri-ovulatory phase; LP/ML/MLP, luteal/mid-luteal/mid-luteal phase; HC/OCP, hormonal contraceptive/oral contraceptive users.

**Electronic Supplementary Material Appendix S10 (Sunset funnel plots for statistical power visualization in primary, subgroup, and exploratory analyses)
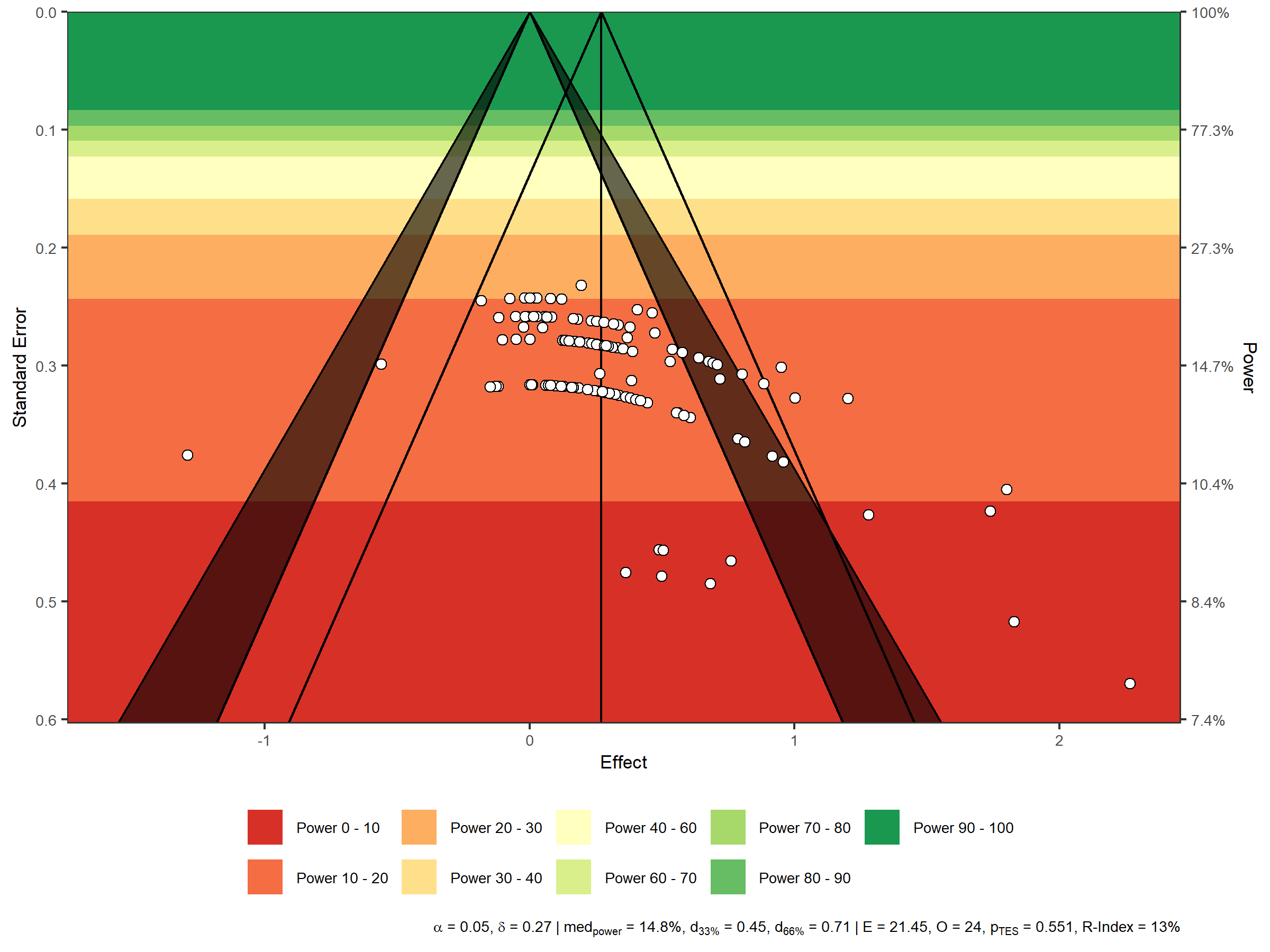
**

| **A**  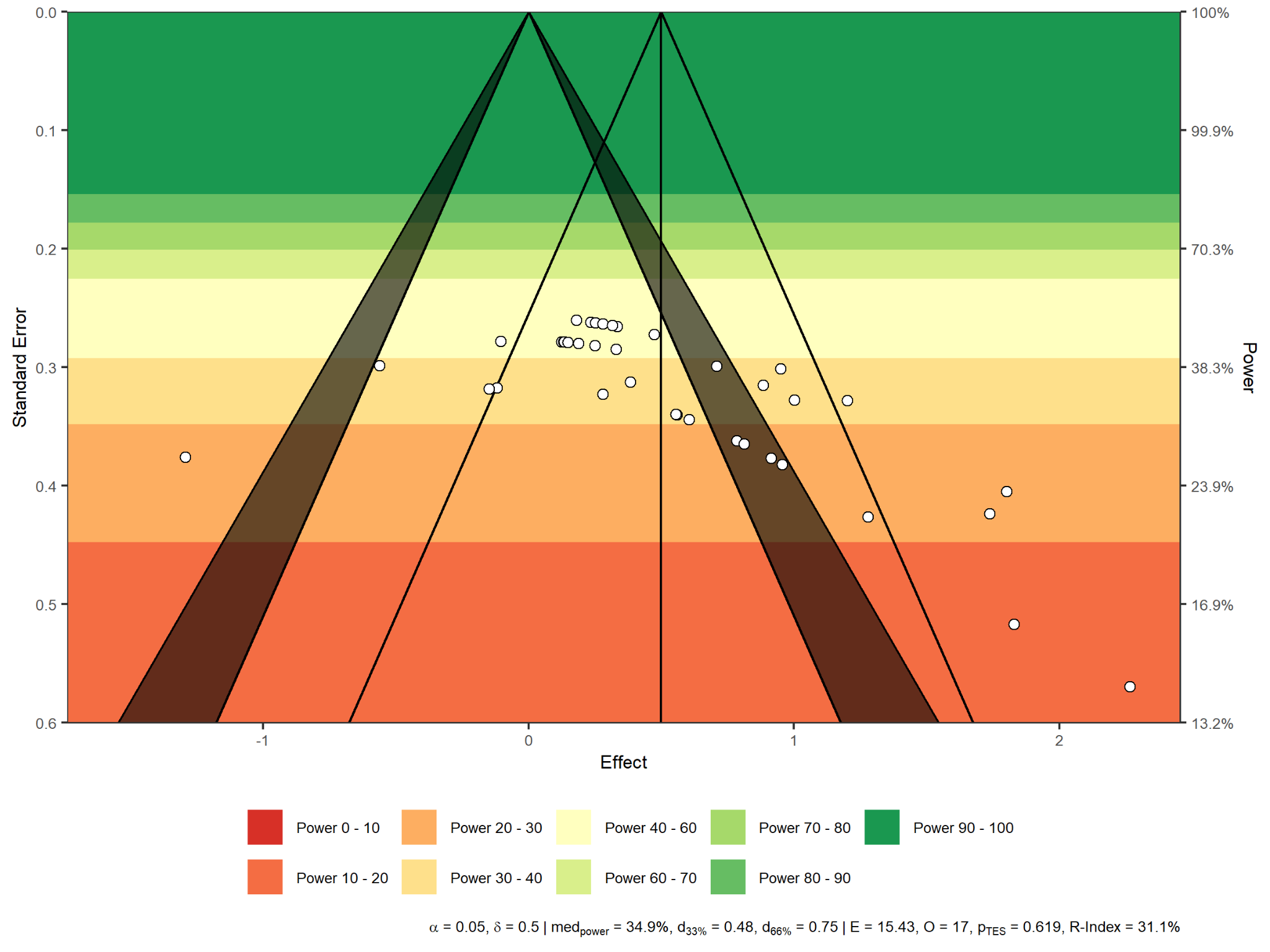 | **B**  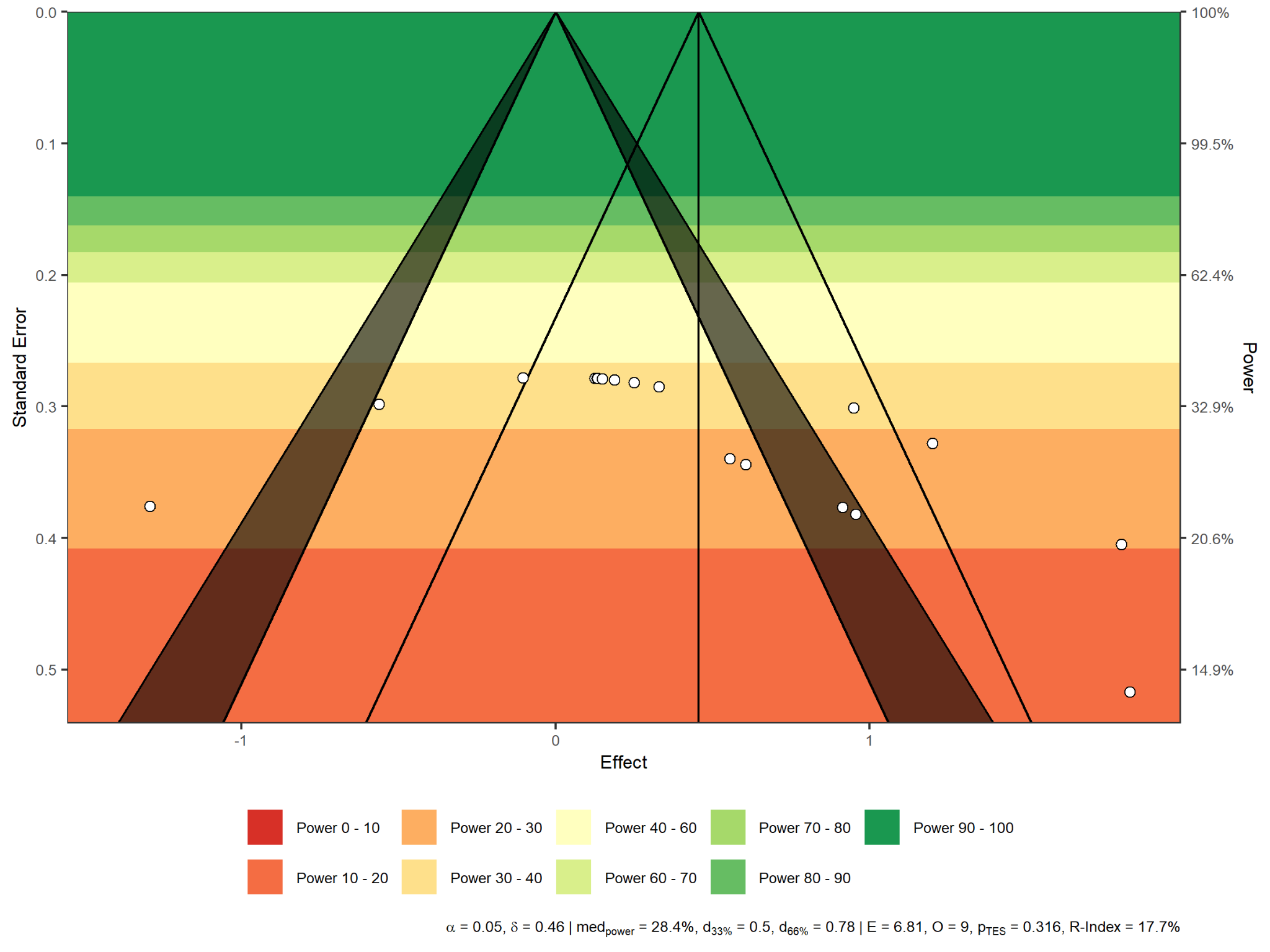 | **C**  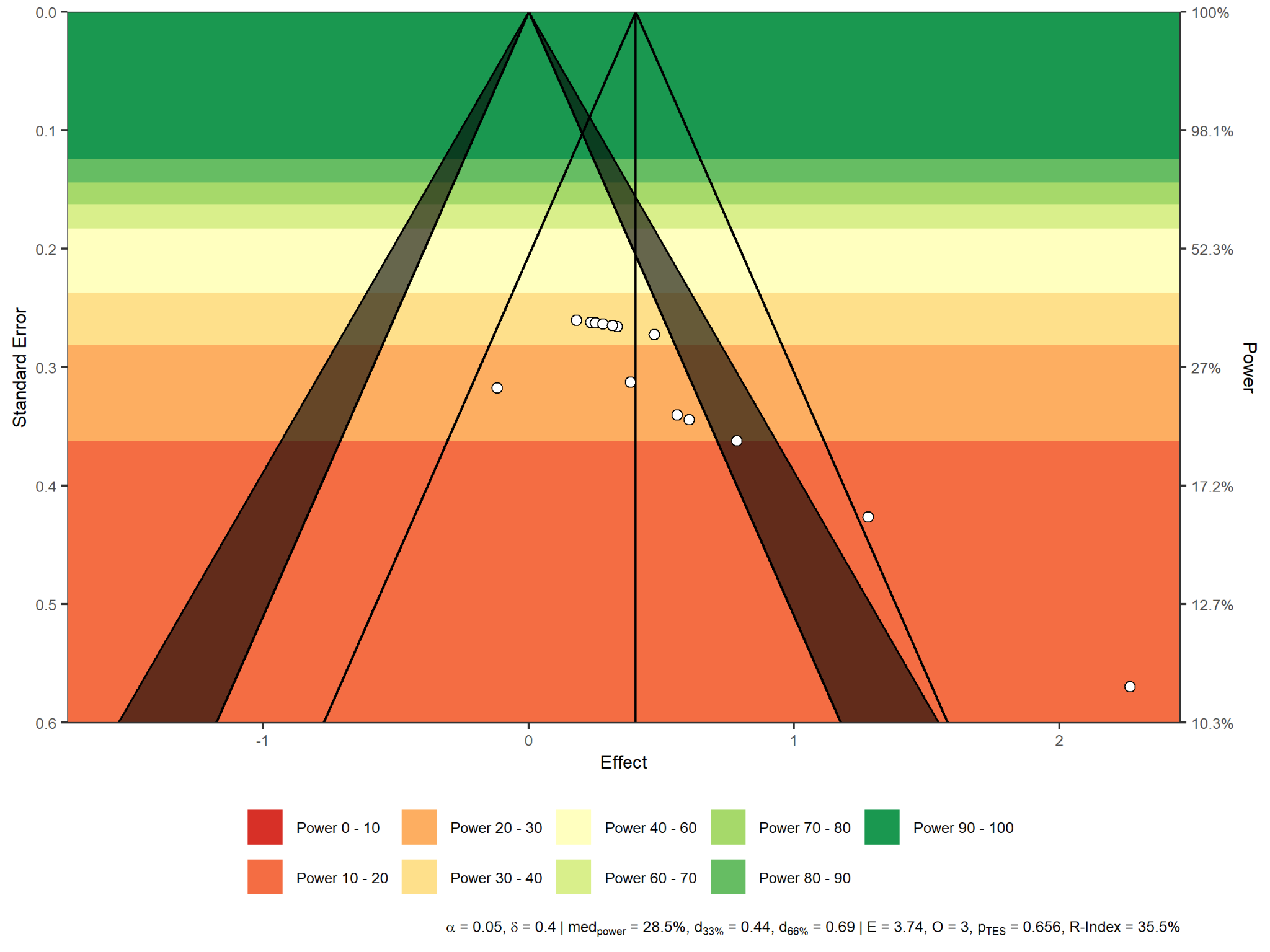 | **D**  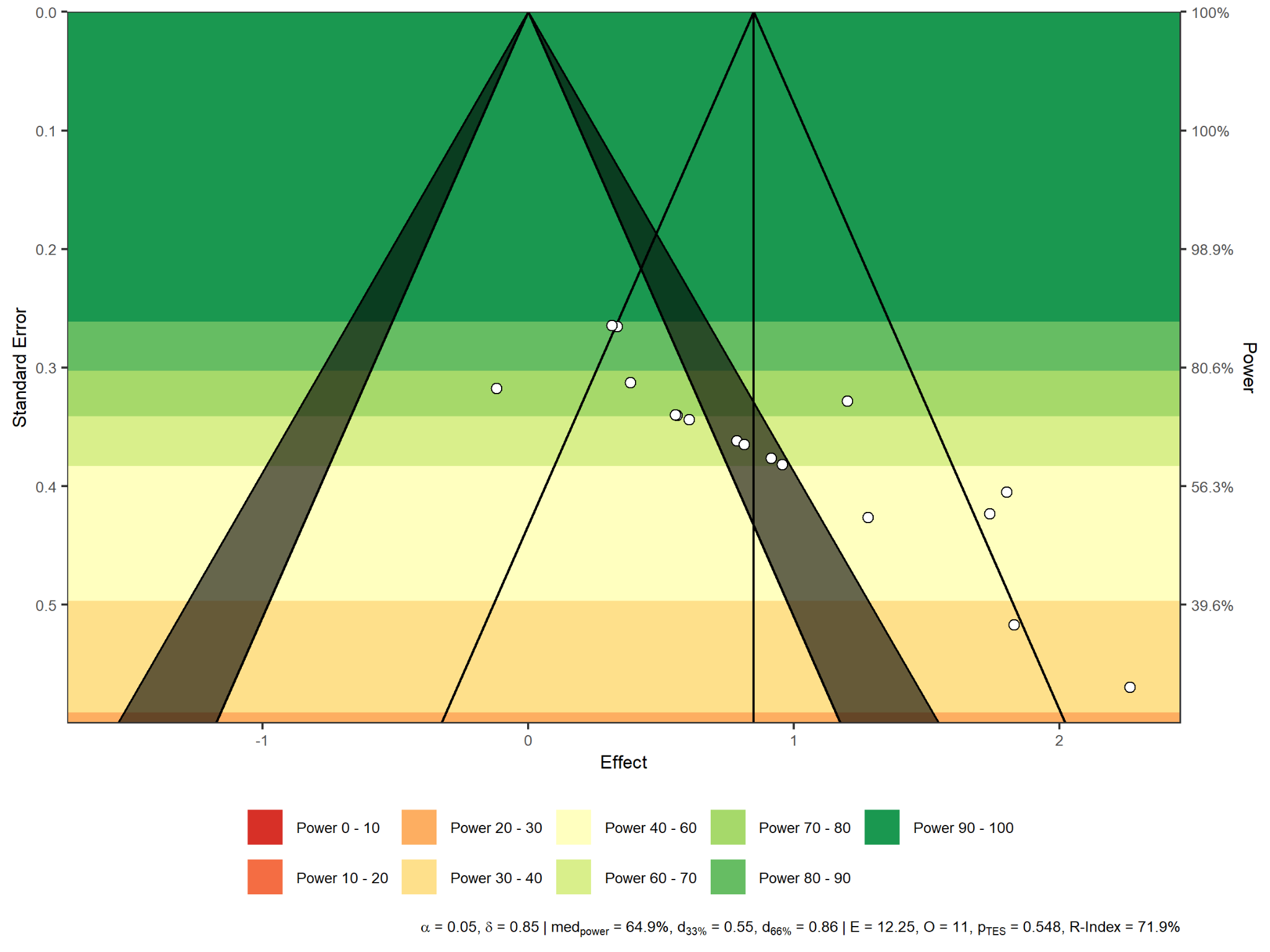 |
| --- | --- | --- | --- |
| **E**  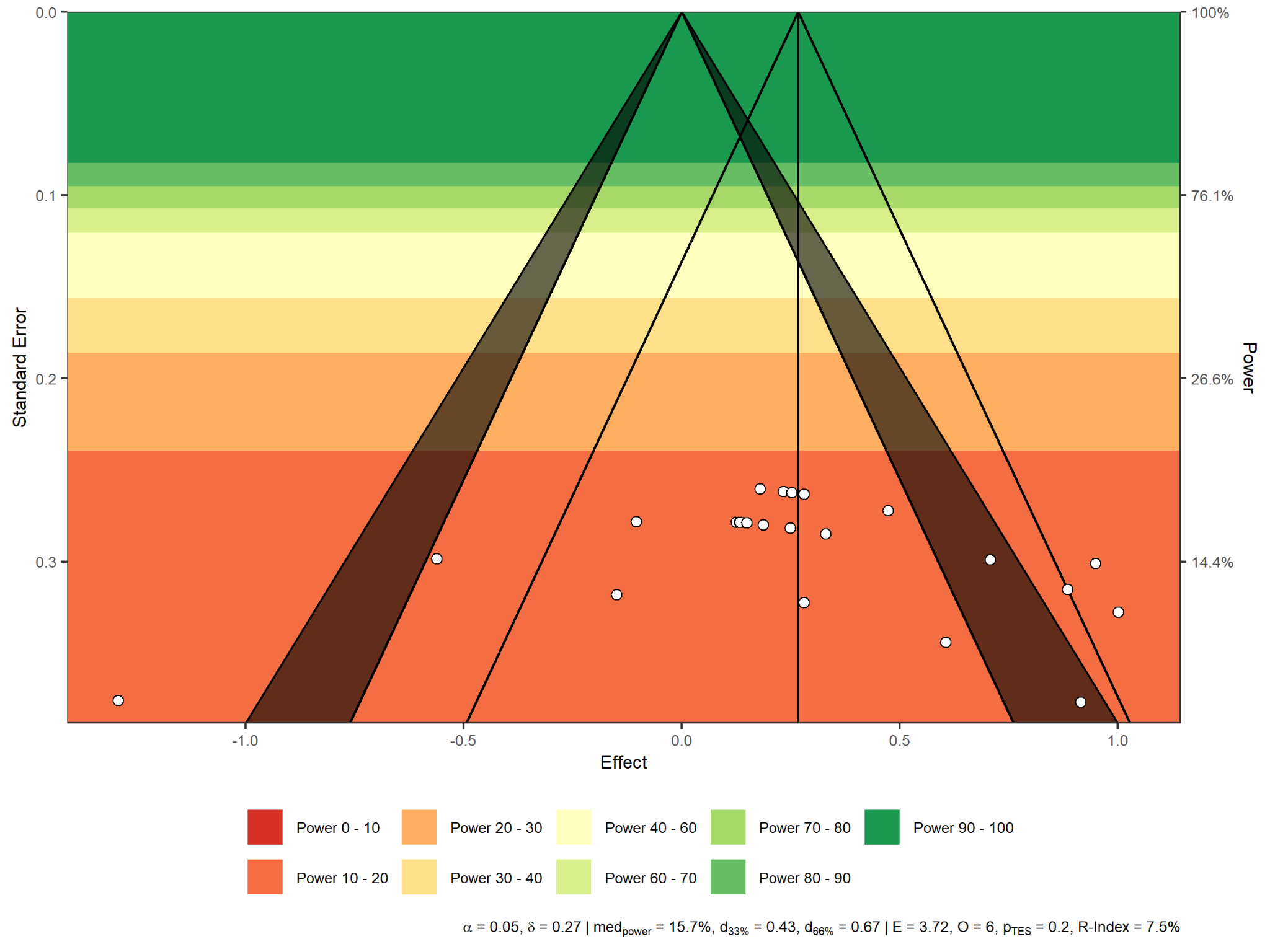 | **F**  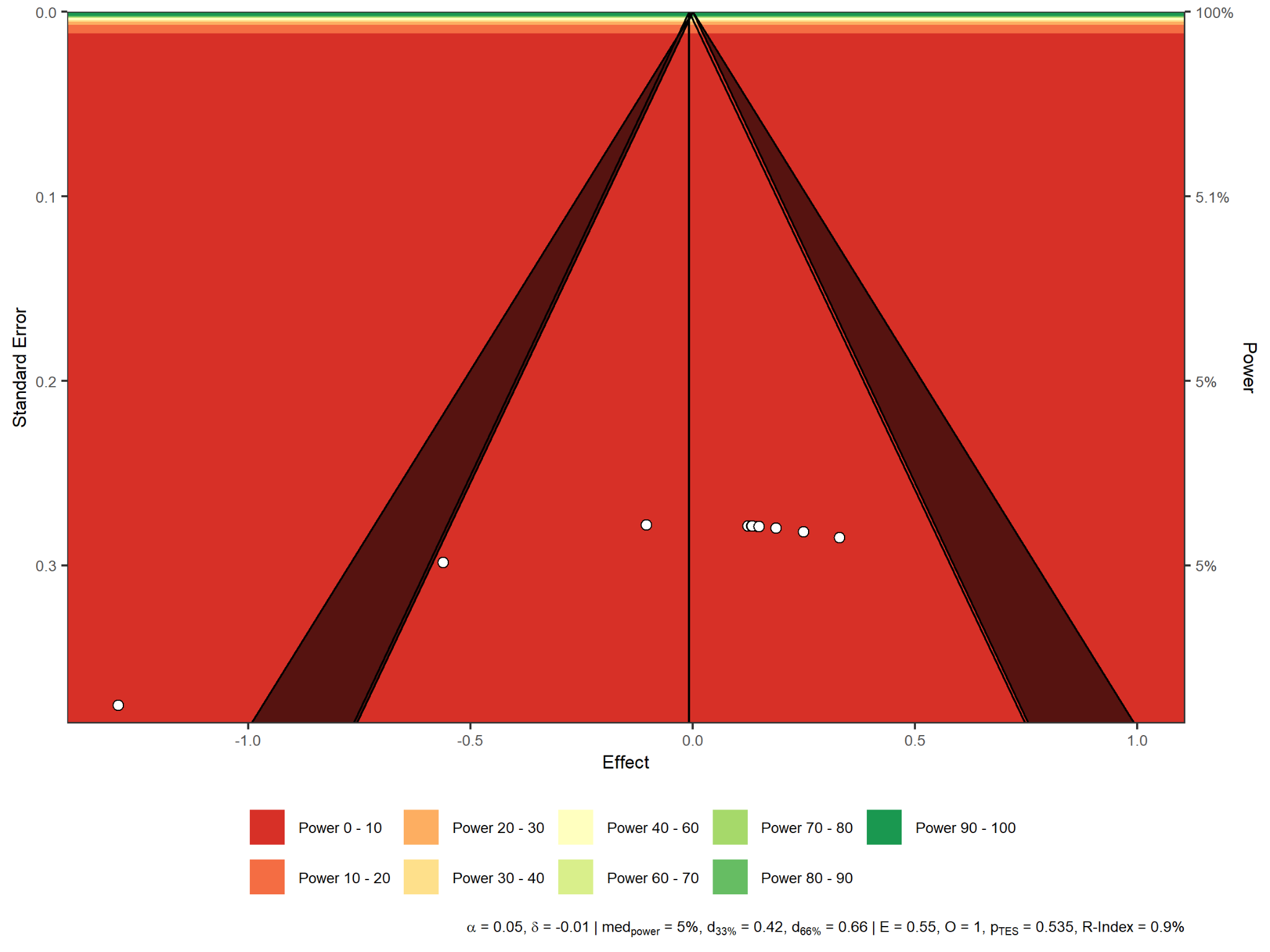 | **G**  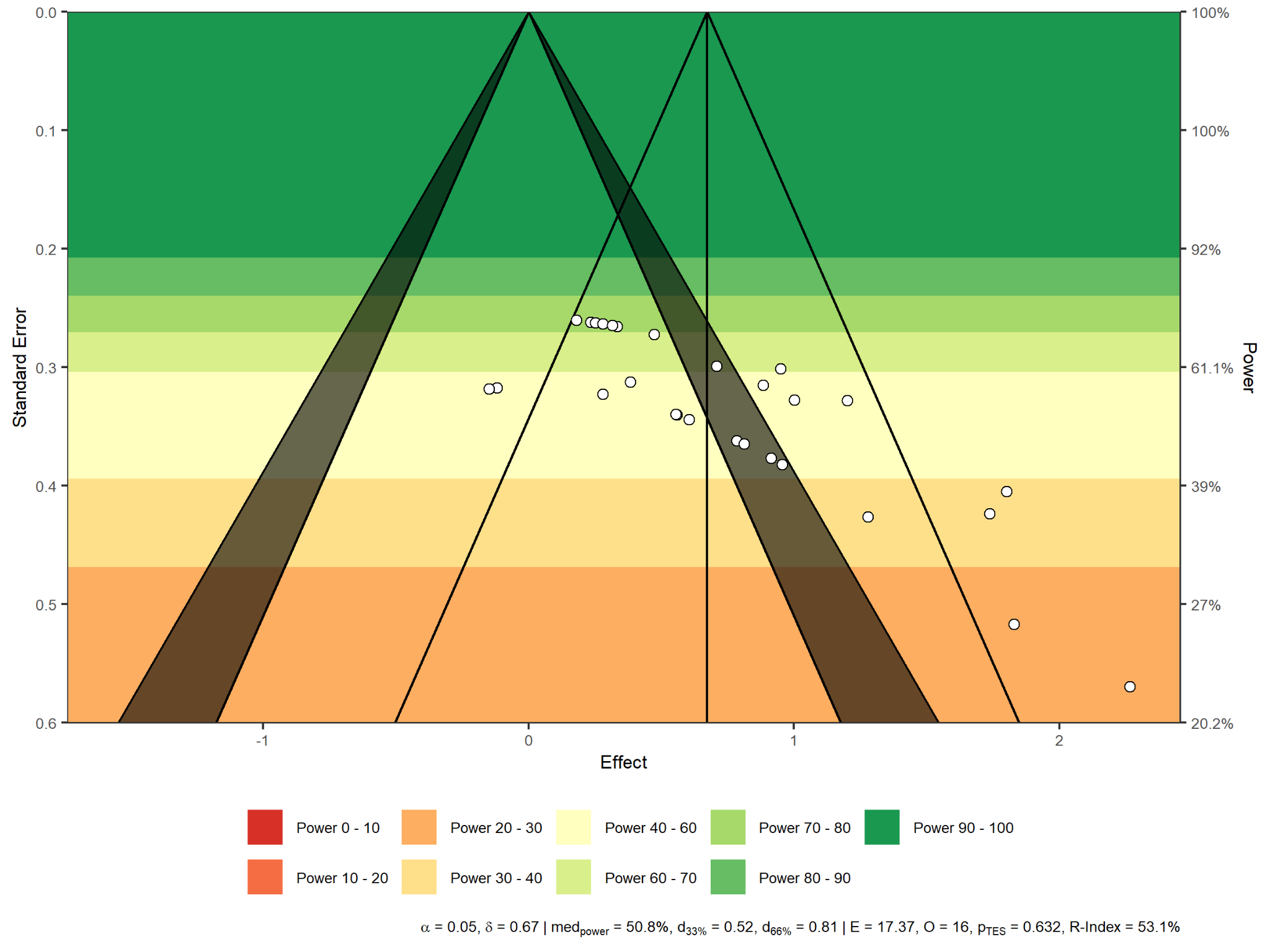 | **H**  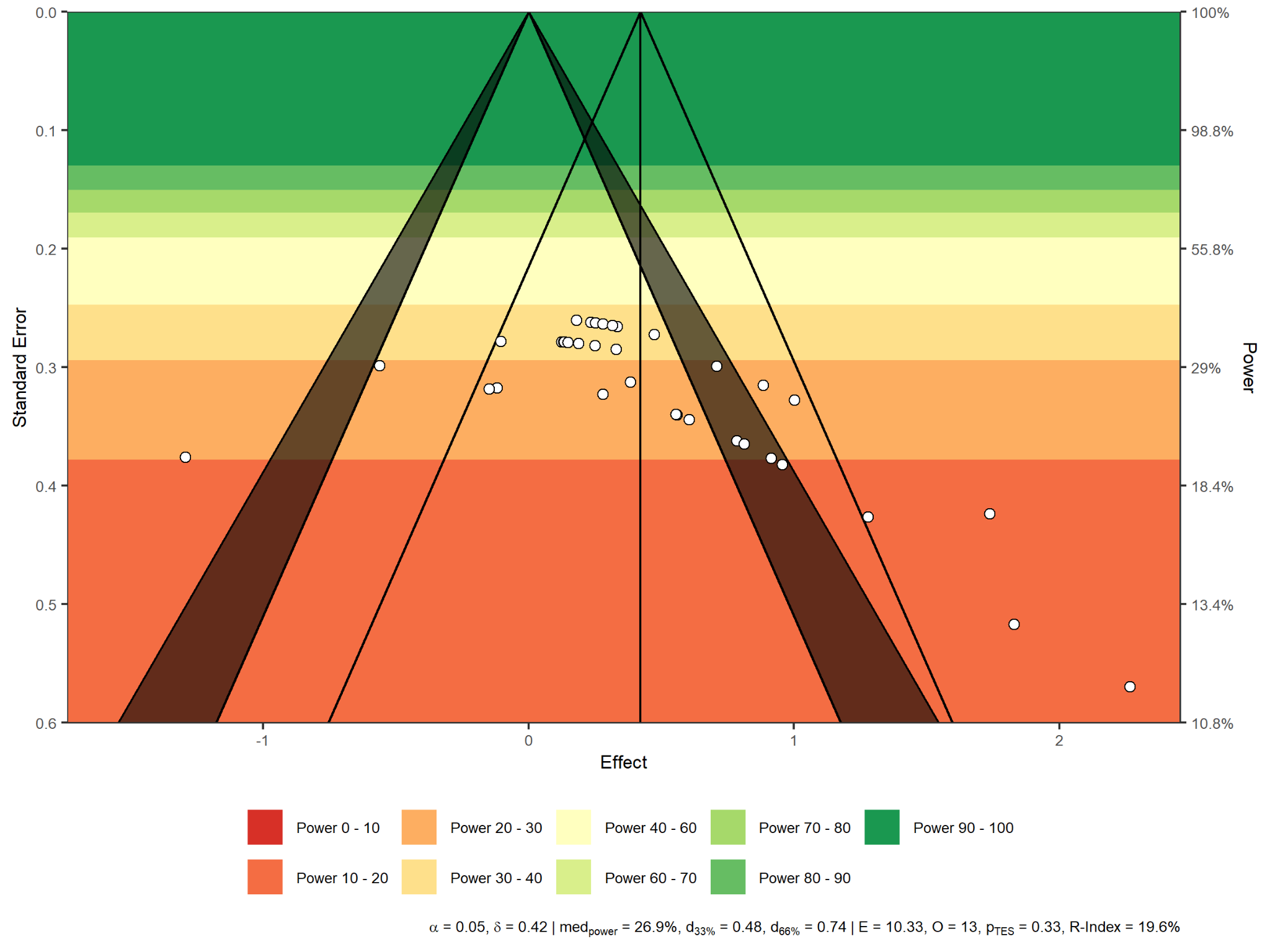 |
| **I**  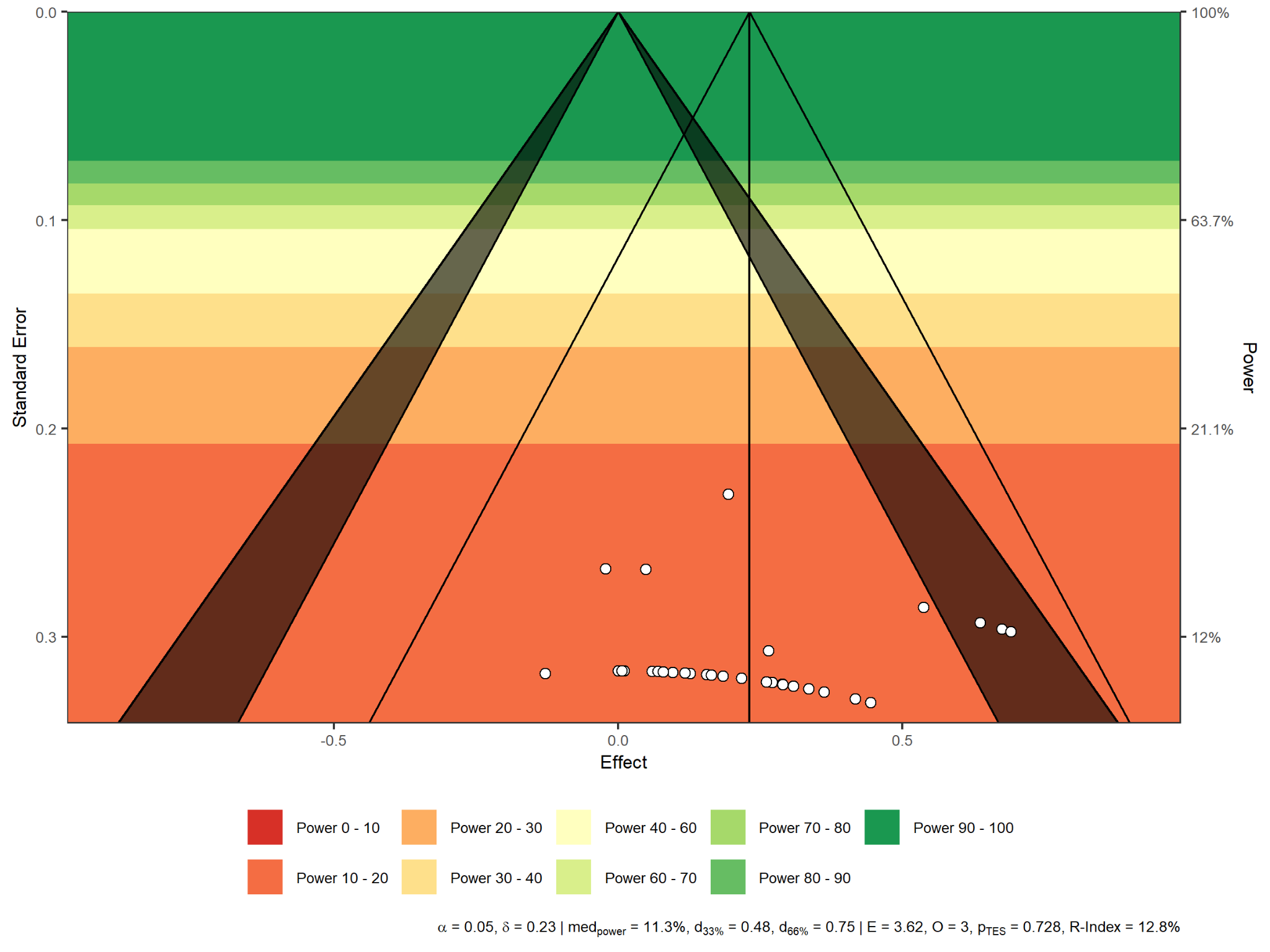 | **J**  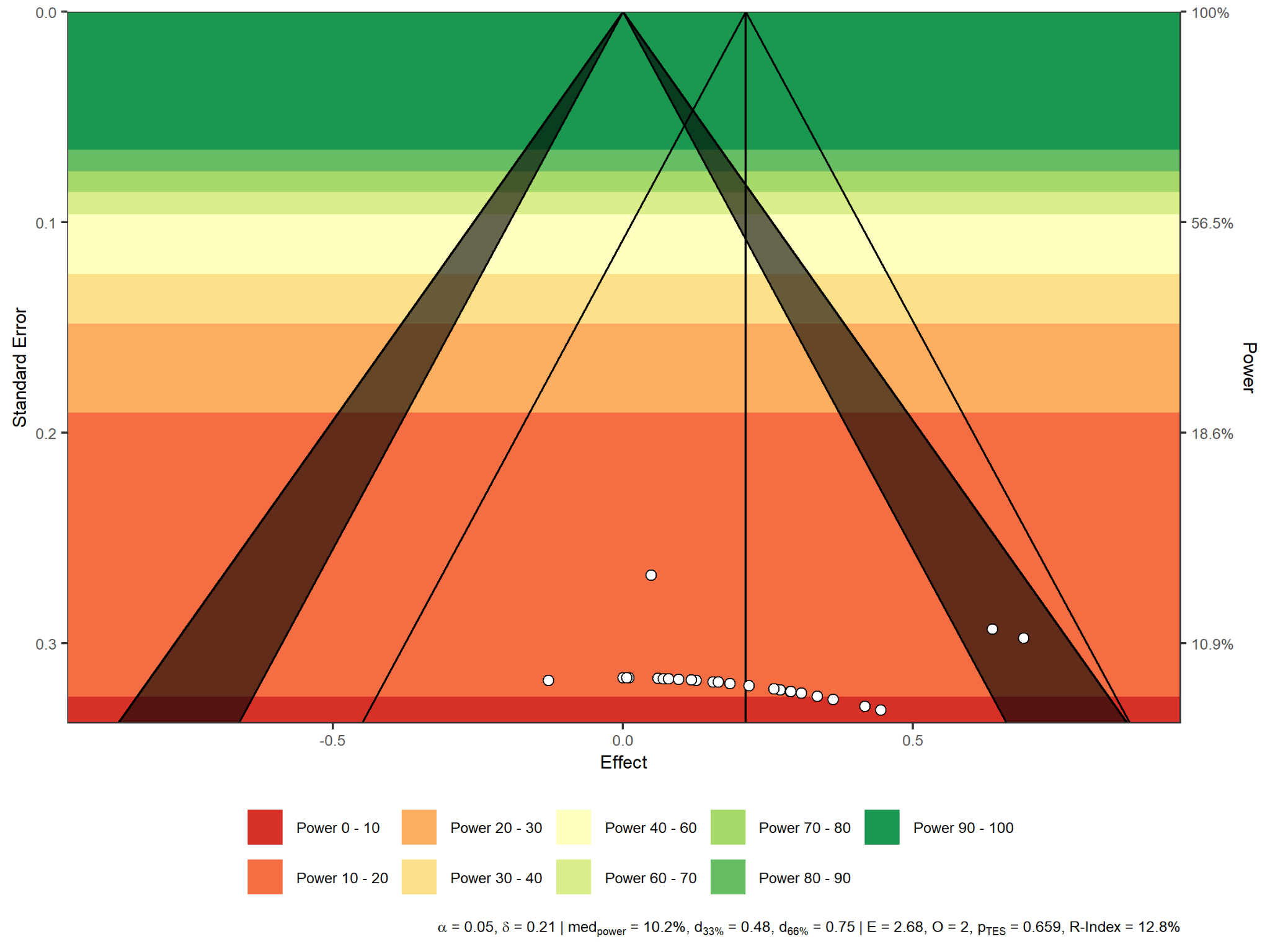 | **K**  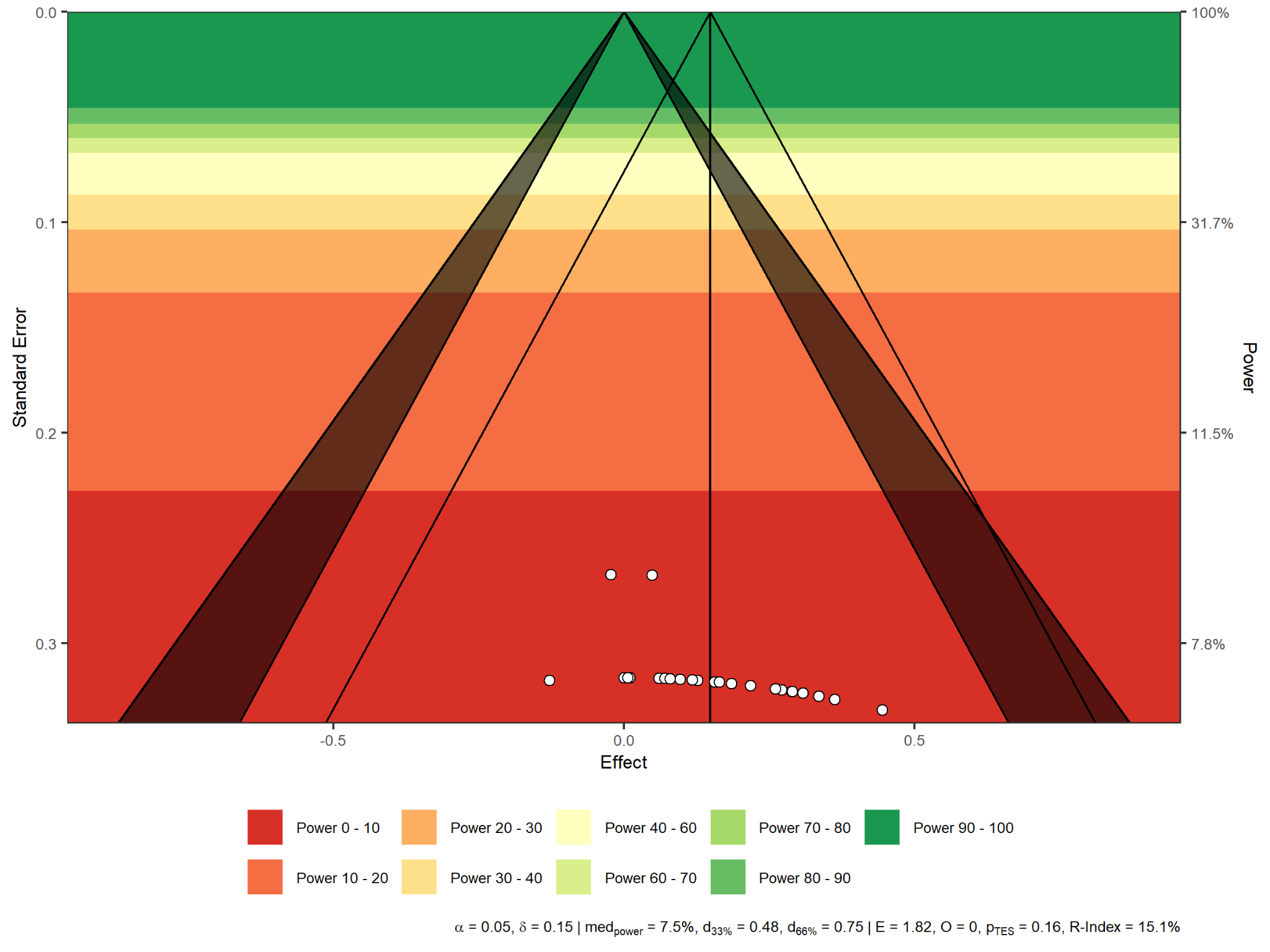 | **L**  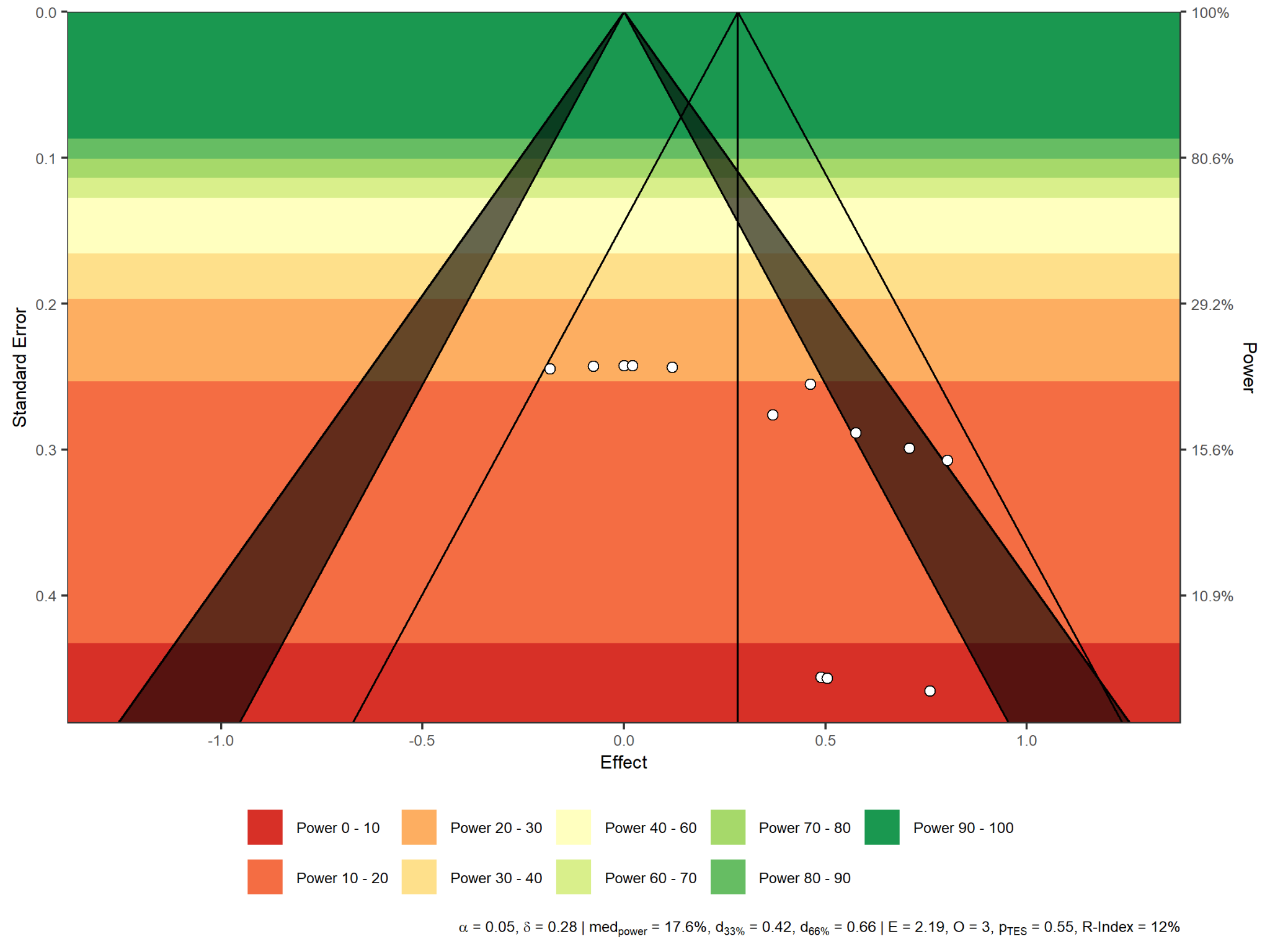 |
| **M**  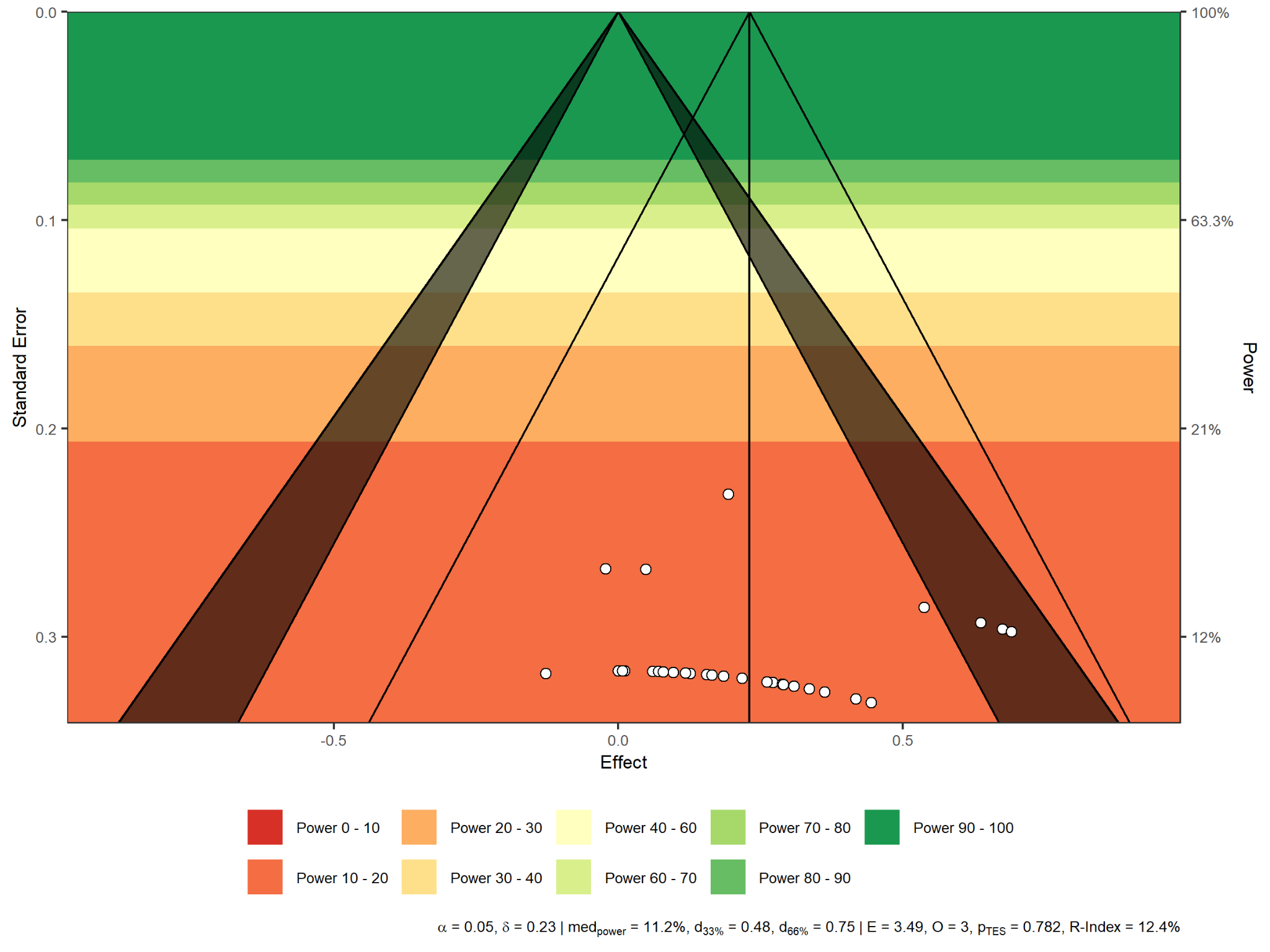 | **N**  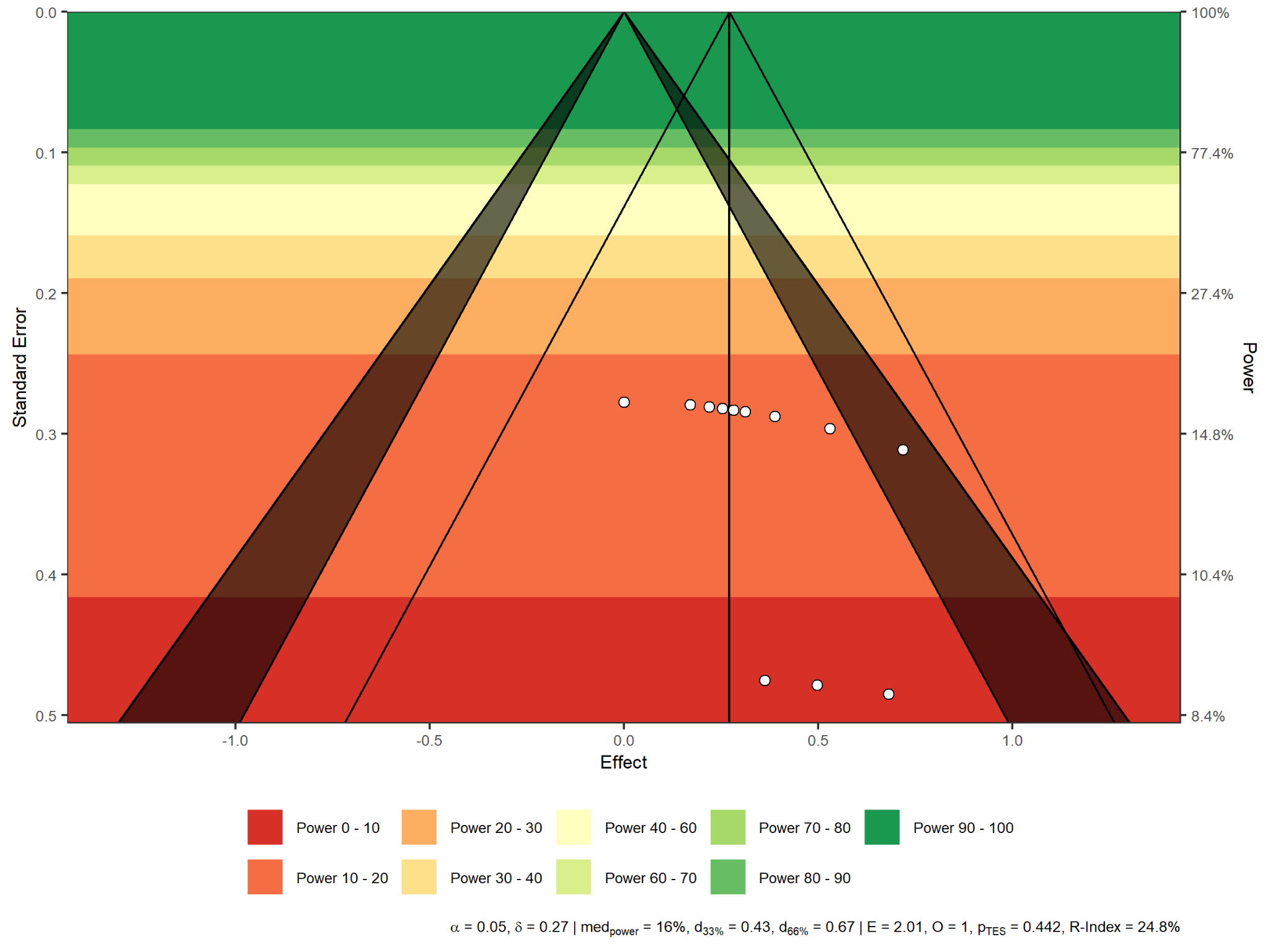 | **O**  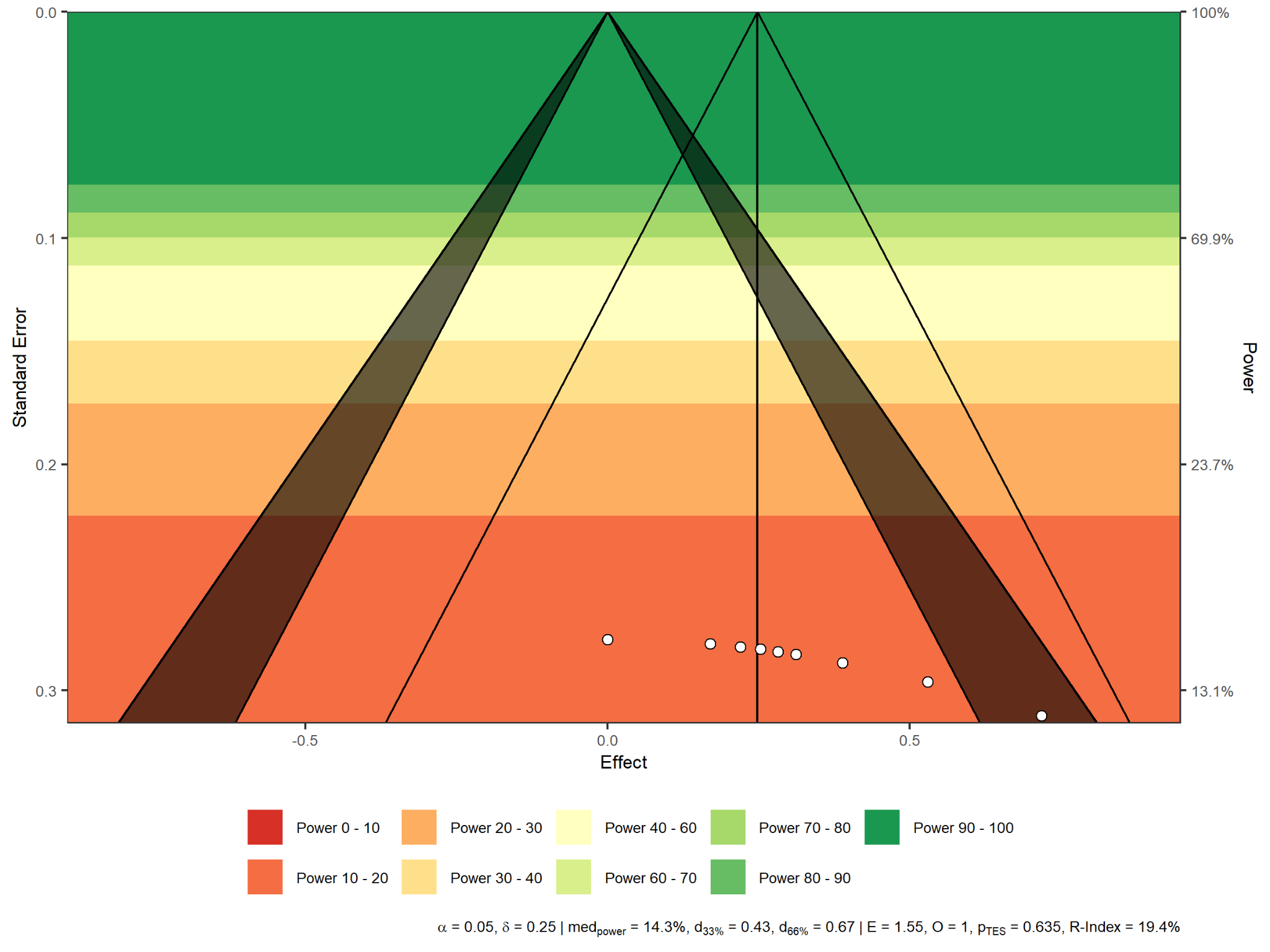 | **P**  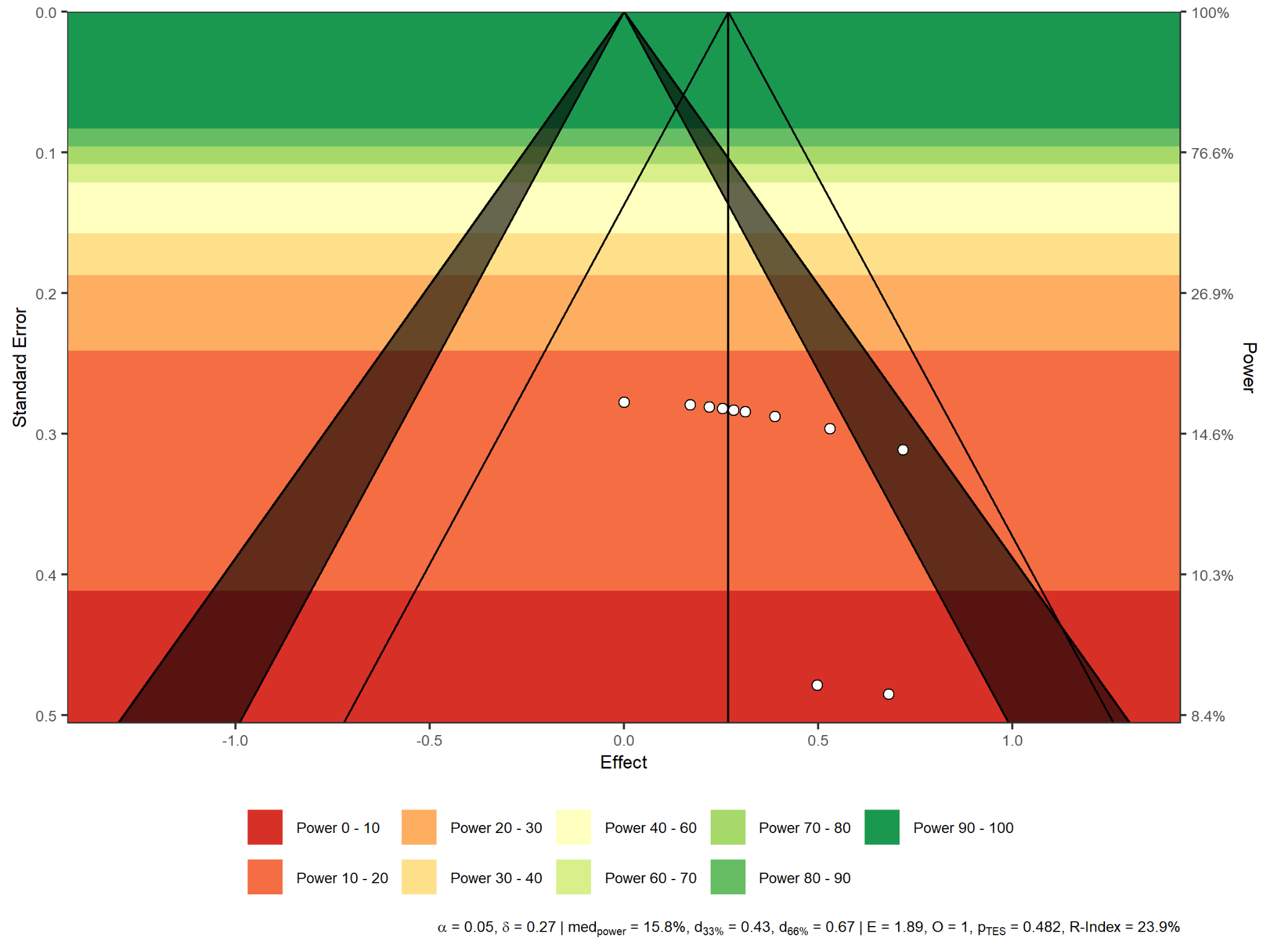 |
| **Q**  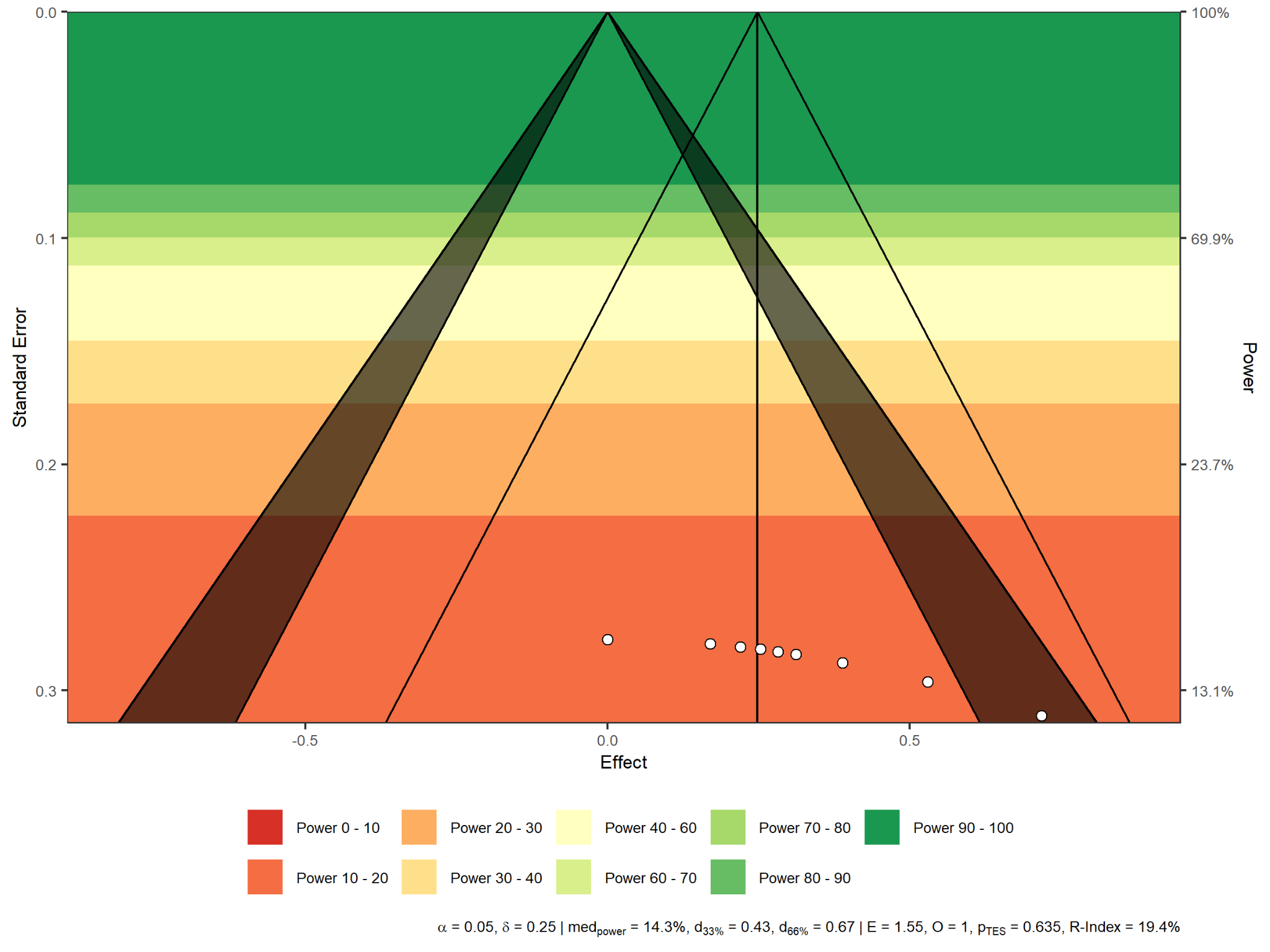 | **R**  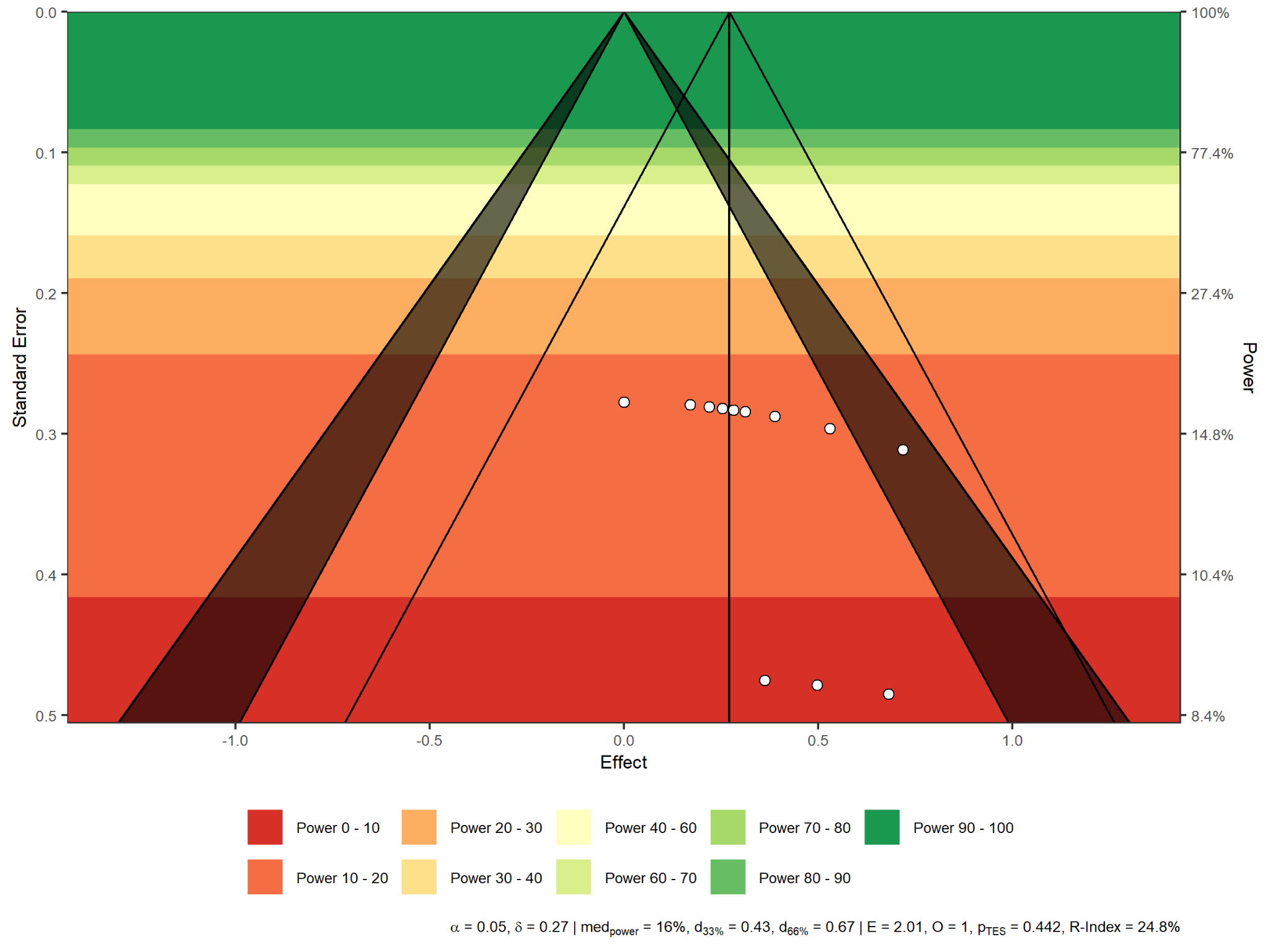 | **S**  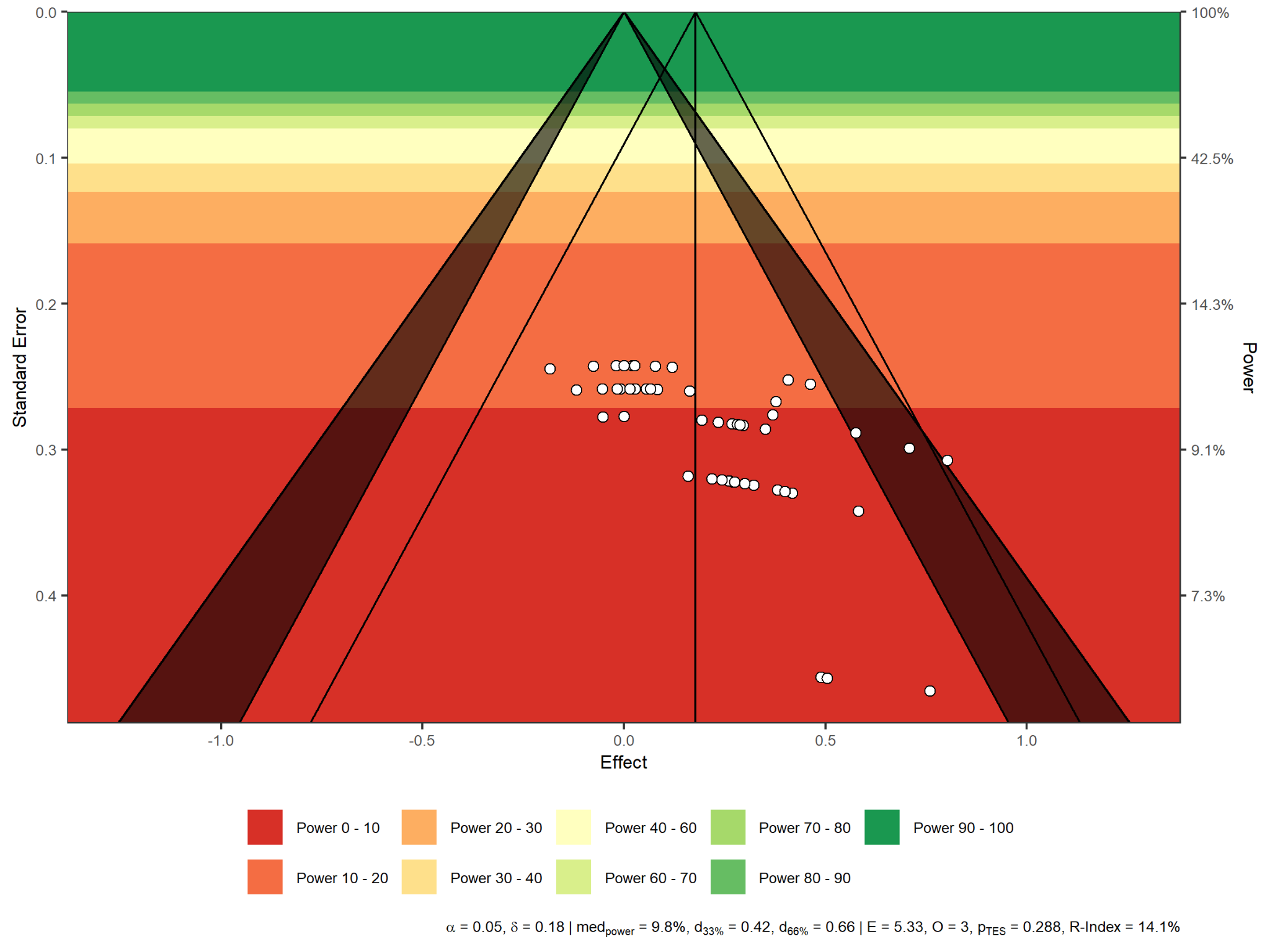 | **T**  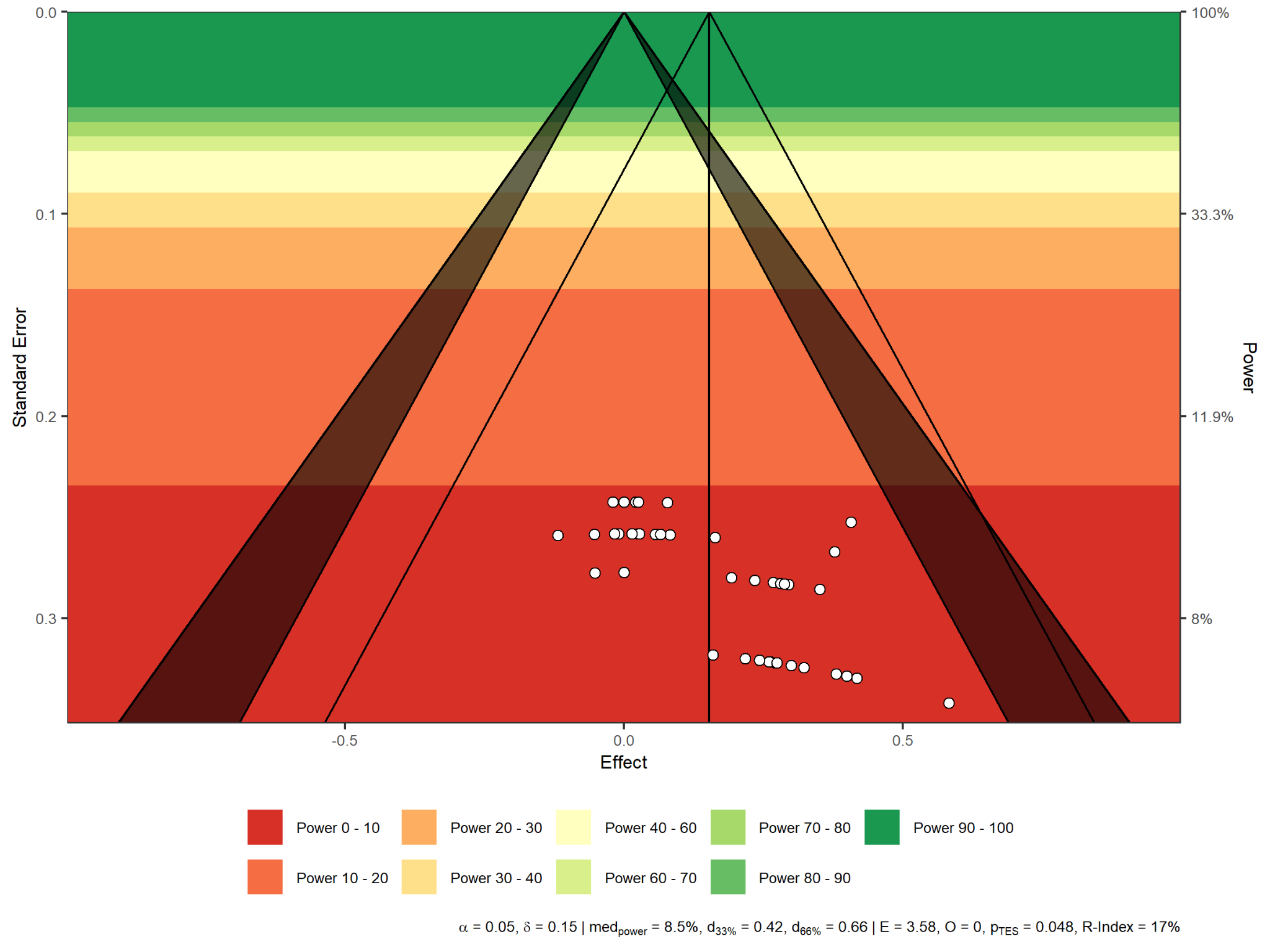 |
| **U**  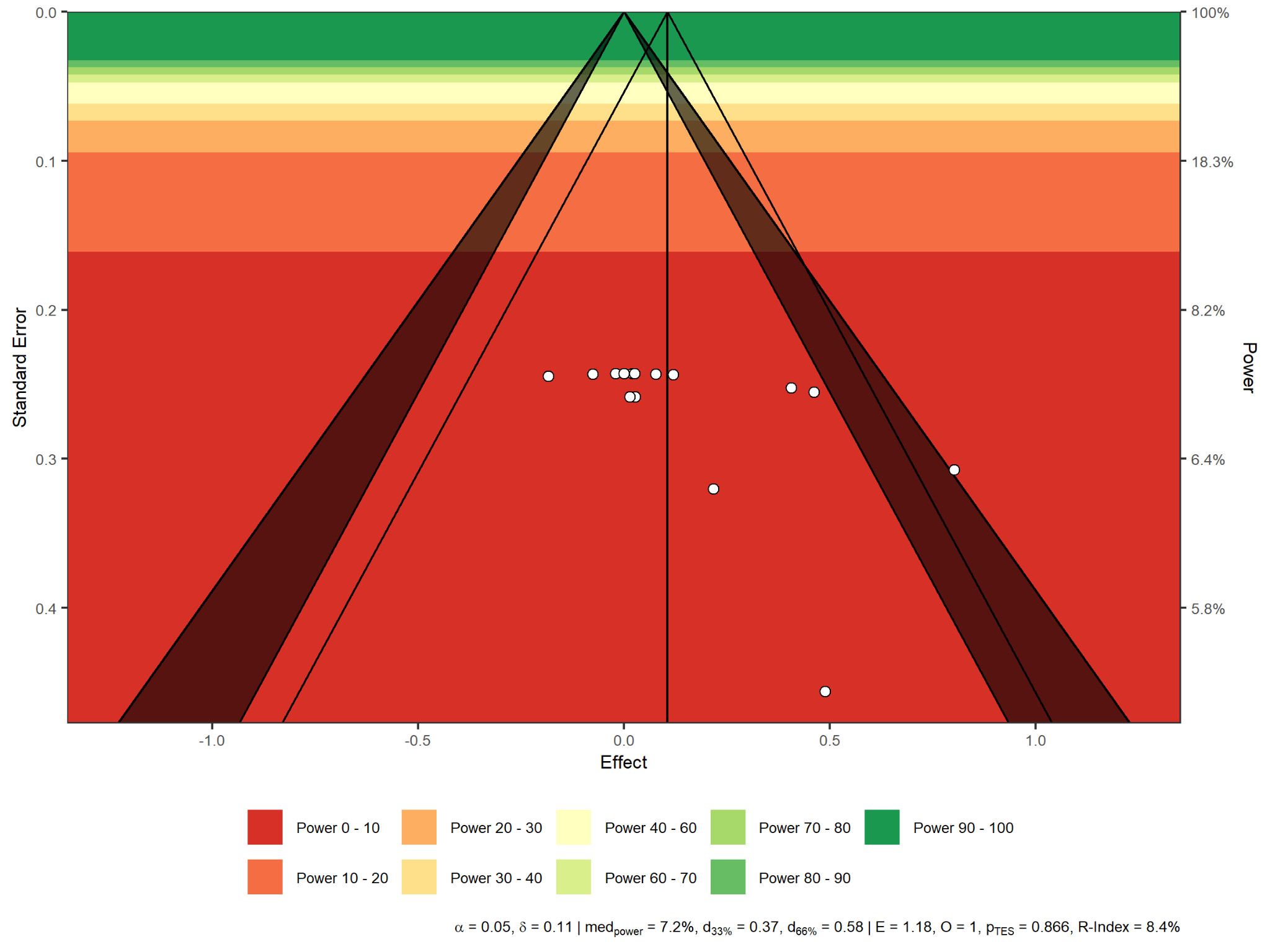 | **V**  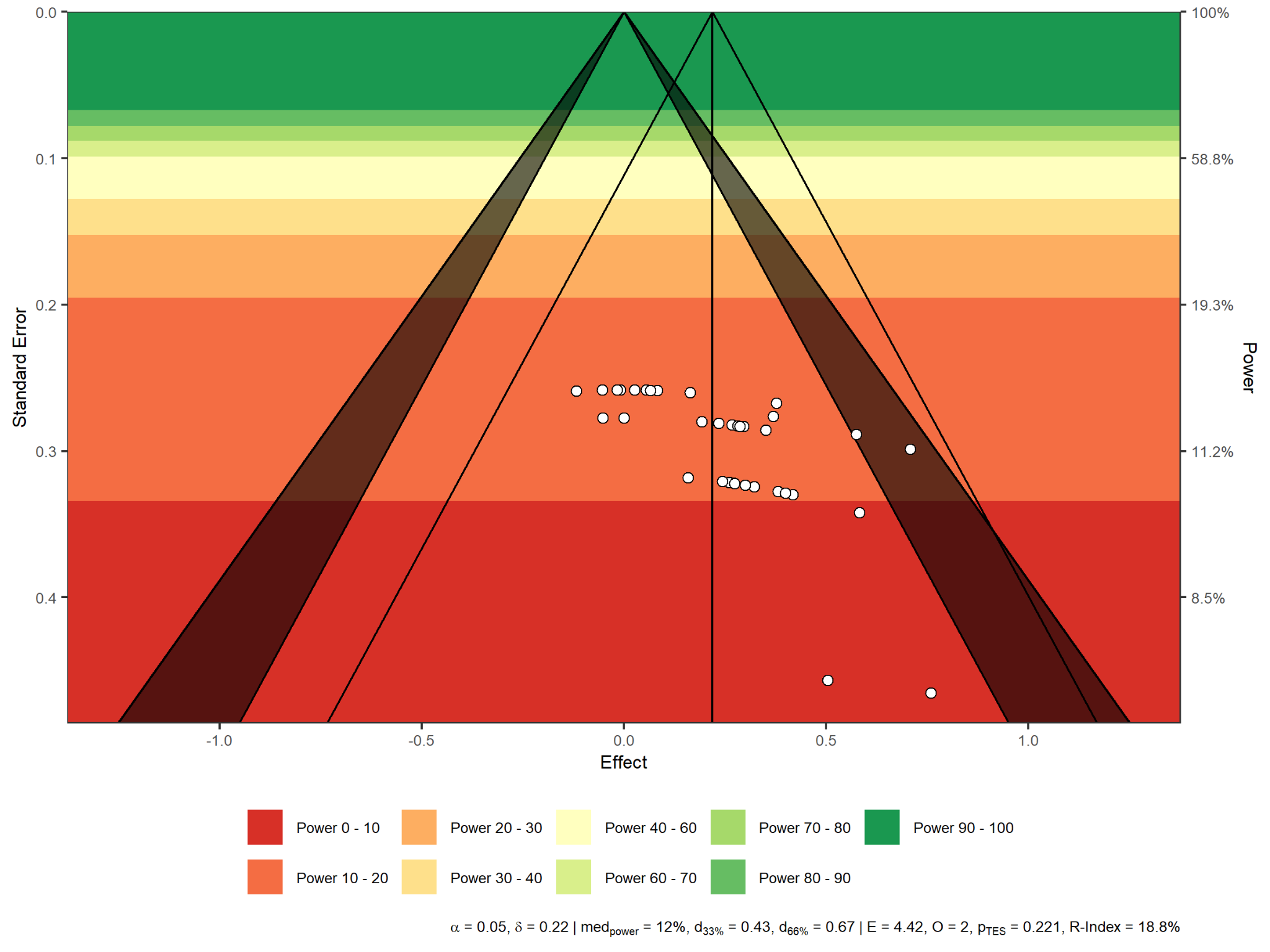 | **W**  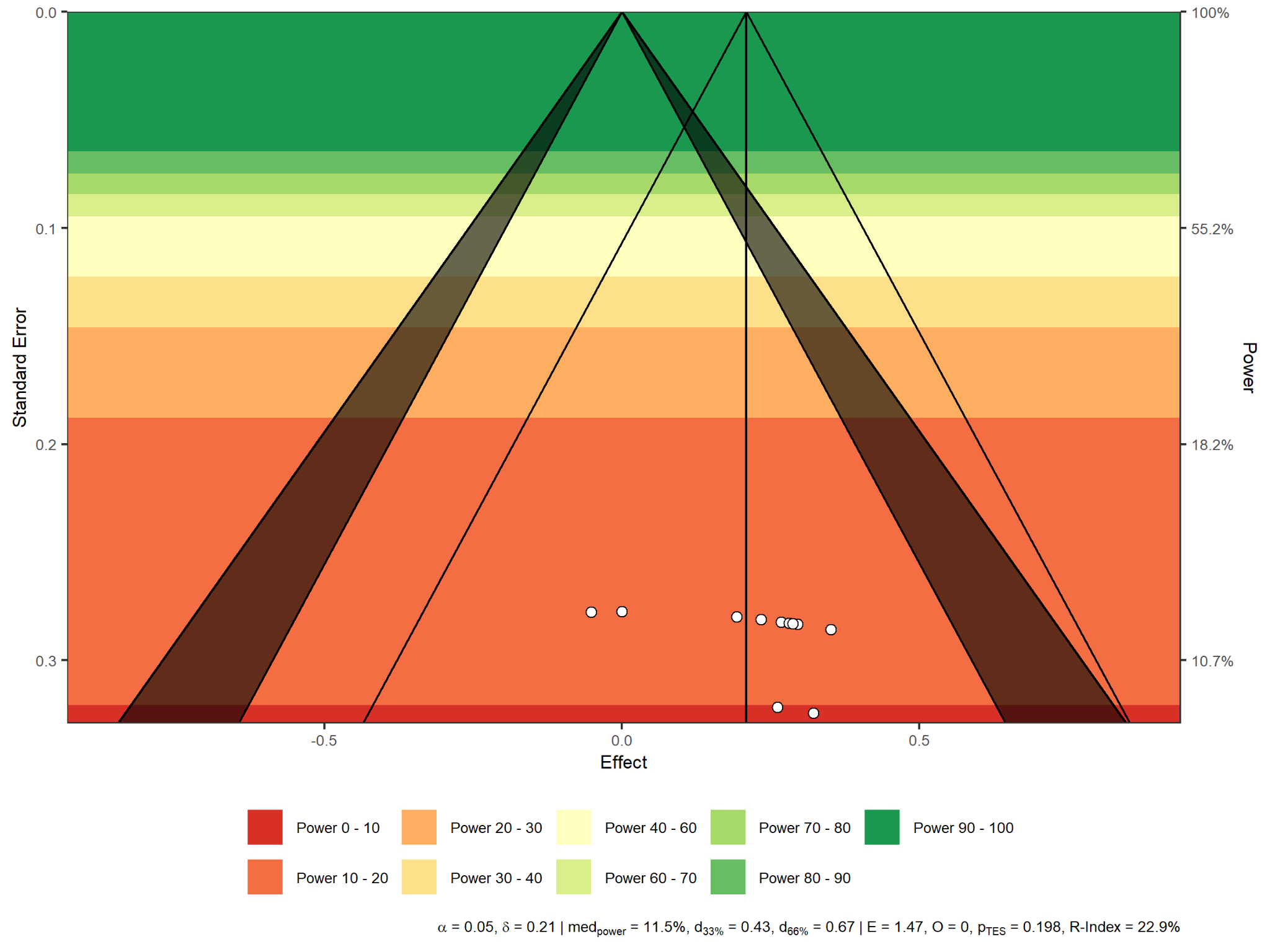 | **X**  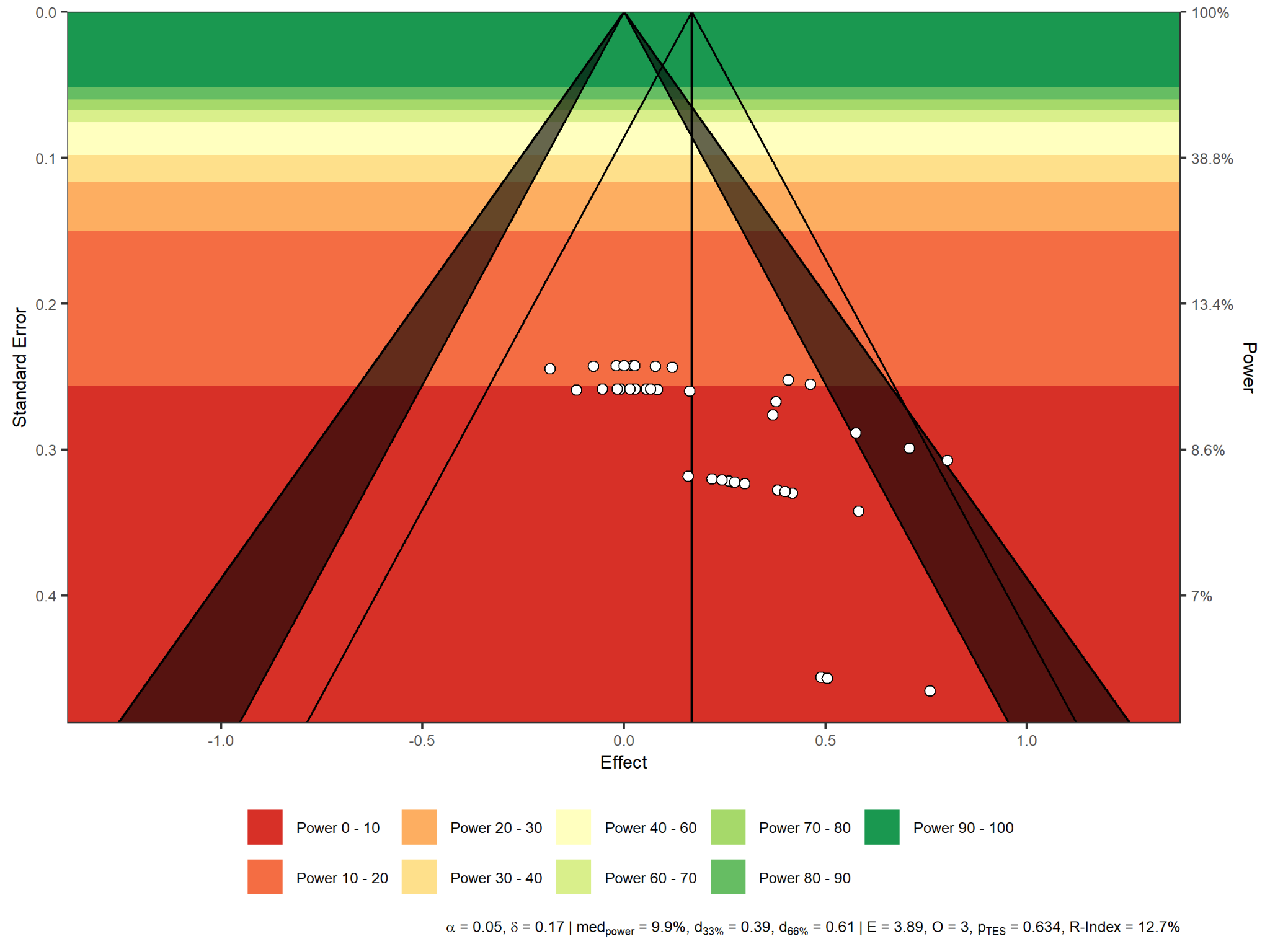 |
|  | **Y**  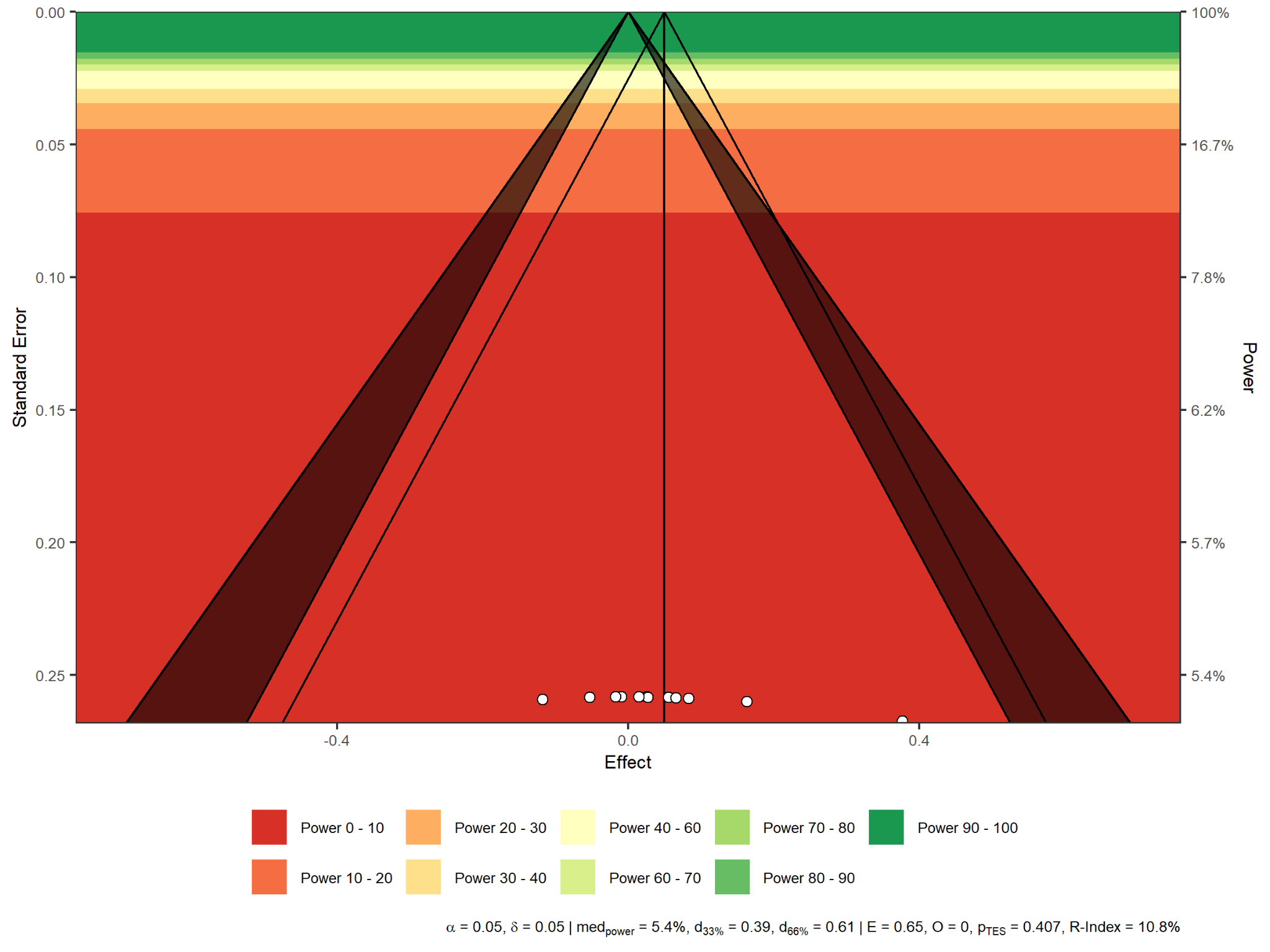 | **Z**  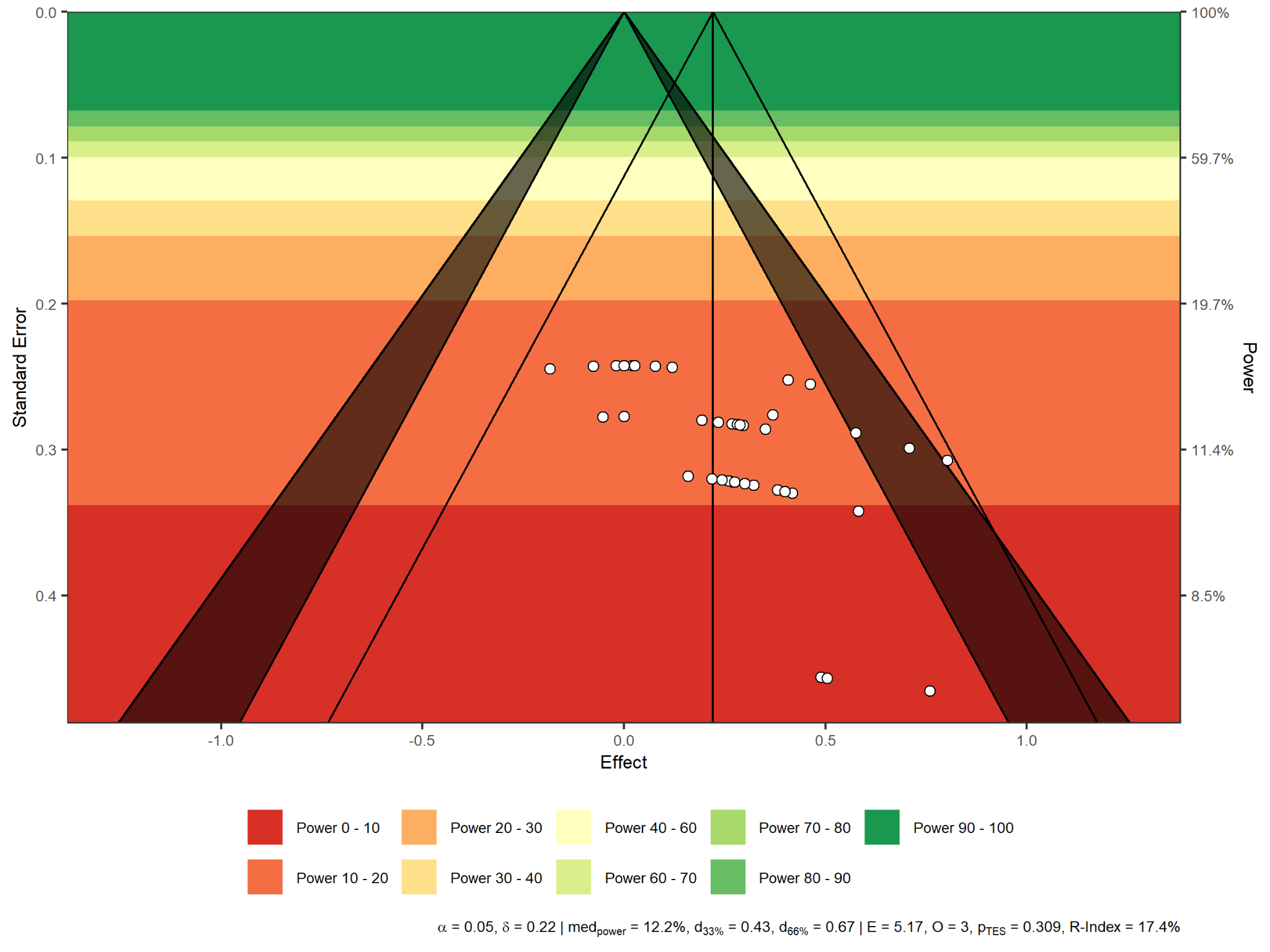 |  |

**Notes:** Open circles represent individual effect sizes. The colored background bands indicate statistical power levels from low power (red) to high power (green). Black funnel contours show expected precision regions around the pooled effect. Panels A–H show EF analyses: overall, 3 mg/kg, 4 mg/kg, endurance, neuromuscular, hormonal verification, no/cycle verification, and 60 min. Panels I–M show HC/OCP analyses: overall, 60 min, neuromuscular, no/cycle verification, and ≥5 mg/kg. Panels N–R show LFP/PO analyses: overall, 3 mg/kg, neuromuscular, hormonal verification, and 60 min. Panels S–Z show LP/ML/MLP analyses: overall, 3 mg/kg, endurance, neuromuscular, hormonal verification, no/cycle verification, 30 min, and 60 min. Abbreviations: EF, early follicular phase; LFP/PO, late follicular/peri-ovulatory phase; LP/ML/MLP, luteal/mid-luteal/mid-luteal phase; HC/OCP, hormonal contraceptive/oral contraceptive users.

**Electronic Supplementary Material Appendix S11 (Modified PEDro assessment of the included randomized controlled trials)**

| Table A. PEDro assessment results of exercise performance researches | | | | | | | | | | | | | |
| --- | --- | --- | --- | --- | --- | --- | --- | --- | --- | --- | --- | --- | --- |
| Study | Item 1 | Item 2 | Item 3 | Item 4 | Item 5 | Item 6 | Item 7 | Item 8 | Item 9 | Item 10 | Item 11 | Item 12 | Total |
|  |  |  |  |  |  |  |  |  |  |  |  |  |  |
| Abumoh’d et al. (2024) | Yes | Yes | No | Yes | Yes | No | No | Yes | Yes | Yes | Yes | No | 7 |
| Ali et al. (2016) | Yes | Yes | No | Yes | Yes | No | No | Yes | Yes | Yes | Yes | No | 7 |
| Chen et al. (2015) | Yes | Yes | No | Yes | Yes | No | No | Yes | Yes | Yes | Yes | No | 7 |
| Clarke et al. (2019) | Yes | Yes | No | Yes | No | No | No | Yes | Yes | Yes | Yes | Yes | 7 |
| Jones et al. (2021) | Yes | Yes | No | Yes | Yes | No | No | Yes | Yes | Yes | Yes | No | 7 |
| Karayigit et al. (2021) | Yes | Yes | Yes | Yes | Yes | No | Yes | Yes | Yes | Yes | Yes | No | 9 |
| Lara et al. (2019) | Yes | Yes | No | Yes | Yes | No | No | Yes | Yes | Yes | Yes | Yes | 8 |
| Lara et al. (2020) | Yes | Yes | Yes | Yes | Yes | No | Yes | Yes | Yes | Yes | Yes | No | 9 |
| Lara et al. (2021) | Yes | Yes | Yes | Yes | Yes | No | Yes | Yes | Yes | Yes | Yes | Yes | 10 |
| Mendes et al. (2025) | Yes | Yes | Yes | Yes | Yes | No | No | Yes | No | Yes | Yes | No | 7 |
| Norum et al. (2020) | Yes | Yes | Yes | Yes | Yes | No | Yes | Yes | Yes | Yes | Yes | Yes | 10 |
| Ouergui et al. (2022) | Yes | Yes | No | Yes | No | No | No | Yes | Yes | Yes | Yes | Yes | 7 |
| Ouergui et al. (2023) | Yes | Yes | No | Yes | No | No | No | Yes | Yes | Yes | Yes | Yes | 7 |
| Robles-González et al. (2022) | Yes | Yes | Yes | Yes | Yes | Yes | Yes | Yes | Yes | Yes | Yes | No | 10 |
| Romero-Moraleda et al. (2019) | Yes | Yes | No | Yes | Yes | No | No | Yes | Yes | Yes | Yes | No | 7 |
| Santana et al. (2022) | Yes | Yes | No | Yes | Yes | No | No | Yes | Yes | Yes | Yes | No | 7 |
| Shlool et al. (2024) | Yes | Yes | No | Yes | Yes | No | No | Yes | Yes | Yes | Yes | No | 7 |
| Skinner et al. (2019) | Yes | Yes | Yes | Yes | Yes | No | No | Yes | Yes | Yes | Yes | Yes | 9 |
| Stojanović et al. (2020) | Yes | Yes | No | Yes | Yes | No | No | Yes | Yes | Yes | Yes | No | 7 |
| Suvi et al. (2017) | Yes | Yes | No | Yes | Yes | No | No | Yes | Yes | Yes | Yes | Yes | 8 |

**Electronic Supplementary Material Appendix S12 (Table. GRADE certainty assessment for overall, subgroup, and exploratory meta-analytic outcomes)**

| **Outcome** | **K** | **Certainty of Evidence Assessment** | | | | | **Hedges' g [95% CI] *** | **GRADE†** |
| --- | --- | --- | --- | --- | --- | --- | --- | --- |
|  |  | **Risk of Bias** | **Inconsistency** | **Indirectness** | **Imprecision** | **Others** |  |  |
| Primary outcome and menstrual-cycle/contraceptive strata | | | | | | | | |
| Overall exercise performance | K = 144 | Serious | Not serious | Not serious | Not serious | Publication bias | 0.37 [0.24, 0.50] * | ⨁⨁◯◯  Low |
| Early follicular (EF) | K = 44 | Serious | Serious | Not serious | Not serious | None | 0.42 [0.25, 0.59] * | ⨁⨁◯◯  Low |
| Late follicular / peri-ovulatory (LFP/PO) | K = 14 | Serious | Not serious | Not serious | Very serious | None | 0.48 [0.26, 0.71] * | ⨁◯◯◯  Very low |
| Luteal / mid-luteal (LP/ML/MLP) | K = 55 | Serious | Not serious | Not serious | Not serious | Publication bias | 0.35 [0.18, 0.53] * | ⨁⨁◯◯  Low |
| Hormonal contraceptive / oral contraceptive (HC/OCP) | K = 31 | Serious | Not serious | Not serious | Serious | None | 0.28 [0.00, 0.55] | ⨁⨁◯◯  Low |
| Dose analyses | | | | | | | | |
| EF - 3 mg/kg | K = 23 | Serious | Serious | Not serious | Serious | None | 0.45 [0.04, 0.85] * | ⨁◯◯◯  Very low |
| EF - 4 mg/kg | K = 14 | Serious | Not serious | Not serious | Very serious | None | 0.54 [0.02, 1.07] * | ⨁◯◯◯  Very low |
| EF - >=5 mg/kg | K = 7 | Serious | Serious | Not serious | Very serious | None | 0.65 [0.01, 1.30] * | ⨁◯◯◯  Very low |
| LP/ML/MLP - 3 mg/kg | K = 42 | Serious | Not serious | Not serious | Not serious | Publication bias | 0.21 [0.07, 0.36] * | ⨁⨁◯◯  Low |
| LP/ML/MLP - >=5 mg/kg | K = 13 | Serious | Serious | Not serious | Very serious | None | 0.31 [0.10, 0.52] * | ⨁◯◯◯  Very low |
| Exercise-type analyses | | | | | | | | |
| EF - Endurance / repeated-output | K = 19 | Serious | Serious | Not serious | Serious | None | 0.73 [0.45, 1.01] * | ⨁◯◯◯  Very low |
| EF - Neuromuscular / maximal-output | K = 25 | Serious | Serious | Not serious | Serious | None | 0.37 [0.12, 0.62] * | ⨁◯◯◯  Very low |
| LP/ML/MLP - Endurance / repeated-output | K = 17 | Serious | Not serious | Not serious | Very serious | None | 0.20 [-0.03, 0.42] | ⨁◯◯◯  Very low |
| LP/ML/MLP - Neuromuscular / maximal-output | K = 38 | Serious | Not serious | Not serious | Serious | None | 0.26 [0.11, 0.41] * | ⨁⨁◯◯  Low |
| Phase-verification analyses | | | | | | | | |
| EF - No verification / cycle counting | K = 33 | Serious | Not serious | Not serious | Serious | None | 0.68 [0.45, 0.91] * | ⨁⨁◯◯  Low |
| EF - Hormonal verification | K = 11 | Serious | Not serious | Not serious | Very serious | None | 0.07 [-0.31, 0.44] | ⨁◯◯◯  Very low |
| LP/ML/MLP - No verification / cycle counting | K = 42 | Serious | Not serious | Not serious | Not serious | Publication bias | 0.26 [0.07, 0.44] * | ⨁⨁◯◯  Low |
| LP/ML/MLP - Hormonal verification | K = 13 | Serious | Not serious | Not serious | Very serious | None | 0.23 [-0.02, 0.48] | ⨁◯◯◯  Very low |
| Timing-category analyses | | | | | | | | |
| EF - 60 min | K = 38 | Serious | Not serious | Not serious | Serious | None | 0.46 [0.19, 0.72] * | ⨁⨁◯◯  Low |
| EF - 45 min | K = 6 | Serious | Not serious | Not serious | Very serious | None | 0.97 [0.23, 1.72] * | ⨁◯◯◯  Very low |
| LP/ML/MLP - 60 min | K = 43 | Serious | Not serious | Not serious | Not serious | Publication bias | 0.28 [0.13, 0.43] * | ⨁⨁◯◯  Low |
| LP/ML/MLP - 30 min | K = 12 | Serious | Not serious | Not serious | Very serious | None | 0.05 [-0.28, 0.38] | ⨁◯◯◯  Very low |

**Notes:** K, number of effect sizes included in the pooled estimate; CI, confidence interval; EF, early follicular; GRADE, Grading of Recommendations Assessment, Development and Evaluation; HC/OCP, hormonal contraceptive/oral contraceptive; LFP/PO, late follicular/peri-ovulatory; LP/ML/MLP, luteal / mid-luteal / mid-luteal phase.

*: The pooled effect size (Hedges' g) was statistically significant (p < 0.05).

† GRADE certainty began as High because the evidence came from randomized controlled trials. Downgrading was cumulative across risk of bias, inconsistency, indirectness, imprecision, and other considerations: High = very confident; Moderate = moderately confident; Low = limited confidence; Very low = very limited confidence.

Evaluation rule used for this appendix: imprecision was judged primarily by effect-size K and the 95% CI. In general, K >= 40 with a CI excluding the null was not downgraded for imprecision; K = 20-39 or a CI close to/crossing the null was downgraded once; K < 20, single-study strata, or very wide/null-crossing intervals were downgraded twice. K counts effect sizes and should not be interpreted as independent participants because most source trials used crossover designs and contributed multiple outcomes.

Publication bias was not downgraded unless there was clear evidence of small-study effects. Subgroup-specific publication-bias tests were considered underpowered and were therefore not used as stand-alone downgrading evidence.
